# Effects of Prenatal Multiple Micronutrient Supplementation on Fetal Growth Factors: A Cluster-Randomized, Controlled Trial in Rural Bangladesh

**DOI:** 10.1371/journal.pone.0137269

**Published:** 2015-10-02

**Authors:** Alison D. Gernand, Kerry J. Schulze, Ashika Nanayakkara-Bind, Margia Arguello, Abu Ahmed Shamim, Hasmot Ali, Lee Wu, Keith P. West, Parul Christian

**Affiliations:** 1 Department of International Health, Center for Human Nutrition, Johns Hopkins Bloomberg School of Public Health, Baltimore, MD, United States of America; 2 Department of Nutritional Sciences, Pennsylvania State University, University Park, PA, United States of America; 3 The JiVitA Project, Gaibandha, Bangladesh; The University of Queensland, AUSTRALIA

## Abstract

Prenatal multiple micronutrient (MM) supplementation improves birth weight through increased fetal growth and gestational age, but whether maternal or fetal growth factors are involved is unclear. Our objective was to examine the effect of prenatal MM supplementation on intrauterine growth factors and the associations between growth factors and birth outcomes in a rural setting in Bangladesh. In a double-blind, cluster-randomized, controlled trial of MM vs. iron and folic acid (IFA) supplementation, we measured placental growth hormone (PGH) at 10 weeks and PGH and human placental lactogen (hPL) at 32 weeks gestation in maternal plasma (n = 396) and insulin, insulin-like growth factor-1 (IGF-1), and IGF binding protein-1 (IGFBP-1) in cord plasma (n = 325). Birth size and gestational age were also assessed. Early pregnancy mean (SD) BMI was 19.5 (2.4) kg/m^2^ and birth weight was 2.68 (0.41) kg. There was no effect of MM on concentrations of maternal hPL or PGH, or cord insulin, IGF-1, or IGFBP-1. However, among pregnancies of female offspring, hPL concentration was higher by 1.1 mg/L in the third trimester (95% CI: 0.2, 2.0 mg/L; p = 0.09 for interaction); and among women with height <145 cm, insulin was higher by 59% (95% CI: 3, 115%; p = 0.05 for interaction) in the MM vs. IFA group. Maternal hPL and cord blood insulin and IGF-1 were positively, and IGFBP-1 was negatively, associated with birth weight z score and other measures of birth size (all p<0.05). IGF-1 was inversely associated with gestational age (p<0.05), but other growth factors were not associated with gestational age or preterm birth. Prenatal MM supplementation had no overall impact on intrauterine growth factors. MM supplementation altered some growth factors differentially by maternal early pregnancy nutritional status and sex of the offspring, but this should be examined in other studies.

**Trial Registration:**

ClinicalTrials.gov NCT00860470

## Introduction

Intrauterine growth restriction remains a widespread public health issue across the globe [1]. Pooled results of over a decade of prenatal multiple micronutrient (MM) supplementation trials show a 54 (95% CI: 45–64) g gain in birth weight and a 17% reduced risk of small-for-gestational age (RR: 0.83; 95% CI: 0.73–0.95) for MM vs. iron-folate (IFA) supplementation [2]. Our recent trial of MM vs. IFA supplementation beginning in early pregnancy showed that birth weight and other measures of birth size were higher in offspring of mothers receiving MM due to a mean of 0.3 weeks longer length of gestation [3].

Little is known about what mechanisms underlie such an effect, but hormones and growth factors that regulate fetal growth are clear targets for research [4, 5]. Insulin and proteins in the insulin-like growth factor (IGF) family are some of the dominant regulators of fetal growth [6, 7]. Further, the placenta is an important regulator of fetal growth, synthesizing both human placental lactogen (hPL) and placental growth hormone (PGH) to impact maternal energy regulation. PGH is somatotrophic [8] and concentrations are decreased in maternal plasma of infants with IUGR [9]. Both hPL and PGH are believed to create peripheral insulin resistance in the mother that allows for preferential glucose supply to the fetus [10]. Insulin and IGF-1 are structurally homologous and are likely the most prominent endocrine regulators of fetal growth [10], while IGF binding protein-1 (IGFBP-1) binds to IGF-1 to limit its activity. Cord concentrations of IGFBP-1 are inversely associated with birth weight [11].

Relationships between micronutrients and growth factors have been examined in animal models and cellular research. Zinc and calcium have well-established roles in insulin synthesis and secretion [12] and circulating IGF-1 is lower from zinc deficiency [13]. Vitamin D increases IGF-1 and IGFBP-3 production [14]; vitamin E improves insulin sensitivity [15]; and retinoic acid [16] and vitamin D [17] each stimulate hPL production in trophoblast cells of healthy human placentas. These factors have rarely been studied in human subjects in nutritionally disadvantaged populations. An observational study in Pakistan found no correlation between cord zinc or ferritin and placental expression of IGF-1 or IGF-2 [18], but recently a trial in Burkina Faso found higher IGF-1 in the cord blood of male (but not female) newborns of MM vs. IFA supplemented mothers [19].

There is limited understanding of the potential for prenatal micronutrient supplementation to enhance in utero growth through changes in growth factors, especially in settings with high rates of malnutrition. Thus, we examined the impact of MM on key fetal growth factors in the context of a randomized trial of MM compared to IFA in rural Bangladesh. Further, we assessed the relationship between these growth factors and placental weight, infant size, and gestational age at birth.

## Methods

This study involved a sub-sample of pregnant women (n = 500) participating in a double-masked, cluster-randomized controlled trial (n = 44,567) of daily MM or IFA (standard of care) supplementation to reduce infant mortality [3]. The MM supplement tested contained 15 vitamins and minerals and was similar to the formulation recommended by UNICEF and WHO called UNIMMAP[20]. Iron (27 mg) and folic acid (600 μg) amounts were the same in both groups. The MM also included vitamin A (770 μg retinol equivalents), vitamin D (5 μg), vitamin E (15 mg), thiamin (1.4 mg), riboflavin (1.4 mg), niacin (1.4 mg), vitamin B_12_ (2.5 mg), vitamin B_6_ (1.9 mg), vitamin C (85 mg), zinc (12 mg), iodine (220 μg), copper (1000 μg), and selenium (60 μg). The trial was conducted in rural northwestern Bangladesh where the ~435 km^2^ study area was divided into 596 sectors of comparable size that were used as units of randomization (additional details and trial protocol are published [3]). In the study area, all non-pregnant, married women of reproductive potential were visited every 5 weeks by a study worker and offered a urine-based pregnancy test if they reported no menstruation in the last 30 days. All women with a positive pregnancy test were invited to enroll in the parent trial and our staff read the entire consent form aloud. Women were given time to consider the response and discuss with the head(s) of the household. Consent statements were read to the woman, and due to low literacy and limited use of signatures or thumb prints in the culture, a verbal response was given and recorded by our staff in the presence of a second witness. Our process of verbal consent was approved by the ethics committees (see below).

A substudy was conducted in 64 contiguous sectors (32 per group) to assess the changes in maternal vitamin and mineral status due to supplementation. The area was purposely selected to be accessible by road but similar to the parent trial across several socio-demographic and geographic factors. Maternal venous blood was obtained at 10 and 32 weeks of gestation, among numerous other measurements. Approximately half of this substudy area (31 sectors) was selected for an additional intensive home-based cord blood collection protocol. A primary outcome of this study was concentrations of maternal and fetal growth factors, assessed at 10 and 32 weeks gestation (maternal) and at birth (fetal, from cord blood). From February 2009 to April 2010, newly pregnant women in the selected substudy area were asked for consent (by the same process described for the parent trial) for cord blood collection after enrolling in the parent study at a median of 9.6 (IQR 7.7, 11.7) weeks gestation. Final births from enrolled women, and the end of follow up for this substudy, occurred in November 2010. Those >28 weeks gestation at the time of enrollment were excluded from the cord blood substudy. Sample size (n = 310 cord blood samples) was set to detect differences in micronutrient status.

Local field workers met women weekly to provide the micronutrient supplements from enrollment to 3 months postpartum; supplements were self-administered daily. Trained data collectors measured women in their homes at the time of substudy enrollment. A questionnaire was used to collect self-reported data on pregnancy history, diet, strenuous work, morbidities, socio-demographics, and other maternal and household factors. Study staff trained to do ultrasound assessments (SonoSite Titan, SonoSite, Bothel, WA) measured crown-rump length transabdominally at <15 wk to estimate gestational age; date of last menstrual period was used for gestational age estimation if ultrasound was unavailable (n = 44). Before supplementation (~10 wks) and at 32 weeks gestation, the technicians collected 7 mL of maternal venous blood. A drop was used to measure hemoglobin on the spot with a HemoCue photometer (Hb-301, HemoCue). Blood collection tubes were immediately stored in padded, insulated cold boxes with ice packs until technicians returned to the central lab for processing. Processing occurred approximately 1 to 6 hours after collection due to field workers traveling to several homes in difficult to reach rural areas in the same day before returning to the lab.

Because over 90% of women deliver at home in this rural setting, we developed an extensive labor tracking system to allow women or a close family member or friend to notify us of the beginning of labor. Trained nurse midwives were on call 24 hours a day to go to the home (or other delivery site) when notified. Immediately following birth and separation of the infant and placenta, cord venous blood (7 mL) was collected by the trained nurse midwives from the placental side with a syringe; transferred to a 7 mL blood collection tube; put in a padded, insulated cold box with ice packs; and transported to the field-based laboratory for processing. Cord blood processing typically occurred 1–2 hours after collection. Maternal and cord blood tubes were centrifuged and separated plasma was measured into multiple aliquots and immediately stored in liquid nitrogen until received at our US laboratory. Thereafter, plasma was stored at -80°C until assayed, without freeze-thaw cycles.

At birth, we weighed trimmed, drained placentas (iBalance 2500, Phoenix, AZ) and newborns (Seca Scales, Columbia, MD) on separate scales [21]. Data was collected on newborn length, head circumference, and mid-upper arm circumference (MUAC) by trained anthropometrists as part of the parent trial.

We have previously published analyses using this same substudy cohort of 500 enrolled women, and some additional details of the methods, not relevant here, can be found in those papers [21, 22]. The sample size for analysis is slightly different in each paper due to the completeness of relevant data in each investigation (e.g. the previous papers used data collected at 20 weeks gestation). For the benefit of the reader, we chose to present important maternal characteristics in this paper, even though some of the same characteristics can be found in the other publications (with a slightly different sample size due to the *n* for final analysis).

### Laboratory methods

hPL was run using ELISA kits purchased from IBL-America (Minneapolis, MN), with an inter-assay CV of 17%. Concentrations were too low at 10 weeks to be reliably detected and thus only 32 week samples were assayed. PGH was run using ELISA kits purchased from MyBiosource.com, with an inter-assay CV of 14% (assayed in both 10 and 32 week gestation samples). IGF-1 was analyzed using an automated chemiluminescent immunoassay system (Immulite 1000, Siemens Diagnostics). Inter-assay CV was 9.5% for pooled plasma run as a quality control. Insulin and IGFBP-1 were each assessed using a high sensitivity ELISA kit (Alpco Diagnostics, Salem NH). Insulin had an inter-assay CV of 6.2% for a pooled plasma sample; IGFBP-1 had an inter-assay CV of 4.6% for a quality control sample provided by the manufacturer. Lab technicians were blinded to supplementation group.

### Statistical analysis

Small-for-gestational-age (<10^th^ percentile) and birth weight z scores were calculated by a gestational age- and sex-specific birth weight reference [23]. Low birth weight was defined as <2.5 kg and preterm was a live birth at <37 completed weeks gestation. We used the chi-squared test and t test to examine the differences in baseline characteristics by treatment group for categorical and continuous variables, respectively. Spearman’s rank was used to test the correlations between growth factors. Distributions of PGH, insulin, and IGFBP-1 were right skewed and therefore natural log (ln) transformed for regression models. We used linear regression models to test the effect of supplementation on fetal growth hormones with generalized estimating equations (GEE) to account for cluster randomization. Power to detect the differences we found (at alpha = 0.05) were as follows: hPL = 0.50, PGH = 0.05, insulin = 0.10, IGF-1 = 0.22, IGFBP-1 = 0.05. We also tested for effect modification of the supplementation effect by infant sex and maternal parity, age, early pregnancy BMI, and height by the likelihood ratio test (α <0.10). Participants were analyzed in the original, assigned supplement group.

We used multiple linear regression models to test the association between fetal growth factors and placental weight, birth weight z score, length, head circumference, chest circumference, and mid-upper arm circumference. We used multiple log-binomial regression models to estimate the association between fetal growth factors and risk of preterm and SGA. We adjusted for potential confounders selected due to associations with our exposures and outcomes of interest: maternal BMI, height, parity, age, education, and supplementation group. We present results from untransformed variables, except for insulin as an outcome, in all regression models to improve interpretability as results were similar and model fit was good when using untransformed compared to ln transformed variables. For the effect of MM on insulin, we report the percent change from models using ln transformed concentration.

We tested the sensitivity of our results by excluding twins (n = 4); extreme statistical outliers (n = 1 for PGH, n = 7 for insulin; n = 1 for IGF; n = 5 for IGFBP-1) or low undetectable concentrations for insulin (n = 2) or IGF (n = 18), which did not meaningfully change results and thus are not presented. As well, there were no meaningful differences in results when restricting analyses of maternal samples to pregnancies ending in singleton, live births (n = 387). All data analysis was conducted with Stata 13.0 (College Station, TX) and statistical significance was considered at p<0.05.

### Ethics statement

The trial and substudies received ethical approvals from the Institutional Review Board (IRB) at the Johns Hopkins School of Public Health, Baltimore, MD and the Bangladesh Medical Research Council (BMRC), Dhaka, Bangladesh. The current study was approved on December 11, 2008 by the Johns Hopkins’ IRB and on December 1, 2008 by the BMRC. The parent trial is registered on clinicaltrials.gov (NCT00860470). The process for registration began in 2007; however, the official release of the trial registration occurred after the study began due to numerous issues related to accurately documenting this large, community-based field trial on a site designed for patient-based, US clinical trials. It required substantial time and troubleshooting between the study investigators, the Johns Hopkins’ compliance officer, and the clinicaltrials.gov curators. A remnant of this process still remains with our major outcomes of infant mortality, preterm birth, and low birth weight being flagged as unrecognized conditions.

## Results

**Fig 1:**
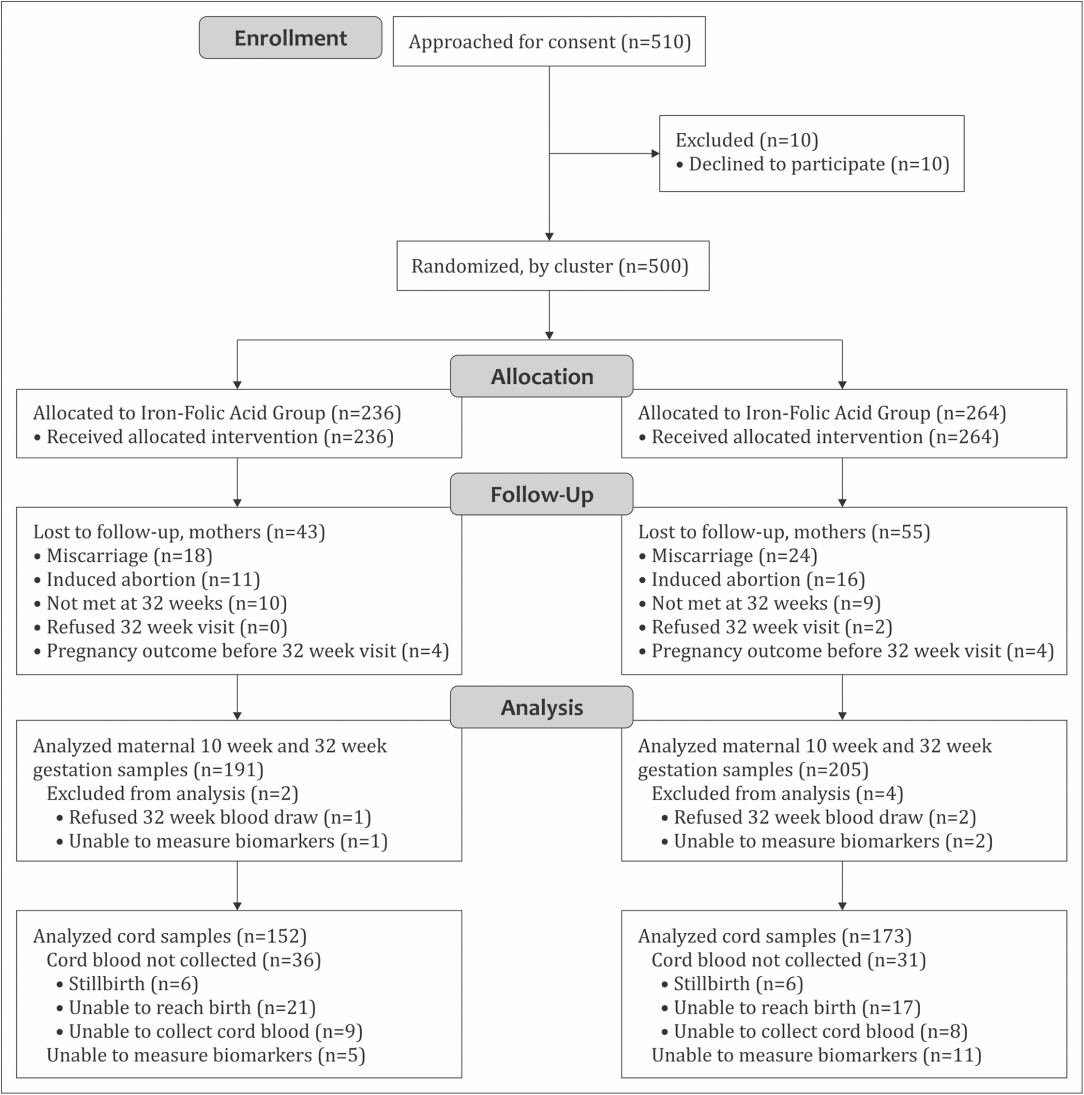
CONSORT flow diagram showing maternal and infant participation through the study. There were 16 clusters in the folic-acid group and 15 clusters in the multiple micronutrient group. Some mothers were not met at 32 weeks, but were met at birth for cord blood collection.

Fig 1 displays the flow of participants from enrollment to analysis. In all, 500 pregnant women were enrolled in the substudy, from whom 396 paired maternal blood samples and 325 cord blood samples were included in the analysis. Pregnancy loss (miscarriage, abortion, or stillbirth) occurred in 16% of enrolled women. Pregnant women at baseline were young (27% were <20 years old), parous (63%), short (22% with height <145 cm), and had few years of education (31% <4 years of school; Table 1). Sixteen percent were anemic (hemoglobin <110 g/L). Only 16% of households had electricity while 36% owned a mobile phone and 14% owned a television. Eight percent of infants were born preterm (<37 weeks), 63% were born small-for-gestational age, and a third were low birth weight (Table 1). Mean (SD) placental weight was 351 (74) g and birth weight was 2683 (414) g. These and other characteristics did not differ by supplementation groups (Table 1). Compliance was high–women consumed a median (IQR) of 95.5% (89.1, 98.4) of supplement tablets and compliance did not differ between supplementation groups (median 95.5% vs. 95.5% for MM vs. IFA, p = 0.39 by Kruskal-Wallis test).

**Table 1:** Characteristics of mothers and their offspring by supplementation allocation in rural Bangladesh, 2008–2009.

	Overall	Iron and folic acid	Multiple micronutrients	*P* value
	Mean ± SD or n (%)			
**Maternal (baseline)**	(n = 396)	(n = 191)	(n = 205)	
Age, y	23.4 ± 5.2	23.6 ± 5.4	23.1 ± 5.0	0.34
Height, cm	148.9 ± 5.4	149.3 ± 5.5	148.6 ± 5.2	0.21
BMI, kg/m^2^	19.5 ± 2.4	19.6 ± 2.6	19.4 ± 2.2	0.42
<18.5	152 (38.4)	64 (33.5)	88 (42.9)	0.07
18.5–24.9	236 (59.6)	121 (63.4)	115 (56.1)	
≥25.0	8 (2.0)	6 (3.1)	2 (1.0)	
MUAC, cm	23.7 ± 2.4	23.8 ± 2.4	23.6 ± 2.5	0.65
Hemoglobin, g/L	119 ± 10	119 ± 11	120 ± 10	0.37
Parity				0.63
0	148 (37.4)	71 (37.2)	77 (37.6)	
1	140 (35.4)	64 (33.5)	76 (37.1)	
≥2	108 (27.3)	56 (29.3)	52 (25.4)	
Completed years of school	5.6 ± 3.8	5.7 ± 4.0	5.5± 3.7	0.54
Has monetary earnings^1^	154 (38.9)	69 (36.1)	85 (41.5)	0.28
**Infant (birth)**	(n = 325)	(n = 152)	(n = 173)	
Birth weight (g)	2683 (229)	2723 (332)	2648 (316)	0.10
Placental weight (g)^2^	351.0 (4.1)	343.1 (5.4)	358.0 (6.0)	0.11
Gestational age (week)	39.1 (0.1)	39.3 (0.1)	39.1 (0.1)	0.37
Small for gestational age^3^	205 (63.1)	113 (65.3)	92 (60.5)	0.37
Low birth weight (<2.5 kg)	106 (33.6)	46 (30.3)	60 (34.7)	0.40
Preterm (<37 weeks)	25 (7.7)	13 (7.5)	12 (7.9)	0.90

^1^Represents economic activities that provide income since few women in this setting have formal paid jobs.

^2^One missing placental weight.

^3^Birth weight <10^th^ percentile of a gestational age and sex specific reference [23].

Maternal plasma PGH increased from a median (IQR) 2.6 (1.4, 7.6) ng/mL at 10 weeks gestation to 117.0 (74.8, 172.3) ng/mL at 32 weeks. Maternal hPL was 9.4 (7.5, 11.5) mg/L at 32 weeks. Cord plasma insulin was 2.8 (1.4, 5.4) μIU/mL, IGF-1 was 32.2 (17.8, 45.9) μg/L, and IGFBP-189.0 (55.3, 157.2) μg/L at birth. IGFBP-1 was negatively correlated with IGF-1 (r = -0.41) and insulin (r = -0.28) and IGF-1 was positively correlated with insulin (r = 0.20; all p < 0.01). PGH at 32 weeks was negatively correlated insulin (r = -0.14) PGH and hPL were not correlated with each other and hPL was not correlated with any of the fetal growth factors examined in cord blood (all p > 0.05).

There was no difference in mean concentrations of hPL at 32 weeks between those receiving MM vs. IFA when accounting for the cluster design of the trial (Table 2). We observed no differences in mean late pregnancy concentrations of PGH or the change in PGH from 10 to 32 weeks gestation in those receiving MM or IFA supplementation. As well, differences were not present for insulin, IGF-1, or IGFBP-1 concentrations by supplementation group (Table 2).

**Table 2:** Effect of prenatal multiple micronutrient supplementation compared to iron and folic acid on fetal growth factors in maternal and cord plasma, rural Bangladesh, 2009–2010^1^.

	Iron Folic Acid	Multiple Micronutrients	Mean difference^2^	95% CI
	mean± SD or median (IQR)			
**Maternal plasma** ^**3**^	**(n = 191)**	**(n = 205)**		
hPL^4^, mg/L	9.43 ± 2.76	10.00 ± 3.05	0.55	-0.17, 1.27
PGH, ng/mL	119.1 (74.2, 170.2)	112.3 (75.7, 175.0)	0.13	-20.8, 21.1
PGH Δ, ng/mL	114.7 (67.0, 166.2)	107.2 (71,5, 168.7)	0.19	-20.6, 20.9
**Cord plasma**	**(n = 152)**	**(n = 173)**		
Insulin, μIU/mL	1.01 (0.35, 1.72)	1.04 (0.50, 1.70)	8.3%	-14.4, 31.0%
IGF-1, μg/L	35.6 ± 26.0	32.5 ± 20.9	-3.24	-7.87, 1.40
IGFBP-1, μg/L	89.7 (50.6, 159.4)	89.0 (56.3, 148.4)	-0.003	-20.7, 20.7

^1^ hPL, human placental lactogen; PGH, placental growth hormone; IGF-1, insulin like growth factor; IGFBP-1, IGF-1 binding protein.

^2^ Linear regression models with generalized estimating equations (GEE) to account for cluster randomization. Results did not meaningfully differ when using ln transformed outcomes for PGH and IGFBP-1 so untransformed data were modeled. For insulin, mean difference is percent change.

^3^ hPL and PGH at 32 weeks gestation; PGH Δ is the concentration at 32 weeks–concentration at 10 weeks.

^4^ p = 0.05 for unadjusted difference.

**Fig 2:**
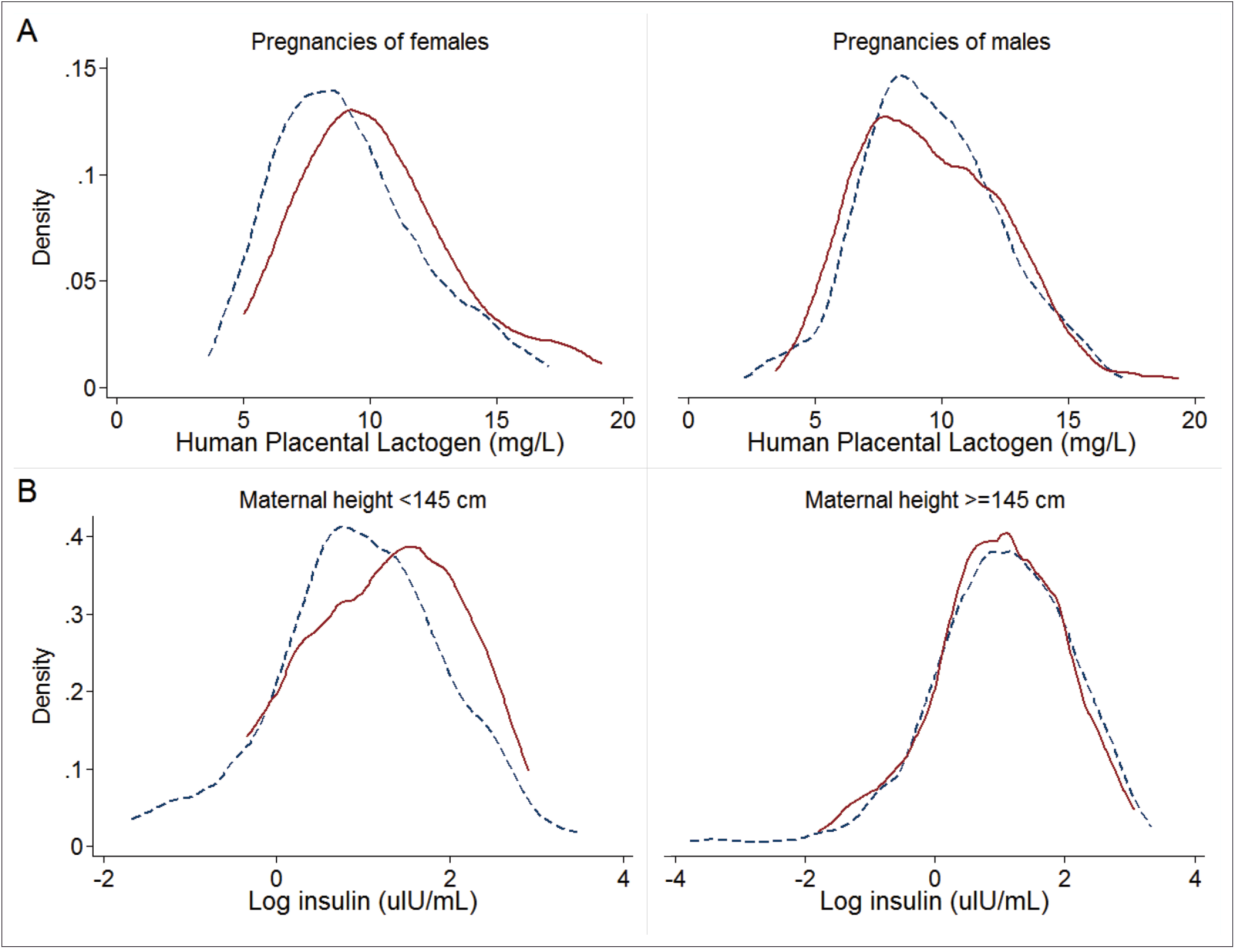
Effect of multiple micronutrient supplementation compared to iron and folic acid on a) human placental lactogen, by infant sex (p = 0.09 for interaction), and b) cord plasma insulin, by maternal stature (p = 0.04 for interaction). Solid line for multiple micronutrient group; dashed line for iron and folic acid group.

Examination of possible interacting variables revealed that among short women (<145 cm), insulin was 59% higher (95% CI: 3, 115) in cord blood of the newborns of women consuming multiple micronutrients compared to iron-folic acid (p = 0.039 for interaction; Table 3). There was no difference by supplementation group in cord insulin for women of normal height. Similarly, there was an interaction (p = 0.090) by fetal sex such that MM increased hPL in mothers carrying a female (but not male) fetus at 32 weeks gestation (Table 3). These interactions were reflected in distributions for insulin and hPL that were right-shifted among short women and mothers of female fetuses, respectively, who were taking the MM supplement compared to IFA (Fig 2). No other interactions between supplementation and infant sex, maternal BMI, age, and parity were statistically significant for any fetal growth factors.

**Table 3:** Effect of prenatal multiple micronutrient supplementation compared to iron and folic acid on plasma concentrations of growth factors by maternal and fetal characteristics in rural Bangladesh, 2009–2010^1^.

	Effect of Multiple Micronutrient vs. Iron Folic Acid Supplementation^2^									
	hPL (mg/L)		PGH (ng/mL)		Insulin (% change)		IGF-1 (μg/L)		IGFBP-1 (μg/L)	
	β	95% CI	β	95% CI	β	95% CI	β	95% CI	β	95% CI
**Maternal characteristics**										
Height										
< 145 cm (n = 91)	-0.12	-1.38, 1.15	-3.78	-48.7, 41.1	59.3^3^ ^,^ ^4^	3.3, 115	0.13	-10.5, 10.7	-11.5	-56.0, 33.1
≥145 cm (n = 305)	0.74	-0.03, 1.5	1.21	-22.6, 25.0	6.9	-34.3, 20.5	-4.38	-9.77, 1.01	3.27	-20.2, 26.7
BMI										
<18.5 kg/m^2^ (n = 152)	0.90	-0.12, 1.92	0.41	-34.7, 35.5	27.0	-15.6, 69.6	-2.72	-10.7, 5.29	22.9^4^	-10.7, 56.5
≥18.5 kg/m^2^ (n = 244)	0.20	-0.65, 1.06	1.87	-25.1, 28.8	0.7	-32.2, 30.8	-2.69	-8.82, 3.44	-14.8	-41.3, 11.7
Age										
<23 years (n = 198)	0.25	-0.67, 1.17	3.31	-27.1, 33.7	5.0	-30.4, 40.5	-1.11	-7.87, 5.65	11.4	-17.9, 40.7
≥23 years (n = 198)	0.84	-0.07, 1.75	-2.75	-33.2, 27.7	11.6	-24.2, 47.3	-5.47	-12.27, 1.34	-11.2	-40.5, 18.2
Parity										
0 (n = 148)	0.46	-0.57, 1.49	1.67	-33.6–37.0	27.4	-15.7, 71.0	-3.83	-11.92, 4.26	-1.3	-35.4,- 32.9
≥1 (n = 248)	0.60	-0.24, 1.44	-0.80	-27.8, 26.2	-3.4	-35.0, 28.2	-2.81	-8.86, 3.23	0.68	-25.4, 26.7
**Infant sex**										
Male (n = 212)	0.10^4^	-0.77, 0.97	-0.03	-29.5, 29.4	12.3	-21.8, 46.4	-3.47	-10.2, 3.21	-19.1^4^	-46.6, 8.46
Female (n = 178)	1.09^3^	0.16, 2.02	0.70	-31.6, 33.0	3.2	-34.0, 40.3	-2.84	-10.0, 4.36	21.5	-8.08, 51.1

^1^ hPL, human placental lactogen; PGH, placental growth hormone; IGF-1, insulin like growth factor; IGFBP-1, insulin like growth factor—1 binding protein. For insulin, IGF-1, and IGFBP-1, n = 70 & 255 for height groups; n = 129 & 196 for BMI groups; n = 162 &163 for age groups; n = 120 & 205 for parity groups; and n = 174 for males and n = 151 for females.

^2^ Mean difference in supplement groups from linear regression models with generalized estimating equations (GEE) to account for cluster randomization.

^3^ p<0.10 for interaction.

^4^ p<0.05.

Both hPL and PGH were positively associated with placental weight, and hPL alone was associated with birth weight z score (Table 4). Insulin and IGF-1 were positively associated with placental weight, birth weight z score, and MUAC, while IGFBP-1 was negatively associated with these outcomes (Table 4). In addition, IGF-1 was positively associated with length and head circumference. IGF-1 was negatively associated with gestational age at birth (β: -2.0, 95% CI: -3.5, -0.6 days per standard deviation increase in IGF-1). hPL, PGH, insulin, and IGFBP-1 were not associated with gestational age (p >0.05).

**Table 4:** Association between fetal growth hormones and birth size in rural Bangladesh, 2009–2010^1^.

	Placental weight (g)		Birth weight z score^2^		Length (cm)		Head Circumference (cm)		MUAC (cm)	
Per 1 standard deviation increase	β	95% CI	β	95% CI	β	95% CI	β	95% CI	β	95% CI
Maternal plasma (n = 396)										
hPL	21.6^4^	14.3, 28.9	0.11^3^	0.02, 0.21	0.13	-0.14, 0.40	0.120	-0.048, 0.289	0.05	-0.05, 0.15
PGH	12.4^4^	5.2, 19.6	0.01	-0.08, 0.11	0.13	-0.13, 0.38	0.040	-0.122, 0.202	-0.04	-0.14, 0.05
Cord plasma (n = 325)										
Insulin, μIU/mL	8.1^3^	0.2, 16.0	0.11^3^	0.01, 0.21	0.15	-0.08, 0.37	-0.028	-0.178, 0.123	0.12^3^	0.03, 0.21
IGF-1, μg/L	21.1^4^	13.5, 28.7	0.42^4^	0.33, 0.50	0.35^4^	0.12, 0.57	0.150^3^	<0.001, 0.299	0.26^4^	0.18, 0.35
IGFBP-1, μg/L	-11.2^4^	-18.9, -3.4	-0.19^4^	-0.28, -0.09	-0.08	-0.31, 0.14	-0.016	-0.164, 0.132	-0.16^4^	-0.25, -0.07

^1^ hPL, human placental lactogen; PGH, placental growth hormone; IGF-1, insulin like growth factor; IGFBP-1, IGF-1 binding protein; MUAC, mid-upper arm circumference. Linear regression models adjusted for maternal BMI, height, parity, age, education, and supplementation group (multiple micronutrient or iron and folic acid). Insulin, IGF-1, and IGFBP-1 were standardized to have mean = 0 and SD = 1. Exposures were standardized to have mean = 0 and SD = 1. β's represent mean increase in placental weight or other birth size outcome for each standard deviation increase in exposure.

^2^ Sex- and gestational age-specific z scores were calculated by a Canadian National Growth Reference [23].

^3^ p<0.05

^4^ p<0.01

Each standard deviation increase in IGF-1 was associated with a 30% decreased risk of SGA and a 43% decreased risk of LBW (**Table 5**). We also found a 12% increased risk of SGA and 16% increased risk of LBW for each standard deviation increase in IGFBP-1. There was no association between maternal hPL, maternal PGH, or cord insulin concentrations and risk of SGA or LBW, or any associations between the fetal growth factors assessed and risk of preterm birth (Table 5).

**Table 5:** Association between fetal growth hormones and risk of small for gestational age and preterm birth in rural Bangladesh, 2009–2010^1^.

	SGA		Low Birth Weight		Preterm	
	(<10^th^ percentile)		(<2.5 kg)		(<37 weeks)	
Per 1 standard deviation increase	Adjusted Risk Ratio	95% CI	Adjusted Risk Ratio	95% CI	Adjusted Risk Ratio	95% CI
Maternal plasma (n = 396)						
hPL^3^	0.93	0.86, 1.01	0.92	0.78, 1.07	1.04	0.75, 1.45
PGH	0.98	0.92, 1.05	1.02	0.86, 1.21	1.03	0.71, 1.48
PGH Δ	1.00	0.92, 1.09	0.95	0.82, 1.10	1.07	0.70, 1.63
Cord plasma (n = 325)						
Insulin, μIU/mL	0.94	0.82, 1.07	0.88	0.71, 1.09	1.08	0.78, 1.52
IGF-1, μg/L	0.70	0.63, 0.78	0.57	0.46, 0.70	1.01	0.77, 1.32
IGFBP-1, μg/L	1.12	1.05, 1.20	1.16	1.03, 1.30	0.86	0.58, 1.28

^1^ hPL, human placental lactogen

PGH, placental growth hormone; IGF-1, insulin like growth factor; IGFBP-1, insulin like growth factor—1 binding protein, Log-binomial regression models adjusted for maternal BMI, height, parity, age, education, and supplementation group (multiple micronutrient or iron and folic acid). All growth factors (exposure) were standardized to have mean = 0 and SD = 1.

## Discussion

In a rural area with low socioeconomic status and high rates of maternal and child malnutrition, we examined the impact of maternal MM supplementation compared to IFA on select fetal growth factors. There were no overall differences in maternal plasma levels of hPL or PGH or cord plasma levels of insulin, IGF-1, or IGFBP-1 between supplementation groups. However, MM supplementation resulted in higher cord insulin concentrations in women who were short and higher hPL concentrations in women carrying female fetuses. All growth factors were associated with placental weight and all but PGH were associated with birth weight z score; further, IGF-1 and IGFBP-1 were associated with risk of SGA and LBW. There was an inverse relationship between IGF-1 concentrations and length of gestation, but no other associations were observed between growth factors and gestational age.

In 1999, UNICEF, WHO, and the United Nations University called for research to evaluate the potential pregnancy benefits of a MM formulation containing 15 vitamins and minerals (called UNIMMAP) compared to IFA, the continued prenatal standard of care worldwide [20]. Evidence has been building from many randomized trials to support the benefit of MM over IFA given during pregnancy on reductions in low birth weight and SGA. [2, 24, 25]. In rural Bangladesh, our large trial of MM supplement vs. IFA found a significant improvement in gestational age and a consequent improvement in birth weight and other measures of birth size [3]. Both preterm birth and low birth weight were reduced, but there was no impact on SGA. In this proportionally small subgroup of the parent trial, those birth outcomes were not reflected.

Now research is needed to explain the modest but consistent improvement in birth weight and reduction in risk of SGA in response to the UNIMMAP or similar supplements in populations worldwide. Thus, we aimed to elucidate mechanisms of the MM supplementation impact on fetal growth, in the context of an undernourished setting, by examining the impact of micronutrient supplementation on hPL, PGH, insulin, IGF-1, and IGFBP-1, all which have been shown to impact birth size. We did not find an overall effect of MM on these growth factors and the power to detect such differences was low, at ≤50% for all outcomes. Aside from the sample size limitation, we postulate that the true lack of a difference between these factors by supplement group may be best explained by 1) the lack of an impact on fetal growth shown in the parent trial in which overall birth weight increments were attributed to the two days of longer time in utero [3]; and 2) the same increases in gestational age and birth weight were not observed in this substudy. Regardless of the MM effect on fetal growth and gestational age, we observed expected associations between measured growth factors and placental weight and birth weight.

To our knowledge, this is one of two studies that examined the link between maternal MM supplementation, cord blood growth factors, and infant outcomes. In Burkina Faso, where birth weight was 2.9 kg, cord blood insulin was 2.1 and 1.9 μIU/mL and IGF-1 was 29 and 31 ng/mL in the IFA and MM groups, respectively (not statistically different), showing no impact on UNIMMAP vs. IFA on cord hormones [19]. Yet there was a difference in effect by infant sex, where UNIMMAP increased IGF-1 in males but not females. Birth weight and IGF-1 concentrations were similar to those in our study while insulin was lower in Bangladesh. Also, the correlation of insulin & IGF-1 in our study (r = 0.20) was somewhat lower compared to the correlation reported in Burkina Faso study (r = 0.50). Not surprisingly, results differed for the two studies, with subgroup effects within different groups (i.e. males vs. females) and for different hormones (e.g. insulin vs. IGF-1). The parent trial in Burkina Faso found an impact of MM on birth weight, whereas our large trial in Bangladesh had an impact on length of gestation.

There are many potential and plausible modifiers of the MM effect on fetal growth factors that have been reported previously. Here we examined maternal early pregnancy (as a proxy for prepregnancy) BMI, height, age, and parity and infant sex. We found that maternal height modified the effect of MM on insulin such that only in the “short” women, presumably chronically malnourished, did the supplement have an impact over IFA. Our analysis by low BMI did not show such an interaction, suggesting that supplementation may be benefiting more the chronically, rather than acutely undernourished women. Of note, in these home births and even in the small number of clinic births, women did not have routine medical procedures during labor such IVs with glucose that could have impacted cord insulin concentrations.

Further, we found an infant sex interaction such that hPL was higher in mothers receiving MM that were carrying female fetuses. This finding is difficult to interpret yet contributes to the wide range of findings concerning sex differences in response to maternal nutritional status during pregnancy [26–29]. We speculate that girls may be more responsive to improved nutritional status, which could be reflected in higher hPL concentrations that in turn stimulate increased availability of maternal nutrients for the female fetus; whereas in males, a placenta already in “hyperdrive” may not show a detectable response. However research findings are quite varied in implications; differential sex response to improved nutritional status in under nourished mothers deserves attention. In the parent trial, MM supplementation significantly reduced infant mortality in girls (but not boys) [3], and although this may be unrelated to the hPL finding we present here, it also shows the benefit of MM supplementation was in girls.

Finally, we confirmed that these growth factors had the expected positive (hPL, PGH, insulin, and IGF-1) and negative (IGFBP-1) relationships with fetal and placental growth, even in a population with high rates of undernutrition. IGF-1 and IGFB-1 were related not just with birth size, but also fetal growth, as both factors were strongly associated with the outcome of SGA. Our unanticipated finding that IGF-1 was inversely related to gestational age is likely due to the fact that IGF-1 spikes in the third trimester and then declines until delivery [30].

Our study had a small sample size that was powered to detected differences in micronutrient status rather than growth factors. Testing MM compared to IFA presented several limitations including the lack of a placebo group and not being able to separate specific micronutrient effects. As well, we measured only five fetal and placental growth factors in a complex system of fetal growth regulation. Our blood samples were not collected according to ideal lab protocols due to extremely challenging field conditions. Yet, they were collected with the utmost care, were treated in a manner designed to maintain the integrity of the analytes of interest, and represent a rare opportunity to study these factors in the context of home births in a high nutritional-risk population.

Fetal growth occurs as a result of complex interactions throughout gestation between the maternal environment, including her nutrition and health, and endocrine factors of maternal, placental and fetal origin. In this study, growth factors were associated, as expected from other contexts, with size at birth and placental weight, demonstrating the utility of these biomarkers in settings with high rates of malnutrition. Daily, routine prenatal MM supplementation did not differentially impact, overall, fetal growth factors in maternal and cord blood compared to supplementation with IFA alone. This finding however was consistent with the findings of the parent trial–where MM affected length of gestation but not fetal growth. Differences in growth factors were observed in subgroups, including the nutritionally at-risk subgroup of stunted women, and these interactions should be explored in future human trials. Other potential pathways connecting micronutrient supplementation to fetal growth should also be explored.

## Supporting Information

S1 CONSORT Checklist CONSORT Checklist. (PDF)

S1 Protocol Trial Protocol. Parent Trial Protocol and Amendment.
(ZIP)
