# Supplementary material for: Effects of Prenatal Multiple Micronutrient Supplementation on Fetal Growth Factors: A Cluster-Randomized, Controlled Trial in Rural Bangladesh
Source: PLoS One. 2015 Oct 2;10(10):e0137269. doi: 10.1371/journal.pone.0137269 (PMC4591978; doi:10.1371/journal.pone.0137269)
Supplement: S1 Protocol — Parent Trial Protocol and Amendment. (ZIP) [file pone.0137269.s002.zip › Trial Protocol/Trial Protocol - supplemental information1a.pdf]

13 August 2007

Protocol

JiVitA-3: Antenatal Multiple Micronutrient Supplementation to Improve Infant Survival and Health in Bangladesh: A Double-Masked, Cluster-Randomized, Controlled Trial

<u>Principal Investigators:</u>
Dr. Keith P. West, Jr., Dr.P.H., R.D., Professor
Dr. Parul Christian, Dr.P.H., M.Sc., Associate Professor

<u>Co-Investigators:</u>
Dr. Rolf Klemm, Dr.P.H., Assistant Scientist
Dr. Alain Labrique, MHS, MSc., Assistant Professor
Dr. Mahbubur Rashid, M.B.B.S., M.Sc., Senior Medical Epidemiologist, JiVitA Project
Mr. Shamim Abu Ahmed, M.Sc., Senior Nutritionist, JiVitA Project
Dr. Kerry Schulze, Ph.D., M.Sc., Assistant Scientist
Dr. Joanne Katz, Sc.D., Professor
Dr. Alfred Sommer, M.D., M.H.S., Professor and Dean Emeritus

<u>Collaborating Institutions</u>
Institute of Nutrition at Mahidol University, Thailand
Institute of Nutrition and Food Science, Dhaka University, Bangladesh
DSM Ltd, Basel, Switzerland

<u>Current JiVitA Funding Agencies</u>
The Bill and Melinda Gates Foundation
US Agency for International Development

With additional support from:
Sight and Life Research Institute
The Government of the People's Republic of Banglades

1.0 Executive Summary....4
2.0 Background and Justification....6
  2.1 Individual Maternal Micronutrient Deficiencies and Their Prevention....6
  2.2 Antenatal Multiple Micronutrient Deficiencies....7
  2.3 Antenatal Multiple Micronutrient Supplementation....7
  2.4 Critical Questions to Address....10
3.0 The JiVitA Project: Overview....10
4.0 The JiVitA-3 Trial....12
  4.1 Overall Goal....12
  4.2 Specific Aims....12
  4.3 Study Design....13

4.4 Eligibility....14
4.5 Sample Size....14
4.6 Duration of Trial....15
4.7 Randomization....15
4.8 Supplements: Field Preparation and Distribution....16
4.8.1 Supplement Nutrient Composition....16
4.8.2 Supplement Production....17
4.8.3 Numbers of Supplements....17
4.8.4 Distribution to the Field....17
5.0 METHODS....18
5.1 Field Trial Procedures....18
5.1.1 Registration of Married Women of Reproductive Age....18
5.1.2 Pregnancy Surveillance and Ascertainment....19
5.1.3 Pregnancy Enrollment....19
5.1.4 Weekly Supplementation....19
5.1.5 First Trimester Assessment....20
5.1.6 Third Trimester Assessment....20
5.1.7 Birth Assessment....20
5.1.8 Maternal and Infant 1 Month Postpartum Assessment....20
5.1.9 Maternal and Infant 3 Month Postpartum Assessment:....21
5.1.10 Maternal and Infant 6 Month Postpartum Assessment:....21
5.1.11 Adverse Vital Outcome Assessment....21
5.1.12 Birth Defect Surveillance System....21
5.2 JiVitA-3 Substudies Procedures....22
5.2.1 Substudy Field Procedures....22
5.2.2 Storage of Substudy Biospecimens:....23
5.3 Procedures: Mapping and Geographic Information System (GIS)....24
5.4 Data Collection, Quality Control and Supervision....24
5.5 Data Management Procedures....25
5.6 Laboratory Analysis Procedures....26
6.0 DATA ANALYSIS....26
6.1 Comparison of Baseline Characteristics....26
6.2 Comparison of Losses-to-Follow-up....27
6.3 Comparison of Compliance Rates....27
6.4 Addressing the Primary Aim....27
6.5 Addressing Secondary Aims....28
7.0 ETHICAL ISSUES....29
7.1 Risks and Benefits....29
7.2 Compensation....30
7.3 Disclosure and Consent Processes....30
7.4. Confidentiality Assurances....31
7.5 Safety Monitoring....31
7.6 Institutional Review....31
8.0 COLLABORATIVE AGREEMENTS....32
9.0 TIMETABLE....32
10.0 REFERENCES....33

Table 1. Antenatal Multiple Micronutrient Supplementation Impacts on Birth Weight, Low Birth Weight and Preterm Delivery and Neonatal/Infant mortality..39
Table 2. Recommended Dietary Allowances and Recommended, Previously Used and Proposed JiVitA-3 Multiple Micronutrient Supplement Compositions....................................40
Table 3 Sample Size of Live Births and Pregnancies Required per Group (Ni)1....................41
Table 4. Multiple micronutrient tablet formulation and amounts...42
Table 5. Numbers of Supplements Required for JiVitA-3...44
Figure 1. JiVitA-3 Design..45
Figure 2. Proposed JiVitA Trial Timetables (Calendar Years)..46
Figure 3. JiVitA Staff Organization..47

1.0 Executive Summary

Poor, undernourished populations across South Asia can be expected to be deficient in essential vitamins and minerals (micronutrients). Women of reproductive age appear to be at high risk of single micronutrient deficiencies, in terms of prevalence and their potential health consequences, especially in relation to pregnancy and infant health. Examples include iron deficiency leading to anemia, low birth weight, and pregnancy complications, iodine deficiency predisposing offspring to mental retardation, periconceptional folate deficiency increasing risk of neural tube defects and vitamin A deficiency causing maternal night blindness and possibly increasing risk of maternal mortality. Recently, global interest has increased, led by UNICEF, to prevent multiple deficiencies by offering mothers a daily, multinutrient antenatal supplement. A 15-nutrient "UNIMMAP" supplement was formulated from an expert consensus in the late 1990s, which remains the formula of choice. While rational in concept, and nearly universally practiced in industrialized societies, data on the impact of antenatal multiple micronutrient supplement use on maternal and infant health outcomes are inconclusive and insufficient on which to base regional and global policies. Studies done to-date have largely focused on discerning effects on birth weight and gestational age, leaving their designs inadequate in size, duration and detail to assess other plausible outcomes that include multiple measures of infant size at birth, health, development and survival during infancy, and measures of maternal health, including obstetric and health complications through the late-pregnancy period. Missing, almost entirely, are data on which "benefit-risk" can be determined. At a presumed minimal cost of ~$0.05 per supplement when at-scale, daily multiple micronutrient supplementation will incur substantial costs in poor societies, increasing the urgency and importance to define the public health benefits. Given a likely widespread micronutrient deficiency problem and need for multiple micronutrient supplement use in South and Southeast Asia and Sub-Saharan Africa, practical assessment and intervention outcomes research is urgently needed in both regions to inform and guide policies.

As a critical step toward evaluating the public health impact of multiple micronutrient (MM) use by women in South Asia, JHU proposes to conduct a double-masked, randomized, community trial to evaluate the efficacy of providing mothers with a daily, antenatal-to-postnatal, multiple micronutrient supplementation to improve several major health outcomes, including fetal loss, growth, maturity at birth and risk of birth defects, infant morbidity and mortality, and maternal morbidity, including obstetric complications. The study will compare the effects of a micronutrient supplement containing an approximate Recommended Dietary Allowance (RDA) during pregnancy for 16 vitamins and minerals to a "standard of care" supplement containing iron + folic acid ("active control"). The trial will be conducted within an existing, densely populated, 650 sq km rural research area developed by the JiVitA Project over the past eight years with USAID and Gates support, located in northern Bangladesh. As the Project's 3rd large trial, "JiVitA-3" will plan to enroll ~36,000-pregnancies with an expected ~25,000 live births, with each of the two arms

enrolling ~18,000 pregnancies and ~12,500 live born infants. This sample size will be sufficient in size to discern with 95% confidence a difference in neonatal mortality of 15% or more.

Taking advantage of the organizational, procedural, personnel and community preparedness of the JiVitA Project that has been developed to carry out ongoing trials, JHU will plan to conduct a new census of married women of reproductive age and start enumeration, enrollment and supplementation of pregnant women for JiVitA-3 in August 2007. At an anticipated pregnancy recruitment rate of 18,000 per year among women registered at the outset of the trial, JiVitA-3 will take ~24 months to achieve its planned sample size of 36,000 pregnancies. Given about one year needed to follow the last of enrolled mothers and infants to six months of age, JiVitA-3 will complete field work by July 2010. Concurrent data entry, quality control, data management and analysis procedures of JiVitA will permit impact to be initially discerned within a month of completing field work. We expect the findings of JiVitA-3 to provide definitive evidence of impact of maternal MM supplementation on pregnancy-related health outcomes in a typical, rural South Asian population.

2.0 Background and Justification

Rural, undernourished South Asian populations are exposed to multiple micronutrient deficiencies from early childhood throughout adult life. Based on existing evidence, the most vulnerable period of maternal micronutrient deficiency and imbalance extends throughout pregnancy into the first postpartum year when breast feeding is most critical and infant health most precarious. It is widely recognized that adequate maternal nutrition is required to support increased metabolic needs related to pregnancy, maternal tissue growth including that of the placenta and body composition changes, fetal development and growth, and the stresses of parturition and lactation, and to maintain host defenses against coincident infections. Consequently, poor nutrition during this period of life may adversely affect maternal, fetal and infant health and survival (1).

2.1 Individual Maternal Micronutrient Deficiencies and Their Prevention

Health-promoting roles of micronutrients during critical maternal and infant stages of life have become increasingly visible from single-nutrient supplementation studies. For example, adequate maternal iodine intake helps to assure fetal development, prevents cretinism and, more broadly, lowers risk of later mental retardation (2, 3). Providing iodine directly to infants may also reduce their mortality (4). Periconceptional folate use can lower risk of neural tube defects (5, 6) and, in one study conducted by our group, has reduced preterm-born infant mortality (7). Iron supplementation during pregnancy can increase iron stores in infancy (8), lower risk of low birth weight (9) and may convey other more subtle developmental benefits to infants (10, 3). Infant (11-13) zinc supplementation may strengthen host defenses against diarrhea, other infections and mortality. Other micronutrients also play central roles in maternal and infant health, though data are presently more controversial. Maternal calcium supplementation may lower risk of neonatal mortality (14) and vitamins C and E taken antenatally may help attenuate pre-eclampsia (15,16), an underlying cause of fetal distress and maternal morbidity (17, 18). Low-dose supplemental vitamin A (or beta-carotene) can prevent maternal night blindness (19) and may reduce maternal mortality (20) in undernourished populations. Dosing infants with vitamin A shortly after birth may dramatically reduce their risk of mortality in the first several months of life (21, 22). And, injecting newborns with vitamin K is standard practice in many developed countries to reduce risk of bleeding disorders in early infancy (23).

Responses to single-nutrient supplements reveal the existence of individual maternal micronutrient deficiencies during pregnancy. However, it is likely that women in poor societies conceive in marginal-to-deficient micronutrient states, stemming from chronic dietary deficits that, in malnourished populations, extend to adolescence and childhood. To illustrate, in South Asia, vitamin A deficiency is common in early childhood (24), the school aged years (25, 26) and in early adulthood (27, 24). It is likely that chronic vitamin A deficiency predisposes mothers to night blindness in the latter half of pregnancy (28, 29) and persistent, associated health risks for years after pregnancy

(30). As another example, low iron intake during the reproductive years may fail to build iron reserves to support continuous demands of menstruation (31) and predispose women to iron deficiency anemia during pregnancy. Chronic and especially periconceptional inadequacy in maternal dietary folate may predispose offspring to neural tube defects considered to be a folate deficiency disorder (32).

It is important to note that health benefits of providing single nutrient supplements have been observed in separate trials in different populations, requiring their effects to be "cross-generalized" to build a case for widespread *multiple* micronutrient deficiency consequences and supplemental benefits.

2.2 Antenatal Multiple Micronutrient Deficiencies

There has been in recent years, increased momentum to prevent multiple, coexisting maternal deficiencies based on *limited* evidence that undernourished women indeed tend to be deficient in multiple micronutrients (33-36) and concern that co-existing deficiencies may pose considerable risks to maternal and infant health (37-41,3). Where examined, deficiency prevalence rates have ranged from a few percent (e.g., copper) to over 75% (e.g., zinc), with most being in the 15 to 50% range, even in the same populations. Internal variation might reflect variation in diets that disfavor some nutrients more than others and unequal effects of underlying diseases on nutritional status, as well as biochemical indicator insensitivity, incorrect cutoffs, and many other factors. The proposition, however, that a supplemental multiple micronutrient approach would be superior to single or double nutrient approaches is defensible given countless metabolic interactions and physiologic functions supported by micronutrients (42). This premise is central to the present protocol and the emerging global policies and programs this research project seeks to inform and guide.

2.3 Antenatal Multiple Micronutrient Supplementation

Micronutrient supplementation has been long held as an effective guard against dietary insufficiency and imbalance that can affect nutrition, performance and health. Currently, over half of American adults take dietary supplements, most of which are multiple micronutrient supplements (43). Supplement manufacturing in the United States alone is a $23 billion a year industry (43), although the effectiveness of multiple micronutrient supplement use is an issue of current debate (44). Virtually all pregnant women under physicians' care are prescribed antenatal supplements. Motivation for daily dietary supplement use hinges on periodic dietary surveys that often show average intakes for some nutrients that may be below the Recommended Dietary Allowance for certain life stage groups. Adequate absorption and utilization to support aspects of health specific to each supplied nutrient (and their interactions) are assumed. In general, however, experimental evidence is lacking in industrialized countries to support the use of multiple micronutrient supplements to improve maternal and infant health (including in the United States) because (a) supplement use is already nearly universal, (b) of an already richly fortified food supply, and (c) of a virtual absence of frank undernutrition to correct for discernable health reasons. Thus, in the developed world, antenatal multiple micronutrient supplementation is, basically, an insufficiently

proven intervention motivated by perceived need, theoretical bioefficacy, evidence of dietary lack in some groups, public aspirations to improve health, performance and longevity and commercial interest.

In the developing world, there is growing international advocacy to promote daily multiple, micronutrient supplement use to control maternal micronutrient deficiencies. While theoretically rational and aimed at resolving an apparent, widespread need, evidence supporting use of multiple micronutrient supplements is weak, with only five randomized controlled trials published to date on the efficacy of antenatal multiple micronutrient supplement use in non-HIV-affected women, and one in HIV+ women (**Table 1**). All have focused on altering risks of low birth weight and, at times, also of prematurity. In Mexico, where women were randomized to receive either iron (60 mg) or a multiple-micronutrient supplement containing iron, there was no observed difference in birth weight between groups (45). A study in Zimbabwe which included both HIV-1 infected and uninfected pregnant women, found a small overall reduction in low birth weight, with the effect being smaller among the non-HIV-1 infected women (shown in Table 1) (46). Neither reduction was statistically significant. In this study, all women also received 60 mg of iron and 400 µg of folic acid as per the national policy. A trial in Guinea-Bissau showed no significant effect on birth size following single-RDA multiple-micronutrient supplementation compared to daily iron-folic acid supplement use (47). However, a multiple-micronutrient supplement that provided twice the RDA for all nutrients significantly increased birth weight by ~90 g. Its use was also was associated with a reduction in miscarriage, but the study was not designed to evaluate this outcome and no explanation was provided for this effect. All groups showed comparable rates of perinatal or neonatal mortality across groups, although the study was insufficient in size to show differences in mortality.

Two trials in Nepal deserve special mention because of their relevance to South Asia, and similarity in design, type of population, and analytic approach to outcomes assessed. The first, conducted in ~5000 pregnant women in southern, low-lying plains District of Sarlahi, tested the use of four additive combinations of micronutrients from early pregnancy through six weeks postpartum, compared to a control supplement (48, 49). The supplements contained (a) folic acid alone, (b) folic acid+iron, (c) folic acid+iron+zinc, and (d) a multiple-micronutrient also containing the above 3 plus 11 other micronutrients. All supplements contained an RDA of vitamin A. Folic acid supplementation alone had no impact on birth weight, but lowered the risk of infant mortality in the first three months of life by over 20 percent. Although not statistically significant, the entire effect was confined to preterm born infants, among whom the mortality reduction was over 60 percent and highly statistically significant. Adding iron to folic acid improved maternal hemoglobin concentration, increased mean birth weight, and reduced the prevalence of low birth weight, but only slightly improved infant survival beyond that achieved with folic acid alone. Adding zinc blunted the benefits of iron without measurably affecting infant survival. Intake of the 14-nutrient supplement exerted the greatest increase in mean birth weight (by 64 g), but surprisingly failed to benefit infant survival, compared to the control group.

The second trial was conducted in the nearby plains district of Dhanusa. This study reported a significant increase in birth weight (77 g, 95% CI: 24-130) among infants born to mothers receiving the UNIMMAP supplement compared to iron-folic acid

(50), but without any improvement in infant survival, similar to the trial in Nepal. Data from both Nepal trials were recently pooled (51) to compare the impact of maternal multiple-micronutrient versus iron-folic acid use (which was one of the intervention arms in the Sarlahi study) to reveal significant increases of 36% and 52% in perinatal and neonatal mortality, respectively. Recently, a Cochrane meta-analysis of multiple micronutrient supplementation, which includes the above mentioned trials, plus another unpublished study from Pakistan showed reductions in low birth weight of about 17% (95% CI 9%-24%) when compared to placebo or two or less micronutrients. However, no statistically significant differences were observed when compared with iron-folic acid supplementation (52).

Thus, research so far has not yet provided a clear platform of evidence to support the widespread antenatal use of multiple-micronutrient supplements. Birth size has been improved in some studies and anemia reduced, but two studies in one population (southern Nepal) observed increased birth weight and risk of neonatal mortality. The lack of strength in evidence of population benefit was highlighted in a micronutrient evidence policy summit during the Innocenti Meeting in Florence, Italy in 2005 (53). The above trials leave the international nutrition community with the following lessons and interpretations, so far, about the effectiveness and safety of maternal multiple micronutrient (MM) supplements:

- Studies have not been able to systematically show an impact on birth weight or preterm birth; in those where an effect has been observed, the magnitude of increase in birth weight has been small.
- Insufficient research has been done to reliably discern effects of antenatal MM supplementation for crafting policies in high-risk regions of the world.
- Studies have generally failed to assess the range of plausible public health outcomes that may improve with MM supplementation; limitations include small sample sizes (i.e. with low power to detect, for example, effects on mortality), insufficient duration of follow-up beyond birth, lack of inclusion of indicators reflecting a larger number of plausible outcomes (e.g., survival, growth, development, maternal complications). Thus, it is presently not known whether maternal MM supplementation can, for example, reduce infant mortality.
- Investigators should be prepared to observe and explain different effects of MM supplements on the same outcome (e.g., birth weight) in populations of varying nutritional status and health risk (e.g., SS Africa vs. S Asia vs. Latin America).
- Antenatal MM effects may also vary qualitatively for different outcomes in the same population (e.g., improved survival may not follow increased birth weight).

In summary, presently the evidence of benefit to mother and infant from maternal multiple micronutrient supplementation in the developing world is equivocal, leaving in question the wisdom of advocating global use of this intervention until there is adequate data on its range of benefits and safety. Additional, adequately designed and sized trials are essential to inform and guide this major intervention strategy in South Asia, Sub-Saharan Africa and Latin America.

2.4 Critical Questions to Address

A research strategy seeking to provide evidence of benefit and safety of antenatal micronutrient supplement use should assess a wide range of plausible effects in each major region where fetal loss, prematurity and infant and maternal mortality are high and coexist with maternal and infant malnutrition. Specifically, trial research should, to the extent possible, determine the efficacy of supplementation in:

A) Improving infant survival, fetal survival and intrauterine growth, including
- Reducing neonatal mortality, overall and by gestational age and size at birth
- Reducing fetal loss (i.e., stillbirth and miscarriage)
- Reducing low birth weight and small size-for-gestational-age at birth
- Normalizing gestational age at birth and reducing risk of preterm birth

B) Influencing infant micronutrient status, growth and development, including
- Improving linear, ponderal and body compositional growth through six months of age
- Improving newborn and infant micronutrient status and stores for sentinel nutrients
- Early achievement of motor and behavioral milestones by six months of age

C) Improving maternal health, including
- Reducing prevalence and severity of pregnancy-related and infectious morbidity and mortality during pregnancy
- Reducing anemia in the third trimester and postpartum period
- Reducing risks and severity of major obstetric complications (i.e. pre-eclampsia and eclampsia, sepsis, hemorrhage, prolonged and obstructed labor and shock)
- Improving nutritional status, including micronutrient status and body composition
- Improving other biomarkers of nutritional, antioxidant and chronic disease status

Further, trials should be designed and conducted from the outset in a way that could allow randomized, intervened cohorts of offspring to be followed in the future, given increasing evidence of long-term, latent effects of developmental nutritional exposures on adult health, disease and survival.

The Johns Hopkins Micronutrient Research Team, in collaboration with its national and international partners, proposes to assess the efficacy and safety of maternal multiple micronutrient supplementation, from early pregnancy through postpartum, on a range of public health outcomes in a rural South Asian setting - the JiVitA research study area located in northern Bangladesh.

3.0 The JiVitA Project: Overview

The USAID-Gates-supported JiVitA Project is located in Gaibandha and Southern Rangpur Districts of northwestern Bangladesh. The study area is a vast, rural, agrarian site of nearly 650 sq km with a population of ~650,000 people (a population density that exceeds 1000 per sq km). In these and numerous health, agricultural, geographic, demographic and infrastructural features, the JiVitA study area broadly represents poorer, though not the poorest, rural areas of Bangladesh and, more broadly, the greater Gangetic floodplain communities of South Asia. Advantages of having JiVitA carry out the trial proposed herein include

- National recognition as an official research component of the National Integrated Population and Health Program (NIPHP) under the Ministry of Health and Family Welfare of the Government of Bangladesh, with tax exemption status through a bilateral agreement with the Government of the United States,
- 850 trained staff with excellent field, clinical, laboratory and data-handling research skills from carrying out large trials among pregnant women (JiVitA-1; N=67,000) and newborn infants (JiVitA-2; N=25,000),
- An extensive network of diverse, collaborating institutions (NGOs, Universities, Research Centers) in and outside of Bangladesh,
- State-of-the-art technology, including GIS and high-capacity data management, field communications networks, sophisticated biospecimen sampling, storage, processing and overseas shipment ,
- Standardized and integrated administrative, financial, logistical, personnel, procurement and inventory policies and procedures,
- Strong history of community trust earned while building integrating project activities into the area since 1999,
- An established study population divided into nearly 600 project-defined, household-level GIS-mapped, community clusters (called “sectors”) forming blocks that are designed for randomization and as supervised field work units.

As part of ongoing trial activities, JiVitA maintains a 5-weekly pregnancy surveillance system among ~130,000 women of reproductive age, and is capable of ascertaining, supplementing and tracking up to ~18,000 pregnancies per year (1500 per month). The project has a birth surveillance system capable of reaching ~80% of home-born infants within the first day of life for intervention, assessment and follow-up. JiVitA maintains a lay-worker trained birth defect surveillance system, backed up by physician examination and digital photography that has developed the most extensive rural birth defect database in South Asia. Among the project’s 850 trained staff, 90% are local female residents. Professional Bangladeshi staff include half-dozen MBBS-level research physicians (supervising substudies, assessing birth defects and interviewing families about causes of maternal death) including three with advanced degrees, a masters’ level senior nutritionist, social scientist, computer scientist and administrator, backed by trained field, logistics and administrative staff.

Operations are managed through a project headquarters in the provincial city of Rangpur, a field station in the district town of Gaibandha and 70 field offices located throughout the rural study area. All field offices are equipped, staffed and managed to follow and implement standardized operational procedures. Daily operations of the

Project are managed by a Senior Management Team, Field Management Team, 14 Area Coordinators and 56 Team Leaders. Standardized data collection, personnel, inventory, accounts and logistics policies provide the operational framework of the JiVitA Project. Staff is paid monthly through a collaborative and secure network of 14 village-based banks, following exacting standards of accounting. Johns Hopkins typically maintains 2-3 resident faculty or technical staff positions to oversee the scientific and operational aspects of the Project. As the JiVitA offices have access to broadband technology, Baltimore and Bangladesh-based JiVitA teams maintain routine daily communication on a number of levels through email and instant messaging.

Presently, the JiVitA-1 trial is scheduled to complete its recruitment of pregnant women by January 2007 and all 3-month postpartum follow-up assessments by September 2007. JiVitA-2, which enrolls and doses newborns of JiVitA-1 mothers, will cease obtaining consent from 3rd trimester mothers by April, complete its program of dosing newborns by the end of June, and also complete its infant follow-up phase by the Fall of 2007. These time-points are critical because, at the close-out of recruitment into JiVitA-1, the JiVitA Project is prepared and capable of reassembling, recensusing, and carrying out an updated assessment of, and re-consenting and enrolling ~130,000 resident women of reproductive age into a third, JiVitA-3 multiple micronutrient trial. We anticipate being ready to begin field work and start enrolling the first of the planned 36,000 newly pregnant women within 4 months of closing out the enrollment of women into JiVitA-1.

4.0 The JiVitA-3 Trial

4.1 Overall Goal

The overall goal of JiVitA-3 will be to evaluate the impact of daily, maternal multiple micronutrient supplementation on infant survival and health, using a supplement containing 16 essential vitamins and minerals at dosages corresponding to recommended dietary allowances (RDA) during pregnancy and lactation (54, 55) and the currently recommended formulation by UNICEF and WHO (56). Health outcomes will be compared to an “active” control group of women who will receive a daily antenatal “standard of care” iron + folic acid supplement at the same dosages as provided in the multiple micronutrient supplement.

4.2 Specific Aims

Primary Aim

1. Infant Survival: Determine the efficacy of a standard MM supplement given to women daily during pregnancy through 12 weeks postpartum in lowering neonatal and infant mortality through six months of age by $\geq$15% compared to the mortality of infants whose mothers receive daily iron + folic acid.

The sample size required in each proposed intervention arm and will be 17,950 pregnancies, which are expected to yield 12,565 live births (see below).

Secondary Aims

2. <u>Fetal and Newborn Outcomes</u>: Assess the efficacy of the MM intervention in reducing the
    a) Stillbirth rate by 23% or more, from an expected 30 to 24 or fewer stillbirths per 1000 births (i.e. live + still births);
    b) Rate of preterm birth (live birth delivered $\leq$ 37 weeks' gestation) by 10% or more, from an expected 20% to 18% or fewer of all live births;
    c) Prevalence of low birth weight (<2500 g) by 5% or more, from an expected 40% to 38% or fewer of all live births; and
    d) Neonatal morbidity related to sepsis, birth asphyxia, hypothermia and diarrhea.

3. <u>Other Infant Outcomes (to 6 months of age)</u>: Assess the efficacy of the MM supplement intervention in improving
    a) Linear and ponderal growth, including its ability to protect lean body mass, that could lead to reduced prevalences of stunting, wasting and underweight status in infancy;
    b) Infant morbidity, including diarrhea and acute lower respiratory infection, in the first 3 months of life;
    c) Micronutrient intake, represented by measured breast milk micronutrient concentrations;
    d) Micronutrient status, and reducing prevalences of multiple deficiencies;

4. <u>Maternal Outcomes</u>: Assess the efficacy of either MM supplement intervention in influencing among mothers the
    a) Prevalence of infectious morbidity, based on history, testing and signs, during pregnancy and in the 1st six months postpartum;
    b) Rates of potentially fatal ("near miss") obstetric complications;
    c) Prevalence of anemia and micronutrient deficiencies in the 3rd trimester and at 3 months postpartum (assessed in subsamples of blood and breast milk); and
    d) Lean body mass depletion and changes in adiposity, assessed by body composition measurements, during and following pregnancy.

5. <u>Longer Latency Outcomes:</u> While not a formal part of the currently proposed trial, JiVitA-3 will be designed and conducted in ways, that include the establishment of a long-term archive of biological specimens (maternal plasma, buffy coat, breast milk and urine) that will facilitate future investigations of longer-term health, functional and survival outcomes that could be attributed to enriched developmental fetal and infant exposure to micronutrients achieved by maternal MM supplementation.

4.3 Study Design

JiVitA-3 will be a two-arm, cluster-randomized, double-masked, controlled trial to evaluate the efficacy of giving pregnant and postpartum women a daily multiple

micronutrient supplement in reducing early infant mortality. The units (clusters) of randomization will be 596 previously defined, mapped and addressed “sectors”, each with ~250 married women of eligible age. Prior to the outset of the trial, half of the sectors (~298) will be randomly assigned for consenting pregnant women to receive one of two coded supplements through 12 weeks postpartum, or until censor due to out-migration, refusal, death or the end of the trial. The design of JiVitA-3 trial is presented in **Figure 1**.

Each coded regimen, described in Table 2, will consist of either

- An antenatal and postnatal iron-folic acid supplement (standard of care) containing 27 mg and 600 □g, respectively; or
- An antenatal and postnatal MM supplement (Table 2).

4.4 Eligibility

Women under 45 years of age who are married and living with their husbands as residents of the JiVitA study area at the time of an initial population enumeration, and those who enter the cohort as newlyweds, will be eligible for pregnancy surveillance and, when identified as pregnant, enrolled, supplemented and followed in the trial. All eligible married women will be approached for consent in conjunction with husbands or parents as needed, but will be considered as autonomous adults. Women will be eligible to contribute more than one pregnancy to the trial, for which they will receive the same sector-assigned supplement. From previous data in JiVitA-1, the mean interval between two live births is 36 months, whereas that following a miscarriage is shorter, around 18 months.

4.5 Sample Size

The number of live born infants needed to be enrolled into JiVitA-3 is that required to discern whether or not maternal MM supplementation can reduce infant mortality by at least 15% in the first six months of life. Sample size estimation requires that we consider a plausible range of effect sizes (i.e., percents reduction in 6-month mortality attributed to intervention), a design effect (i.e., potential loss in precision in effect estimation due to randomizing clusters vs. individuals), possible losses to follow-up (using estimates based on existing JiVitA trials), acceptable probabilities for Type I (α=0.05) and Type II (β=0.20) errors, and the rate of infant mortality expected in the control group.

Table 3 provides a matrix of sample sizes required to detect reductions in six-month infant mortality across a plausible range of mortality rates. A 15% reduction in infant mortality represents the *a priori* minimum detectable difference of public health importance for this trial. The calculated number of live born infants required to detect this difference is n_i=12,565 per group, assuming a control group 6-month infant mortality rate of 60 deaths per 1000 live births, a design effect of 1.15, an expected 5% loss to

follow-up, and accepted Types I and II error probabilities of 0.05 and 0.20, respectively. A control group mortality rate of 60/1000 is conservative, and lower than presently observed in the study area (i.e., 76/1000) but can be expected given that newborns could be receiving vitamin A shortly after birth, should the current JiVitA-2 trial show this intervention to reduce mortality risk. This sample size will also permit minimum reductions in infant mortality of 17% and 18% to be detected should the control rate be lower, such as 55 or 50 infant deaths per 1000 live births, respectively.

Thus, the total number of live births required will be 25,130 summed across the two groups. Given an expected 30% pregnancy loss rate (due to spontaneous and induced miscarriage and stillbirth), based on extant data in this population, JiVitA-3 will require 17,950 pregnancies per group, or a total of 35,900 enrolled pregnancies (ie, ~36,000 pregnancies). Larger differences in infant mortality, especially 20% or higher, would substantially reduce the final sample size. A higher than expected infant mortality rate in the control group would also reduce the numbers of pregnancies and live born infants required to be enrolled to meet our primary aim of infant mortality reduction Either condition would shorten the duration of the trial (see below).

4.6 Duration of Trial

Based on existing census data and experience, we expect JiVitA-3 to recruit and begin supplementing ~18,000 pregnant women per year. Thus, pregnancy enrollment can be expected to take two years (24 months) to reach the planned sample size of 35,900 pregnancies. Assuming pregnancy enrollment and supplementation begins by September 2007, the full sample size should be attained by the end of August 2009. Field work (supplementation, interviews and vital status follow-up through 6 months postpartum) among the last of enrolled pregnant women and their infants will be completed by August 2010, with initial reports of impact available shortly thereafter. Thus, JiVitA-3 is projected to be in the field for ~3 years (**Figure 2**).

The planned duration is guided by the population size, assumed rates of pregnancy ascertainment and infant mortality in the control group, and by the aim to discern a 15% reduction. A higher control infant mortality rate, for example of 65 (vs. 60 as planned) deaths per 1000 live births would require nearly 1500 fewer pregnancies (i.e., 16,490 vs. 17,950) per group (Table 3). A 20% (vs. 15%) reduction in 6-month infant mortality would nearly halve the required sample size (i.e., 9,915 vs. 17,950 pregnancies), and enrollment period from 1.5 years (i.e., 18 months). These trends and differences will be periodically reviewed by an independent data and safety monitoring board throughout the trial. The DSMB would be expected to issue recommendations to the investigative team concerning the strength of emerging findings that could alter sample size requirements and duration of the trial, conditioned on findings up to a point in time.

4.7 Randomization

Randomization provides reasonable assurance that groups are comparable in their health risks in the absence of an intervention. Operational efficiencies are gained

when units of randomization are community clusters of households. Statistical efficiency is gained when these units are similar in size. There are no administrative subdivisions in rural Bangladesh that meet the cluster size requirements for JiVitA-3. Therefore, we plan to use previously divided study population units of 596 clusters, called “sectors”, of comparable numbers of households. The sector also serves as a unit of work for each locally resident female field worker (field distributor). Sector maps and statistics are regularly updated into the JiVitA GIS system.

For JiVitA-3, sectors residing on an existing JiVitA master list, each with a 3-digit numeric identifier will be listed in contiguous, geographic order. Using a computer-programmed routine the numbers 1 and 2 will be randomly generated within randomly permutated blocks of 6 and 8 numbers, to which the 596 existing sectors, contiguously listed to balance for geographic variation and related factors, will be assigned, with each number representing one of the two supplement formulations, coded A and B. Codes identifiying the supplement content will be maintained in sealed envelopes, with one set under lock and key at the Beximco production facility and another under secure, locked conditions at the Johns Hopkins Bloomberg School of Public Health. Confidential sector lists will be produced for each of the two codes, which will be confidentially maintained by a management-level JiVitA staff member in charge of supplement logistics (but unrelated to field work). The lists will serve as the basis for issuing sector-coded supplements to the field for use by enrolled women.

4.8 Supplements: Field Preparation and Distribution

4.8.1 Supplement Nutrient Composition

It is the aim of JiVitA-3 to evaluate a MM supplement that would most likely be used in global programs: one that provides a range of vitamins and minerals at their recommended dietary allowance level (an RDA). Practically, such a supplement should also be similar to any formulation advocated by UNICEF and, to the degree possible, be in compliance with existing dietary guidelines. The composition of the proposed MM supplement for JiVitA-3, as described in the final column of Table 3, reflects the current RDA for pregnant women for 15 essential vitamins and minerals, as specified by the Institute of Medicine (IOM) in the United States. The nutrient composition and dosages are close, but for some nutrients not identical, to those in the UNICEF UNIMMAP supplement that was derived by expert panel discussions in 1998, and has since been updated and aligned with the IOM recommended allowances for use in emergency settings (57). Thus, most differences between the proposed JiVitA-3 supplement and the original UNICEF formulation are ones that reflect the IOM dietary recommendations that include adding 50% more folic acid and iodine, 50% less copper and other slight differences for a few other nutrients. Consistent with the UNICEF formulation, there is no vitamin K in the proposed JiVitA-3 supplement. The proposed JiVitA-3 supplement also differs slightly from the NNIPS-3 supplement (used in Nepal in 1999-2001), that predated the UNIMMAP mixture, in that it includes two additional nutrients not used in Nepal (iodine and selenium). We propose that the standard-of-care, the active control supplement for JiVitA-3, contain 27 mg of iron (as fumarate) and 600 µg of folic acid (listed in the footnote of Table 3). Johns Hopkins has been collaborating with DSM and

Task Force Sight and Life (Basel, Switzerland) to formulate the new JiVitA-3 supplement (**Table 4**), that will use accepted, industry-standard ingredients.

4.8.2 Supplement Production

As of November 2006, working with the South Asian Regional Office of DSM and Sight and Life, the Johns Hopkins team has secured an agreement from and has been working closely with Beximco Pharmaceuticals, Ltd, the second largest pharmaceutical manufacturer in Bangladesh, to produce, bottle and label all JiVitA-3 supplements according to full specifications in-country. Beximco was approached after independent tests by DSM of multiple micronutrient supplements obtained off the shelf in Dhaka showed their formulation to be as claimed on the label for all nutrients. As partners in the trial, DSM has agreed to provide the nutrient premix to Beximco without charge, and Beximco, under the terms of a Memorandum of Agreement signed between JiVitA and Beximco in July 2007, has agreed to manufacture, bottle and label all supplements without charge to the JiVitA Project at its state-of-art, new production facility north of Dhaka. Premix will be produced on two occasions (outset and mid-study) at the DSM Singapore production facility and shipped to Bangladesh. The JiVitA in-country team will work with Beximco to clear the premix through customs for transport to the Beximco plant. Manufacturing the supplements in Bangladesh will result in substantial savings and efficiencies in terms of production, labeling, storage, potency maintenance and delivery of supplements to the project site, which lies ~350 km north of the Beximco plant. An initial test batch of supplements from an initial premix produced in Singapore was produced in December 2006 for accelerated stability testing between January and March 2007. The supplements underwent independent analysis for content at a US-based laboratory (Medallion Laboratories) which found nutrient concentrations to be acceptably close to the intended label claim for most although not all nutrients. With further analyses by DSM, the reasons for overages and underages in the initial test batch have been worked out to allow production of the first batch to be planned for late August 2007 and delivered to the JiVitA Project in September 2007.

4.8.3 Numbers of Supplements

The estimated numbers of supplements need for JiVitA-3 are provided in **Table 5** for the projected sample size required to reduce the 6-month infant mortality rate of 60 per 1000 by 15%, and also for other scenarios where infant mortality or the percent reduction may be higher. According to our present plan, JiVitA-3 will require a total of 16 million supplements (8 m each of multiple micronutrient and iron + folic acid) over the entire three years of anticipated field activities. This figure includes a 20% overage for losses and sample size adjustments, if required. We are planning for study supplements to be produced, bottled and labeled in two production batches of 8 million each (4 million per supplement group), with the first due to go on stream in August 2007 and in second around November 2008.

4.8.4 Distribution to the Field

Supplements are to be produced in Bangladesh, bottled, descriptively labeled and coded as “A” or “B”” according to a key that links the labeled codes to their nutrient content. The key linking the labeled codes on bottles to actual nutrient content will be

kept in wax-sealed envelopes under lock and key at the supplement manufacturer in Bangladesh and at the Johns Hopkins Bloomberg School of Public health in Baltimore, MD. All supplement bottles will be plastic, opaque, appropriately labeled for JiVitA research purposes, and marked with a batch number and expiry date. Filled and labeled 250-count supplement bottles will be transported by road to the project area, approximately 350 km north of Dhaka, where they will be stored under light protected, temperature and humidity-controlled conditions. Following JiVitA convention, the two factory-imprinted supplement codes on bottles, reflecting the two treatment groups, will be confidentially labeled-over by a logistics staff member to 3-digit identifiers representing each of the 596 sectors that will have been pre-randomized for women to receive one of the two study codes. Thus, in the field, the trial will appear to have 596, rather than two supplement groups to "double mask" JiVitA staff, investigators, and community and to diffuse potential, general interest in two differently coded batches of supplements. The 250-ct bottles will be distributed to "Field Distributors" by Team Leaders (one of 56 field supervisors in charge of 56 local teams of distributors), along with personal-sized, empty, sector and JiVitA-identifier labeled plastic bottles. Supplement bottles will be distributed on a regular basis to each of 56 team offices in the study area that will then be disbursed to our 596 local field distributors according to their sector code. As pregnant women are recruited for the trial, field distributors will place 14 tablets into a woman's personal, study-number (bar-coded) bottle which will be monitored and refilled each week thereafter through 12 weeks postpartum

5.0 METHODS

Many field and data management methods proposed for JiVitA-3 are modifications of procedures that have been established, standardized and documented in existing manuals of operation for the conduct of the JiVitA-1 and JiVitA-2 trials (58). We will implement a set of procedures that will involving registering and periodically visiting women of reproductive age (i.e., ~13 to 45 years) in the 596 study sectors for occurrence pregnancy, and then enrolling, supplementing and following pregnancies for vital and morbidity outcomes.

5.1 Field Trial Procedures

5.1.1 Registration of Married Women of Reproductive Age

At the outset of the trial, we will conduct an initial census, registration and pregnancy ascertainment of an estimated 130,000 married women of reproductive age who are residing with their husbands. At this time, we plan to collect limited data on their SES, parental vital status and arm circumference size (reflecting nutritional status). Pregnancy will be ascertained by asking women if they are pregnant and by eliciting a history of menstruation in the previous month, following procedures we have developed previously in Nepal and Bangladesh. Since the trial seeks to enroll and supplement women as early as possible in pregnancy, women who respond that they know they are pregnant (thus likely to be relatively advanced in pregnancy), or who are breast feeding an infant <12 months of age (thus, not likely to be menstruating due to lactational

amenorrhea) will be temporarily excluded from entry into the pregnancy surveillance system until they either report resuming menstruation or enter their second year postpartum, whichever occurs first. At the time of the initial census, registered, women who have not menstruated in the past month but also do not know if they are pregnant (thus, possible early gestation pregnancies) will be offered a urine test and, if positive, asked to enroll into the trial (see below). Otherwise, registered non-pregnant women will be asked to participate in a pregnancy surveillance system.

5.1.2 Pregnancy Surveillance and Ascertainment

After the initial registration round, 115-120,000 consenting women (who were not initially pregnant) will be visited every five weeks at home by our field team of 596 local female workers (~1 per sector) over the next 2 to 3 years. During these home visits, usually lasting a few minutes, women will be asked about menstruation in the previous month. Those reporting to be amenstrual will be offered a urine test. Test-positive women will be asked to enroll into the trial (see below). Women who report to have entered menopause, who have become divorced or whose husbands have died will be excluded from subsequent pregnancy surveillance rounds. Otherwise non-pregnant women will continue to be visited at home in the same way until the sample size of ~36,000 pregnancies is attained. During surveillance, newly married women will be asked to be registered into the surveillance system, tested for pregnancy if not menstruating, and offered an opportunity to enroll into the trial if identified as pregnant. We expect to register ~10,000 newly pregnant women over the course of the ~2 year surveillance period.

5.1.3 Pregnancy Enrollment

Within a week of the initial registration or a subsequent home surveillance visit, newly identified pregnant women (by urine testing) will be approached by one of our 56 field team leaders who will provide women with a pamphlet on antenatal, postpartum and infant care, irrespective of their decision to participate in the trial. Team leaders will explain the purpose and voluntary nature of the trial, elements of participation and potential risks and benefits, and will seek informed consent from women to participate in the study. Mothers will be asked to consent to (a) take daily a coded micronutrient tablet, to be distributed on a weekly basis at home by visiting project staff, and (b) participate in periodic health interviews and assessments of herself and, after delivery, of her infant(s).

5.1.4 Weekly Supplementation

Within days of giving consent, pregnant women will be visited by one of the 596 female distributing staff who will provide the first week’s supplements, plus a spare week’s worth of supplements to allow for unscheduled misses during weekly home visits. Supplements will be provided in a personal plastic bottle with screw top. Women will be advised to keep the bottle out of reach of children at all times. Thereafter, women will be visited usually by the same female supplement distributor each week who will restock supplements (to a count of 14 tablets), monitor compliance, and record

any change in pregnancy or vital status of the mother, fetus or infant, through 12 completed weeks after termination of pregnancy. Plastic bottles and any remaining supplements will be retrieved at the 13 week postpartum home visit, after which weekly visits will cease (although maternal and infant vital and health status will again be assessed at a 6-month postpartum visit – see below).

5.1.5 First Trimester Assessment

A first trimester pregnancy enrollment interview will be conducted usually within a week of consent by one of 56 trained interviewers. The assessment will consist of a 30-day and nested 7-day morbidity history, a 7-day recall of food intake, tobacco use and work activity, a pregnancy history, a questionnaire on parental vital status and morbidity, and a household socioeconomic status assessment (e.g., education, and selected asset and land ownership). Maternal anthropometric assessment will include weight, height, and mid-upper arm circumference (AC).

5.1.6 Third Trimester Assessment

At ~20 weeks after the first trimester interview (~32 weeks gestation), trained interviewers will repeat the home-based assessment, obtaining 30- and 7-day morbidity, and 7-day dietary intake, tobacco use and work activity histories. Maternal anthropometry will include weight and AC.

5.1.7 Birth Assessment

To reach newborns in a setting where ~95% of all births occur at home, a local system, developed previously, will be employed whereby family members or neighbors notify their local project distributor staff of a birth, who in turn will notify lay interviewers who will visit the home to measure the newborn and interview the mother. Infant size will be assessed by anthropometry (weight, length, and arm, chest and head circumference). Mothers will be interviewed about any illnesses, complications and care received during the month before delivery, including during labor and delivery, and about prelacteal and early breast feeding practices of the infant in the first hours after birth. Maternal arm circumference will be again measured at this visit. Based on new evidence of efficacy in reducing infant mortality from a prior trial (JiVitA-2, CHR #: H.22.03.01.09.A1), newborns will also be dosed with 50,000 IU of vitamin A.

5.1.8 Maternal and Infant 1 Month Postpartum Assessment

During the 5th week postpartum, project interviewers will return to participating mothers to obtain a maternal history of morbidity symptoms, complications and any care received during the first 4 weeks after delivery, with emphasis on the first week postpartum. Seven day diet, tobacco use and work activity questions will be repeated, and mothers will be measured for weight and AC. Mothers will be asked about breast and complementary feeding practices, and about illnesses and bleeding symptoms[1] that

1 We are asking about bleeding symptoms in order to investigate the possible, but presently unknown, importance of early infantile bleeding as a form of morbidity in rural

may have been experienced by the infant in the 1st four weeks of life. Infant weight, length and circumferential measurements will be repeated at this visit.

5.1.9 Maternal and Infant 3 Month Postpartum Assessment:

At 13 weeks postpartum, project interviewers will return to conduct a maternal 30-day and nested 7-day history of morbidity, and a 7-day assessment of dietary intake, tobacco exposure and work activity. Maternal weight and AC will also be measured at this visit. Interviewers will obtain a 2-month (i.e., covering the 2nd and 3rd months of life) history of infant breast and complementary feeding practices and illness (including sudden bleeding) symptoms). Infant anthropometry will include weight, length and circumferential measurements of the head, chest and arm.

5.1.10 Maternal and Infant 6 Month Postpartum Assessment:

At 26-weeks postpartum, or ~3 months after ceasing maternal supplementation, interviewers will return to carry out an abbreviated assessment of mothers and infants. Maternal arm and infant head, chest and arm circumferences will be measured. Breast-feeding status of infants will also be noted.

5.1.11 Adverse Vital Outcome Assessment

Miscarriages, induced abortions, still births and maternal and infant deaths will be ascertained each week by local female staff who will report such events at weekly, local (team) staff meetings. Approximately 1 month after reported miscarriages, abortions and stillbirths, team leaders will dispatch trained interviewers to participant homes to obtain histories of events leading up to the pregnancy loss. On report of a maternal or infant death, morbidity and other events occurring prior to death will be ascertained by physicians (MBBS) or lay interviewers, respectively, via the conduct of a verbal autopsy interview with family members of the deceased. Later, verbal autopsy forms will be reviewed independently by 2 physicians who will assign proximal and underlying causes of death following a combination of algorithms and clinical judgment, based on review procedures we have developed during previous trials.

5.1.12 Birth Defect Surveillance System

We do not expect maternal micronutrient supplementation at recommended dietary levels that begin at ~8 weeks's gestation to alter risks of birth defects. However, as a safety measure, we plan to monitor the occurrence of externally apparent birth defects building on experiences of previous trials in Bangladesh. A population- and trained lay-worker-based, physician-backed, digital-photography validating birth defect surveillance system will be implemented throughout the trial population area. At the 1-month postpartum assessment visit trained female interviewers will systematically examine infants from head-to-foot for evidence of external abnormalities. Any trait perceived as abnormal will be noted. Positive reports will be followed-up by home visits carried out by a physician or trained health technician who will examine infants, note clinical findings and document evident physical defects by high-resolution digital

---

breast fed populations, that could lead to a need to address and prevent vitamin K deficiency in the future

photographs that will be entered into a secure database for later expert review, diagnosis and classification.

5.2 JiVitA-3 Substudies Procedures

5.2.1 Substudy Field Procedures

Four to six months after the planned start-up of the supplementation trial, a set of more intensive clinical, biochemical and biophysical studies are planned to be conducted among a subsample of mothers and infants. Approximately 600 full-protocol -compliant mothers with live born infants are required per supplement group (total N=1200) to compare groups of mothers and infants with respect to a range of continuous outcomes, accepting probabilities of type I and II errors of 0.05 and 0.20, respectively. Specific outcomes of interest that could be affected by different maternal micronutrient supplement regimens will include between treatment group-changes that may occur from the 1st to the 3rd trimesters, and from either trimester of pregnancy through 3 months post-partum in (a) multiple maternal plasma micronutrient, acute phase protein, and antioxidant concentrations, (b) breast milk concentrations of micronutrients, immune and other nutritional biomarkers, and antioxidants, and (c) maternal weight and body composition (by anthropometry and bioelectrical impedance analysis, or BIA). Beyond measures of growth taken in the larger trial, substudy infants will also be compared by treatment group with respect to differences in (a) size and body composition at birth and at 1, 3 and 6 months of age by combinations of anthropometry and non-invasive bioelectrical impedance analysis (BIA), and (b) plasma concentrations of sentinel micronutrients and other biomarkers.

Enrollment for the substudy is planned to take ~18 months, given that it will begin 6 months after onset of the larger trial. To achieve a total sample size of ~1200 materno-infant substudy pairs through the first 3 postnatal months, the substudies will be conducted in a subset of ~44 contiguous, centrally located sectors (4 “team areas”), chosen to be balanced across the two treatment groups (~22 sectors for each code) and to broadly represent the larger study area with respect to socioeconomic and maternal and infant nutritional characteristics. A sample of 44 sectors will be expected to yield ~11,000 married women of reproductive age (at ~250 women per sector). Given that ~13% will be expected to become pregnant each year (based on previous data), we expect to enroll ~1430 pregnant women in the 1st 12 months and ~620 in the 1st half of the second year (~18 mo enrolment period), giving an expected total number of ~2050 pregnancies enrolled. At an expected pregnancy loss rate of 30% (due to miscarriage, abortion, stillbirth), we expect ~1435 mothers to yield live births, of whom 1350 are expected to have live infants by 3 months of age (at an early infant mortality rate of 60 per 1000 live births). An all-test procedural compliance rate of 85-90% would yield our planned total sample size of ~1200 fully tested, surviving mother-infant pairs at 3 months of age, or 600 per treatment group. It is likely that an additional ~600 mothers will have contributed baseline specimens and, perhaps, half this number (e.g., 300) to have contributed either (both not both) late pregnancy or 3-month post-partum specimens for various reasons. An additional 200 infant blood specimens are likely to be collected from mothers with incomplete specimen collection as well. Specimens

from incomplete protocols will be processed and their data analyzed, as possible, for selected outcomes and making cross-sectional comparisons of treatment groups.
In addition to study-wide procedures described above, substudy field procedures will include additional anthropometric, clinical and biochemical assessments. Maternal venous blood (~8 ml or <2 tsp) will be drawn at the time of the 1st and 3rd trimester home visits, and at 3 months postpartum, from which samples of plasma and buffy coat will be prepared for temporary storage, shipment and subsequent laboratory analyses for micronutrient, acute phase marker and other analyte concentrations. Hemoglobin will also be measured by HemoCue testing at each visit and results shared with mothers. We will offer to measure blood type at the 1st trimester visit and provide this information as a service to mothers. Maternal urine samples (5 ml) will also be collected at each of the two pregnancy visits, which as part of generating a general health profile of mothers will be assessed for protein and sugar levels using a qualitative dipstick test. Aliquots will also be processed, coded, shipped and stored to laboratory archives for future analyses. Manually expressed breast milk samples (6-8 ml) will be collected from mothers at 3 months postpartum for subsequent laboratory nutritional and immunological assays. Infant blood will be drawn at 3 months of age by heel stick to provide a small amount of plasma (~250 □l) for assessing infant status and detectable treatment differences with respect to sentinel micronutrients (e.g., retinol, zinc, ferritin and transferrin receptor concentrations).
Additional maternal biophysical and clinical assessments in substudies will include blood pressure (by sphygmomanometry), body temperature (oral), anthropometry (tricipital and subscapular skinfolds) and non-invasive, multi-frequency bioelectrical impedance analysis (BIA) at 1st and 3rd trimester visits and at 1 and 3 months postpartum. Through family informant procedures (previously developed) we will seek to obtain a late 3rd trimester, prelabor weight measurement. In addition to birth size measures being done across the trial, substudy infant anthropometry will include weight, length, tricipital and subscapular skinfolds and non-invasive BIA at 1, 3 and 6 months of age.
In one-fourth of the substudy area, we also plan to carry out a limited ultrasound measurement of crown-rump length at the 1st trimester visit which will occur at 6-10 weeks' gestation based on reported last menstrual period (LMP). Ultrasound can estimate gestational age (GA) with 3-5 day accuracy. The procedure will be done by a trained study physician who will not identify fetal gender (which can only be assessed accurately after 14 weeks). The reason for obtaining ultrasound is to validate our study-wide distribution of GA estimates which is based on reported LMP and post-hoc analyses, which has in past studies led to a suspected (by unconfirmed) underestimate of GA and overestimation of preterm delivery rates. No fetal ultrasound assessments will be done at later gestational visits.

5.2.2 Storage of Substudy Biospecimens:

In the substudy areas, we plan to collect from an estimated 1200 mothers specimens of blood (8 ml) and urine (5 ul) during the 1st and 3rd trimester home visits, and blood (8 ml) and breast milk (~8 ml) at the 3-month postpartum visit. From ~1200 infants we plan to collect ~400 ul of blood (to obtain ~250 ul of plasma) at 3 months of age. We expect several hundred additional specimens will be collected from mothers

and infants with otherwise incomplete substudy protocols. A total of ~20,000 specimen aliquots are expected to be collected. First biospecimens are not planned to be collected until January 2008. All specimens will be coded; i.e., linked to records of individual subjects through a system of specimen ID numbers. Each person-visit panel of specimens will undergo micronutrient, other nutrient, immune factor and antioxidant analyses at Johns Hopkins to address or support intended aims of the study. We expect, however, to also archive coded aliquots of biospecimens at the Center for Human Nutrition laboratory at Johns Hopkins for future analyses that will address questions of longer-term health outcomes of maternal micronutrient supplementation and other materno-fetal and infant exposures in this trial cohort. To this end, at the time of obtaining consent for biospecimen collection, mothers will be asked to give their permission for us to store specimens for up to a presently expected duration of ten years. Unanalyzed, archived specimens (ie, beyond 2 years after completion of field work) for which consent has been obtained for long-term storage will be maintained at -70 degrees C at the Johns Hopkins Micronutrient Reference Laboratory under the terms of a separate, approved biospecimen repository protocol.

5.3 Procedures: Mapping and Geographic Information System (GIS)

A 20-meter resolution Geographic Information System (GIS) of the entire JiVitA study area was developed between 2000 and 2003 as part of the JiVitA-1 trial. Initially built using publicly available basemaps depicting village road networks and land ownership plots, the current JiVitA GIS continuously updates geospatial locations of over 250,000 landmarks, including participant households and other landmarks of potential environmental, social, health service, or economic utility in the project area. This system facilitates the locating of study houses, and enrolment and tracking of JiVitA study participants for interview and follow-up. The GIS provides ways to monitor supplement compliance, refusal rates and field team performance. Real-time analysis of interview data allows supervisors to discern spatial trends in interviewer data collection errors or other operational problems. The GIS is maintained by six field surveyors using handheld GPS receivers which enable JiVitA to identify and locate ~ 10,000 new houses and a similar number of household deletions and moves each year. The system enables the study area to be updated every two months.

5.4 Data Collection, Quality Control and Supervision

The JiVitA field organization with displayed ratios of field and supervisory staff to be used to carry out the proposed trial is described in **Figure 3**. The four groups of 12 field distributors (FDs) each at the bottom actually represent 56 groups of FDs of this size. The FD will enroll women, carry out pregnancy surveillance, ascertain pregnancies by urine testing, serve as the first visiting staff to households after childbirth, and distribute supplements to women in the community. These women have worked with JiVitA for the past 4 years in this type of field work and data collection. They will undergo new, intensive training for 2 to 3 months prior to launching JiVitA-3 in the new data collection methods.

The second tier of workers comprises 56 trained female interviewers, one to a “team”, who will carry out sequential household interviews and perform anthropometry on

mothers and infants. This cadre of workers has conducted thousands of interviews in the current trials. The FIs meet on a weekly basis with their team leaders and field distributors to discuss data collection forms completion issues, review standard operating procedures, check each other's work for errors and discuss aspects of community relations. Unusual to this conservative and traditional area, every FI rides a project bicycle over a 6-10 sq km work area. A five-member quality control team (not represented in Figure 4), supervised by a master's level social scientist, provides specialized technical supervision and conducts reliability tests of all field interviewers on a periodic basis to maintain data quality as collected. Periodic refresher training based on anonymous distributions is typically held to reinforce techniques and operational definitions across all interviewers.

Fifty-six team leaders provide the first line of direct, daily supervision over each team of 10-12 distributors and one interviewer, a ratio that has proven to be adequate to supervise and manage, organize and guide local field activities. Team leaders cover their local areas on bicycle. Every Thursday, a weekly review meeting will be held by the Team Leader at which he/she will collect and check completed forms, supply field distributors with supplements, discuss and solve field problems, record vital events of the previous week and schedule interviewers for the following week.

The JiVitA study area is divided into 14 “Areas”, each with 4 teams and ~40 sectors that are supervised by Area Coordinators (AC). The AC is a senior, experienced field worker who is responsible for maintaining quality of data collection and the intervention, periodically meeting with community leaders to discuss goals, procedures and progress of the project, checking performance of staff, and serving as a liaison with senior project management.

Routine senior field supervision is provided by a field management team, which is highly experienced in managing large scale field activities. They will be responsible for trouble-shooting routine, higher level problems that arise in the community, planning and implementation of new field procedures, requesting supplies, distributing salaries and providing other managerial support. Overall in-country scientific, managerial and community relations operations of the project are led by a SMT (Senior Management Team) which is comprised of senior, Bangladeshi scientists plus the JHU in-country representative, who are directly responsible to the JHU investigative team.

5.5 Data Management Procedures

The JiVitA data management center is a state-of-art facility with 24 highly trained staff capable of processing 1over 20,000 data records each week, and capacity to store in accordance with accepted research standards of confidentiality.up to five million forms. We anticipate a similar rate volume of forms processing to be required during JiVitA-3. Once forms are collected and reviewed in the field each week, they will be batched by type and transported to the data center with transmittal lists that specify the numbers and identifiers of each form type. Transmittal lists will be checked against forms actually received in the Rangpur office before data are entered using customized

HTML-ASP web interfaces which mimic exactly the paper forms. Consistency, range, and internal validity checks will be run before data is submitted to a streamlined SQL database. Performance tracking software will allow the data center manager to supervise and track individual worker performance by type of form, time of day, and over time. Most data will be entered once, except for identifiers and outcome and substudy forms, all of which are double data entered. A random sample of all other forms will be re-entered each week to estimate a global error rate. Data checking will also be done to ensure correct linkages between forms, and consistency of variables across forms. All data are stored on internally redundant RAID-Array DELL Servers, and data are backed up to secure external hard drives every day. In addition, the database is uploaded to secure servers at Johns Hopkins University on a weekly basis via an encrypted FTP connection.

5.6 Laboratory Analysis Procedures

At Johns Hopkins, maternal plasma specimens collected at the 1st and 3rd trimester visits and at three months postpartum will be analysed for concentrations of multiple individual micronutrients, biomarkers of infection, oxidative stress, lipid and other aspects of nutritional status that may be expected to be affected by differences in micronutrient nutriture, either as presenting at baseline (1st trimester) or achieved by supplementation, using standard assays. Breast milk and urinary analysis will also evaluate micronutrient, other nutritional and immune factor concentrations that may be expected to reflect differences from supplementation regimen throughout pregnancy and the early postpartum period. An archive of aliquots of plasma, breast milk, urine and FTA cards with buffy coat will be established und maintained under a separate repository protocol implemented at the Johns Hopkins Bloomberg School of Public Health.

6.0 DATA ANALYSIS

2Data analysis will be carried out both at Johns Hopkins University and at the JiVitA research center in Rangpur. Data will be analyzed using SAS (ver 9.1 or higher; SAS Institute Inc., Cary NC) and STATA (ver 8 or higher, Stata Press, College Station, TX). Exploratory analysis will examine ranges and consistencies of variable distributions using stem and leaf plots and frequency distributions. The following section illustrates our planned basic approach to evaluating primary aims of the trial.

6.1 Comparison of Baseline Characteristics

Data from the two randomized groups will be analyzed for baseline comparability (e.g., age at entry, socioeconomic status, nutritional status, women’s parity, previous history of miscarriage, stillbirths and infant death among multiparous women; prevalence rates of baseline morbidity, dietary intake and work activity). Rates of missing data for each of the above variables will be examined in-depth to quantify degrees of comparability across supplement groups.

6.2 Comparison of Losses-to-Follow-up

Rates of losses-to-follow-up for primary outcomes will be examined overall and by supplement group. For example, when analyzing mortality as the outcome, loss-to-follow-up will occur when there is no information available on the outcome of a pregnancy or the vital status of an infant known to be born alive.

6.3 Comparison of Compliance Rates

Compliance to supplementation will be defined as the proportion of eligible days during antenatal and postpartum periods for which women report consuming supplements, using pill count data. Mean compliance rates and quartile distributions will be compared across the three treatment groups for each period of time across supplement regimens.

6.4 Addressing the Primary Aim

The primary aim of this trial is to compare mortality rates through six months of age of infants born to mothers who received daily multiple micronutrient supplements to those infants whose mothers received iron + folic acid supplements on a daily basis, representing the current antenatal “standard of care”. Rates of neonatal, postneonatal and 6-month mortality will be included in this analysis.

All primary analyses will be done on an intent-to-treat basis; that is, by randomized groups of infants irrespective of actual maternal supplement compliance. Denominators for infant mortality analysis will comprise numbers of live births in each group. Relative risk (RR) estimates will be calculated for the MM group compared to the iron-folic acid group as the referent. Confidence intervals for RR estimate will be calculated using a Generalized Estimating Equations (GEE) logistic regression model with exchangeable correlation structure in which survival status can be modeled as a function of the supplement code assignment, adjusted for correlation within units of randomization (60, 61) to account for design effect. Confidence intervals that exclude 1.0 will be considered statistically significant. Kaplan-Meier survival analyses will be used with time-to-death as the outcome to test differences in infant survival probabilities by supplement group followed by Cox proportional hazard modeling in situations where adjustment is needed for identified confounders and/or to test effect modification.

Stratified analysis will explore differences that may exist in mortality impact across distributions of major variables such as compliance (reflecting nutrient exposure), characteristics assessed at baseline such as maternal age, parity, height, BMI and measures of socioeconomic status. Analysis will extend to examining differences in infant mortality by gender, birth size, gestational age and other variables collected during pregnancy that would be expected to be unaffected by nutrient supplementation such as season of birth, maternal smoking and other exposures. Multivariate analysis

will adjust for potentially confounding variables shown to be imbalanced between supplement groups at baseline using GEE logistic regression analysis (52).

6.5 Addressing Secondary Aims

Appropriate to an evaluation of a maternal nutrition intervention of substantial policy importance, JiVitA-3 has an extensive array of secondary aims that reflect health, nutrition and survival outcomes in infants, as well as health and nutritional outcomes in mothers, that could plausibly be affected by maternal multiple micronutrient supplementation. Outcomes to be compared by supplement regimen will include (a) rates of stillbirth and perinatal mortality (b) prevalence rates of conditions potentially arising from gestational micronutrient deficiencies including preterm delivery, low birth weight, and being born small for gestational age; (c) incidence rates of maternal obstetric complications by history (e.g., premature rupture of membranes, prolonged labor, hemorrhage, sepsis, eclamptic symptoms, emergency procedures related to child birth); and (d) differences in status (continuous variables), including maternal and infant micronutrient status, anthropometric dimensions (maternal weight gain and body composition changes in pregnancy, birth weight, length, head, chest and arm circumference and neonatal body composition), and (e) differences in prevalence of morbidity conditions in mothers (based on symptomatic reports during pregnancy and postpartum periods) and infants through six months of age. These analyses will enable a more complete evaluation to be carried out of the short-term public health effects of maternal micronutrient supplementation on pregnancy-related outcomes in women and offspring[2].

2 The proposed design and size of JiVitA-3 will also establish a large cohort of infants who will have differed in their micronutrient nutriture during embryonic, fetal and early infancy via maternal supplement receipt, that in the future can serve as a unique resource for documenting longer-term effects on child development and risks of chronic disease, as a means to document the full health and developmental effects of maternal supplementation with multiple micronutrients at RDA levels.

7.0 ETHICAL ISSUES

7.1 Risks and Benefits

The provision of supplementary iron and folic-acid is a nutritional standard of care during pregnancy. All identified pregnant women will receive instructional pamphlets on antenatal and infant care. Pregnant women enrolled into the trial will receive an Institute of Medicine (IOM, USA) recommended dietary allowance (RDA) of iron and folic-acid (24, 25), either as a stand alone supplement or embedded with other nutrients into the multiple micronutrient supplement. Half of the enrolled pregnant women will receive a daily supplement containing an RDA for an additional 13 essential micronutrients, in daily amounts recommended by the IOM in the USA (24, 25). The multiple nutrient mix also closely aligns with a supplement formulation recommended for use by UNICEF in undernourished populations (26).Thus, there is reasonable expectation that participating women and their infants should receive some nutritional and health benefit from participating in the trial, irrespective of treatment arm, related to improved iron and folate status, risk reduction in iron deficiency anemia and perhaps a modest increase in birth weight. Conventional wisdom would predict that these nutritional benefits should reduce risk of infant death and materno-infant health complications in both groups. However, neither the health benefits nor risks of antenatal-postnatal multiple micronutrient supplement use are quantified or understood. A large recent trial in Indonesia has reported a significant 20% decline in infant mortality with antenatal micronutrient supplementation, using a formulation similar to that proposed here (Shankar et al, Micronutrient Forum, Istanbul, Turkey, 2007). On the other hand, two small trials in Nepal unexpectedly found non-significant reductions in neonatal survival following multiple micronutrient supplement use (19,20), possibly related to increased risk of birth asphyxia from a marginally increased birth size (27). We believe that the weak and/or contrasting evidence leads to a state of equipoise about whether or not to promote standard (and already widely used and accepted) antenatal multivitamin and mineral supplement use as a public health measure in South Asia. Infant mortality differences between groups, in either direction, will be regularly monitored through the DSMB review process (see below)

In terms of procedural risks, phlebotomy in women and in infants may cause momentary discomfort, but will be performed by trained workers according to standard medical practice. While popularly considered as a source of exposure to infection, this has not been our experience when carried out with appropriate expertise in trials we have conducted in Bangladesh and Nepal over the past 15 years. The body composition and ultrasound assessments proposed in this study are common, non-invasive and safe for use at all ages. For example, the BIA equipment is powered by a small, 9v transistor radio battery.

Some additional nutritional or other benefits may accompany participation. Since our recently completed JiVitA-2 trial showed that newborn vitamin A can reduce risk of infant

mortality, we plan to offer mothers the opportunity for staff to dose babies born in the trial with 50,000 IU vitamin A. With time we intend to explore how this newborn supplementation opportunity might be extended more broadly to the District of Gaibandha as national policy discussions proceed about this new promising intervention. Infants and their mothers who, at the times of their home-based morbidity interviews, report illnesses that suggest a severe illness (based on predefined criteria for maternal responses to closed-ended questions) will be referred to local clinicians. Women reporting to be night blind in the third trimester of pregnancy will be treated with 25,000 IU of vitamin A weekly for at least 4 weeks, following WHO guidelines. In the substudy area, mothers blood-spot tested for anemia will be supplied with iron-folic acid supplements and followed for response or need for further referral. Mothers will be issued a project birth certificate for each live-born infant shortly after birth. Women who are identified through surveillance as pregnant will receive counseling and a pamphlet on antenatal, postnatal and newborn care, irrespective of participation in the trial. Also, local health center staff will receive periodic continuing education seminars which may lead to improved clinical care, or public health and program skills in the study area.

7.2 Compensation

Subjects will not be compensated for participating in any facet of the trial. Mothers or infants who may be referred to local clinicians for health evaluation will be provided transport costs to clinics in order to remove this particular obstacle to care.

7.3 Disclosure and Consent Processes

Following traditional practices, meetings will first be held to inform leaders, community groups, health officials and local healers about the study, and to request their cooperation. A community brief about the study will be published in local newspapers and circulated throughout the study area. Only married women will be eligible to participate in the trial. Enrolled married women <18 years will be considered “emancipated minors" whose consent will be considered autonomous. Spouses, to the extent possible, will be involved in the consent process. Informed consent from women or their family members will be sought on several occasions, as described in the compendium of consent forms attached to this research plan: that is, at the time of the initial census and pregnancy surveillance (Consent Form 01); before women are administered a pregnancy test at the initial census (Consent Form 02) or during routine pregnancy surveillance (Consent Form 03); when, as pregnant, their participation is sought in the trial to receive supplements and to be periodically assessed (Consent Form 04); prior to investigating a pregnancy loss (Consent Form 05), an infant death (Consent Form 06), a maternal death (Consent Form 07), or a possible birth defect (Consent Form 08); and, prior to subsample women being asked to join enhanced biochemical and clinical assessment protocols (Consent Form 09). All consents are planned to be verbal, following rural tradition, but will be documented on forms by trained field staff which will be signed by a local witness. Signatures or thumb prints of trial subjects will not be used as they are typically reserved for legal contracts, land transfers and other important events. Further, rarely are women asked for signatures.

Subjects will be informed that they can refuse to participate in the study and that they have the right to withdraw from the study at any time. Although there will be no alternative research programs offered, refusal to participate will not affect women's chances of participating in future JiVitA Project activities.

7.4. Confidentiality Assurances

All data linked to identifiers will be treated with strict confidence throughout and beyond the duration of the trial, with identifier-linked data available only to study investigators. Data forms will be kept in secure files while in the field prior to their transmittal to the data entry center. Original data will be stored in a secure, temperature-regulated facility maintained at the Data Management Center in Rangpur. This facility is locked and access to the data is restricted to authorized data management personnel and investigators. The source data will be preserved for 7 years after the close-out of the field data collection period, and then destroyed by shredding. Shredded forms will be dissolved by a local paper mill under the supervision of study personnel to produce recycled paper. Identifiers will remain linked to all entered data given the extended, longitudinal nature of planned studies. All analyses and papers will report results in aggregate so as to prevent individual disclosure. All biochemical specimens will have coded identifiers that will remain with specimens throughout transport and analysis, for which linkage identifiers will remain solely within the data analysis unit in the Center for Human Nutrition. The Principle Investigator (Dr. K. P. West Jr, 410-955-2061) will be responsible for all data security.

7.5 Safety Monitoring

A data and safely monitoring board (DSMB) will be composed with membership representing national and international expertise in obstetrics-gynecology, pediatrics, epidemiology, biostatistics and clinical trial design. Once formalized the IRB at Johns Hopkins and Bangladesh Medical Research Council will be informed of its membership. Once constituted, the Board will be asked to convene within the first 3-4 months of start-up to review the protocol, procedures, study timetable, to agree on terms of reference, timings of meetings, interim analyses and stopping rules. The Board will be asked to convene at least annually throughout the duration nof the trial. A DSMB report will be prepared following each meeting and submitted to the IRB within a month of its preparation.

7.6 Institutional Review

The JiVitA-3 protocol will be submitted to the Johns Hopkins Bloomberg School of Public Health Institutional Review Board and the Bangladesh Medical Research Council in Dhaka for review and approval prior to beginning field research activities. Both institutional review boards will periodically, based on their agency policies, review the progress of the trial, wording, content and use of informed consent procedures and other issues related to the protection of human subjects. Information about the trial will be conveyed through a series of meetings between the JiVitA Project and with

organizations, groups and political and civic leaders at all community levels within the study area. This will enable project staff to approach households to obtain consent from individual women and, whenever possible, their husbands, to participate in the supplementation and assessment activities. An independent, international Data Safety and Monitoring Board (DSMB) composed of experts from relevant fields of public health and clinical sciences, including nutrition, epidemiology/clinical trials, obstetrics and gynecology/maternal health, and neonatology/pediatrics, will be formed and scheduled to meet near the outset of the trial and periodically thereafter (at least annually). The terms of reference for the DSMB will be to review of the quality of implementation and to monitor accruing evidence on the safety and efficacy of the interventions being tested. The DSMB will have confidential access to data in order to conduct its reviews. Following each meeting, the Board will be asked to issue an evaluation report concerning the conduct of the trial and provide recommendations to investigators on appropriateness of its continuance.

8.0 COLLABORATIVE AGREEMENTS

The premix for both supplements will be produced, donated and shipped to Bangladesh by DSM, Ltd in Singapore under technical arrangements with the company's regional and corporate headquarters in Bombay, India and Basel, Switzerland, respectively. The caplets will be produced and donated in-country by Beximco, Ltd. Neither manufacturer has representation on the study team, privileged access to data or exclusive commercial rights to the (generic) supplement formula resulting from collaboration. A total of 16 million supplements will be manufactured, bottled, labeled and delivered to the project for use in the trial free-of-charge. Under agreement with the producers, the trial supplements will be produced in two batches, at the outset and mid-way through the trial, with tests for potency planned to be carried out periodically from supplements sampled at the point of use.

9.0 TIMETABLE

The JiVitA-3 trial is due to begin formal registration and pregnancy surveillance, detection and supplementation activities in September 2007. Pregnancy enrollment is expected to reach the planned number of ~36,000 pregnancies by September 2009. Three month-follow-up of all pregnancies and infants should be completed by or before September 2010. The substudies of JiVitA-3 are due to start-up by January 2008, and be finished in the field by September 2010 as well. **Figure 2** provides an overall timeline for the JiVitA-3 trial.

10.0 REFERENCES

1. Osrin D, de L Costello AM. Maternal nutrition and fetal growth: practical issues in I nternational health. Semin Neonatol 2000;5:209-219.

2. Delange FM. Iodine deficiency disorders in mothers and infants. In: Delange FM, West KP Jr (eds). Micronutrient Deficiencies in the First Months of Life. Nestle Nutrition Workshop Series Pediatric Program. Vevey: Nestec Ltd / Basel: S Karger AG 2003;52:89-102.

3. Black MM. Micronutrient deficiencies and cognitive functioning. J Nutr 2003;133:3927S-3931S.

4. Cobra C, Muhilal, Rusmil K, Rustama D, Djatnika, Suwardi SS, Permaesih D, Muherdiyantiningsih, Martuti S,Semba RD. Infant survival is improved by oral iodine supplementation. J Nutr 1997;127:574-578.

5. Medical Research Council Vitamin Study Research Group. Prevention of neural tube defects: results of the Medical Research Council Vitamin Study. Lancet 1991;338:131-137.

6. Czeizel AE. Reducing risks of birth defects with periconceptional micronutrient supplementation. In: Delange FM, West KP Jr (eds). Micronutrient Deficiencies in the First Months of Life. Nestle Nutrition Workshop Series Pediatric Program. Vevey: Nestec Ltd / Basel: S Karger AG 2003;52:309-325.

7. Christian P, West KP Jr, Khatry SK, LeClerq SC, Pradhan EK, Katz J, Shrestha SR, Sommer A. Effects of maternal micronutrient supplementation on fetal loss and infant mortality: a cluster-randomized trial in Nepal. Am J Clin Nutr 2003;78:1194-1202.

8. Preziosi P, Prual A, Galan P, Daouda H, Boureima H, Hereberg S. Effect of iron supplementation on the iron status of pregnant women: consequences for newborns. Am J Clin Nutr 1998;68:404-405.

9. Christian P, Khatry SK, Katz J, Pradhan EK, LeClerq SC, Shrestha SR, Adhikari RK, Sommer A, West KP Jr. Effects of alternative maternal micronutrient supplements on low birth weight in rural Nepal: double blind randomised community trial. BMJ 2003;326:571-576.

10. Merialdi M, Caulfield LE, Zavaleta N, Figueroa A, Dominici F, DiPietro JA. Randomized controlled trial of prenatal zinc supplementation and the development of fetal heart rate. Am J Obstet Gynecol 2004;190:1106-1112.

11. Sazawal S, Malik P. Preventing zinc deficiency in early infancy: impact on morbidity, growth and mortality. In: Delange FM, West KP Jr (eds). Micronutrient Deficiencies in the First Months of Life. Nestle Nutrition Workshop Series Pediatric Program. Vevey: Nestec Ltd / Basel: S Karger AG 2003;52:143-163.

12. Brooks WA, Santosham M, Naheed A, Goswami D, Wahed MA, Diener-West M, Faruque ASG, Black RE. Effects of weekly zinc supplements on incidence of pneumonia and diarrhea in children younger than 2 years in an urban, low-income population in Bangladesh; randomised controlled trial. Lancet 2005;366:999-1004.

13. Baqui AH, Black RE, El Arifeen S, Yunus M, Chakraborty J, Ahmed S, Vaughan JP. Effect of zinc supplementation started during diarrhea on morbidity and mortality in Bangladeshi children: community randomised trial. BMJ 2002;325:1059-1064.

14. Villar J, Abdel-Aleem H, Merialdi M, Mathai M, Ali MM, Zavaleta N, Purwar M, Hofmeyr J, thi Nhu Ngoc N, Campodonico L, Landoulsi S, Carroli G, Lindheimer M, on behalf of the World Health Organization Calcium Supplementation for the Prevention of Preeclampsia Trial Group. World Health Organization randomized trial of calcium supplementation among low calcium intake pregnant women. Am J Obstet Gynecol 2006;194:639-649.

15. Poston L, Briley AL, Seed PT, Kelly FJ, Shennan AH, for the Vitamins in Pre-eclampsia (VIP) Trial Consortium. Vitamin C and vitamin E in pregnant women at risk for pre-eclampsia (VIP trial): randomised placebo-controlled trial. Lancet 2006;367:1145-1154.

16. Holmes VA, McCance DR. Could antioxidant supplementation prevent pre-eclampsia? Proceed Nutr Soc 2005;64:491-501.

17. Yucesoy G, Ozkan S, Bodur H, Tan T, Caliskan E, Vural B, Corakci A. Maternal and perinatal outcome in pregnancies complicated with hypertensive disorder of pregnancy: a seven year experience of a tertiary care center. Arch Gynecol Obstet. 2005 Nov;273(1):43-9. Epub 2005 Apr 15.

18. Keyes LE, Armaza JF, Niermeyer S, Vargas E, Young DA, Moore LG. Intrauterine growth restriction, preeclampsia, and intrauterine mortality at high altitude in Bolivia. Pediatr Res. 2003 Jul;54(1):20-5. Epub 2003 Apr 16.

19. Christian P, West KP Jr, Khatry SK, Katz J, LeClerq S, Pradhan EK, Shrestha SR. Vitamin A or [illegible]-carotene supplementation reduces but does not eliminate maternal night blindness in Nepal. J Nutr 1998;128:1458-1463.

20. West KP Jr, Katz J, Khatry SK, LeClerq SC, Pradhan EK, Shrestha SR, Connor PB, Dali SM, Christian P, Pokhrel RP, Sommer A, and the NNIPS-2 Study Group. Double blind, cluster randomised trial of low dose supplementation with vitamin A or [illegible]-carotene on mortality related to pregnancy in Nepal. BMJ 1999;318:570-575.

21. Humphrey JH, Agoestina T, Wu L, Usman A, Nurachim M, Subardja D, Hidayat S, Tielsch J, West KP Jr, Sommer A. Impact of neonatal vitamin A supplementation on infant morbidity and mortality. J Ped 1996;128:489-496.

22. Rahmathullah L, Tielsch JM, Thulasiraj RD, Katz J, Coles C, Devi S, John R, Prakash K, Sadanand AV, Edwin N, Kamaraj C. Impact of supplementing newborn infants with vitamin A on early infant mortality: community based randomised trial in southern India. BMJ 2003;327:254-259.

23. Shearer MJ, Zhang HF. Early infant vitamin K deficiency: extent, health consequences and approaches to prophylaxis. In: Delange FM, West KP Jr (eds). Micronutrient Deficiencies in the First Months of Life. Nestle Nutrition Workshop Series Pediatric Program. Vevey: Nestec Ltd / Basel: S Karger AG 2003;52:181-207.

24. West KP Jr. Extend of vitamin A deficiency among preschool children and women of reproductive age. J Nutr 2002;132:2857S-2866S.

25. Singh V, West KP Jr. Vitamin A deficiency and xerophthalmia among school-aged children in southeastern Asia. Eur J Clin Nutr 2004;58:1342-1349.

26. Ahmed F, Khan MR, Karim R, Taj S, Hyderi T, Faruque MO, Margetts BM, Jackson AA. Serum retinol and biochemical measures of iron status in adolescent schoolgirls in urban Bangladesh. Eur J Clin Nutr 1996;50:346-351.

27. Ahmed F, Hasan N, Kabir Y. Vitamin A deficiency among adolescent female garment factory workers in Bangladesh. Eur J Clin Nutr 1997;51:698-702.

28. Christian P, West KP Jr, Khatry SK, Katz J, Shrestha SR, Pradhan EK, LeClerq SC, Pokhrel RP. Night blindness of pregnancy in rural Nepal - nutritional and health risks. Int J Epidemiol 1998;27:231-237.

29. Christian P, Shrestha J, LeClerq SC, Khatry SK, Jiang T, Wagner T, Katz J, West KP Jr. Supplementation with micronutrients in addition to iron and folic acid does not further improve the hematologic status of pregnant women in rural Nepal. J Nutr 2003;133:3492-3498.

30. Christian P, West KP Jr, Khatry SK, Kimbrough-Pradhan E, LeClerq SC, Katz J, Shrestha SR, Dali SM, Sommer A. Night blindness during pregnancy and subsequent mortality among women in Nepal: Effects of vitamin A and [illegible]-carotene supplementation. Am J Epidemiol 2000;152:542-547.

31. Duport N, Preziosi P, Boutron-Ruault MC, Bertrais S, Galan P, Favier A, Lafond JL, Hercberg S. Consequences of iron depletion on health in menstruating women. Eur J Clin Nutr 2003;57:1169-1175.

32. Cuskelly GJ, McNulty H, Scott JM. Fortification with low amounts of folic acid makes a significant difference in folate status in young women: implications for the prevention of neural tube defects. Am J Clin Nutr 1999;70:234-239.

33. Ramakrishnan U, Manjrekar R, Rivera J, Gonzales-Cossio T, Martorell R. Micronutrients and pregnancy outcome: a review of the literature. Nutr Res 1999;19:103-159.

34. Dijkhuizen MA, Wieringa FT, West CE, Muherdiyantiningsih, Muhilal. Concurrent micronutrient deficiencies in lactating mothers and their infants in Indonesia. AmJ Clin Nutr 2001;73:786-791.

35. Jiang T, Christian P, Khatry SK, Wu L, West KP Jr. Micronutrient deficiencies in early pregnancy are common, concurrent, and vary by season among rural Nepali pregnant women. J Nutr 2005;135:1106-1112.

36. Priyali P, Umesh K, Kumar KS, Renu S, Anand K, Nandita G, Nand DS, Rajvir S, Pretti S. Prevalence of multiple micronutrient deficiencies amongst pregnant women in a rural area of Haryana. Ind J Ped 2004;71:1007-1014.

37. Delange FM, West KP Jr (eds). Micronutrient Deficiencies in the First Months of Life. Nestle Nutrition Workshop Series Pediatric Program. Vevey: Nestec Ltd / Basel: S Karger AG 2003;52:1-370.

38. Keen CL, Clegg MS, Hanna LA, Lanoue L, Rogers JM, Daston GP, Oteiza P, Uriu-Adams JY. The plausibility of micronutrient deficiencies being a significant contributing factor to the occurrence of pregnancy complications. J Nutr 2003;133:1597S-1605S.

39. Romero R, Chaiworapongsa T, Espinoza J. Micronutrients and intrauterine infection, preterm birth and the fetal inflammatory response syndrome. J Nutr 2003;133:1668S-1673S.

40. De L Costello AM, Osrin D. Micronutrient status during pregnancy and outcomes for newborn infants in developing countries. J Nutr 2003;133:1757S-1764S.

41. Osendarp SJM, van Raaij JMA, Arifeen SE, Wahed MA, Baqui AH, Fuchs GJ. A randomized, placebo-controlled trial of the effect of zinc supplementation during pregnancy on pregnancy outcome in Banglaeshi urban poor. Am J Clin Nutr 2000;71:114-119.

42. Bates CJ. Assessment of micronutrient status in mothers and young infants. In: Delange FM, West KP Jr (eds). Micronutrient Deficiencies in the First Months of Life. Nestle Nutrition Workshop Series Pediatric Program. Vevey: Nestec Ltd / Basel: S Karger AG 2003;52:1-34.

43. National Institutes of Health. State-of-the-Science Conference Statement. Multivitamin-Mineral Supplements and Chronic Disease Prevention. May, 2006.

44. Joachim Bleys, Edgar R Miller, III, Roberto Pastor-Barriuso, Lawrence J Appel, and Eliseo Guallar. Vitamin-mineral supplementation and the progression of atherosclerosis: a meta-analysis of randomized controlled trials. Am J Clin Nutr 2006 84: 880-887

45. Ramakrishnan U, Gonzalez-Cossio T, Neufield LM, Rivera J, Martorell R. Multiple micronutrient supplementation during pregnancy does not lead to greater infant birth size than does iron-only supplementation: a randomized controlled trial in a semirural community in Mexico. Am J Clin Nutr 2003;77:720-725.

46. Friis H, Gomo E, Nyazema N, Ndhlovu P, Krarup H, Kaestel P, Michaelsen KF. Effect of multimicronutrient supplementation on gestational length and birth size: a randomized, placebo-controlled double-blind effectiveness trial in Zimbabwe. Am J Clin Nutr 2004;80:178-184.

47. Kaestel P, Michaelsen KF, Aaby P, Friis H. Effects of prenatal multimicronutrient supplements on birth weight and perinatal mortality: a randomized, controlled, trial in Guinea-Bissau. Eur J Clin Nutr 2005; July 6 advance online publication.

48. Osrin D, Vaidya A, Shrestha Y, et al. Effects of antenatal multiple micronutrient supplementation on birthweight and gestational duration in Nepal: double-blind, randomized controlled trial. Lancet 2005;365:955-962.

49. Christian P, Osrin D, Manandhar DS, Khatry SK, de L Costello AM, West KP Jr. Antenatal micronutrient supplements in Nepal. (letter) Lancet 2005;366:711-712.

50. World Health Organization, World Food Programme, UNICEF. Preventing and controlling micronutrient deficiencies in populations affected by an emergency. Multiple vitamin and mineral supplements for pregnant and lactating women, and for children aged 6 to 59 months. Geneva: World Health Organization, 2006.

51. Christian P, Osrin D, Manandhar DS, Khatry SK, de L Costello AM, West KP Jr. Antenatal micronutrient supplements in Nepal (correspondence). Lancet 2005;366:711-712.

52. Haider BA, Bhutta ZA, Multiple-micronutrient supplementation for women during pregnancy. Cochrane Database of Systematic Reviews 2006; Issue 4, Art No: CD004905, DOI: 10.1002/14651858.CD004905.pub2.

53. Innocenti Micronutrient Research Report #1.—Sommer A. Innocenti Micronutrient Research Report #1, April 17-20, 2005 .Retrieved October 15, 2006 from

IVACG/International Life Sciences Institute Web site: http://ivacg.ilsi.org/file/Innocenti.pdf

54. Institute of Medicine. Dietary Reference Intakes for Vitamin A, Vitamin K, Arsenic, Boron, Chromium, Copper, Iodine, Iron, Manganese, Molybdenum, Nickel, Silicon, Vanadium, and Zinc. Washington, DC: National Academy Press, 2001.

55. Institute of Medicine. Dietary Reference Intakes for Vitamin C, Vitamin E, Selenium, and Carotenoids. Washington, DC: National Academy Press, 2000.

56. UNICEF/WHO/UNU. Composition of a multi-micronutrient supplement to be used in pilot programmes among pregnant women in developing countries. New York, UNICEF, 1999.

57. WHO/WFP/UNICEF. Preventing and controlling micronutrient deficiencies in populations affected by an emergency Joint Statement by the World Health Organization, the World Food Programme and the United Nations Children's Fund. 2006. Retrieved October 15, 2006 from World Health Organization Web site: http://www.who.int/nutrition/publications/WHO_WFP_UNICEFstatement.pdf

58. West KP Jr, Christian P, Klemm R, Labrique A, Rashid M, Shamim AA, Katz J, Sommer A, for the JiVitA Bangladesh Project: The JiVitA Bangladesh Project: Research to improve nutrition and health among mothers and infants in rural South Asia. Sight and Life Newsletter 2006;1:10-14.

59. Cunningham FG, Gant NF, Leveno KJ, Gilstrap LC III, Hauth JC, Wenstrom KD. William's Obstetrics. 21st Edition, McGraw Hill, 2001, New York, USA.

60. Zeger SL, Liang KY. Longitudinal data analysis for discrete and continuous outcomes.Biometrics. 1986;42(1):121-30.

61. Zeger SL, Liang KY. An overview of methods for the analysis of longitudinal data.

Stat Med. 1992;11(14-15):1825-39

Table 1. Antenatal Multiple Micronutrient Supplementation Impacts on Birth Weight, Low Birth Weight and Preterm Delivery and Neonatal/Infant mortality.

Study authors (Reference)	Country/ Population	Study Design/ Groups (sample size) and Treatment Group Comparisons	Main Outcomes: Mean Bx Wt, g (SD) and Difference in B Wt (95% CL)	Main Outcomes: Low Bx Wt, % RR (95% CL)	Main Outcomes: Preterm, % RR (95% CL)	Main Outcomes: Neonatal mortality Rate /1000 births, and Relative Risk (95% CL)	Remarks
Ramakrishnan et al; 2003 (13)	Mexico/ Peri-urban	RCT/<br>Control:Fe (n=323)<br>MM: (n=322)	 2977 (393)<br>2981 (391)<br>-	 8.9<br>8.5<br>-	 6.5<br>7.5<br>-	Not reported.	Adequate general nutritional status. Low LBW rates
Friis et al; 2004 (14)	Zimbabwe/ Harare antenatal clinics, HIV-negative women	RCT/<br>Control: PL (n=361)<br>MM (n=364)<br><br>MM vs. Placebo	 3044<br>3070<br><br>26 (-38, 91)	 9.7<br>7.1<br><br>0.74 (0.45, 1.20)	 16.2<br>12.7<br><br>0.79 (0.55, 1.13)	Not reported.	Women received iron-folic acid; high loss to follow-up
Kaestel et al; 2005 (15)	Guinea-Bissau, antenatal clinics	RCT/<br>Control: FeFA (n=366)<br>MMx1RDA (n=360)<br>MM2xRDA (n=374)<br><br>MM1 vs. FeFA<br>MM2 vs. FeFA	 3022 (2952, 3051)<br>3055 (3000, 3110)<br>3097 (3049, 3145)<br><br>49 (-22, 121)[2]<br>88 (17, 159)[2]	 13.6<br>12.0<br>10.1<br><br>0.88 (0.57, 1.37)[2]<br>0.70 (0.44, 1.11)[2]	Not reported	 42<br>50<br>44<br><br>1.15 (0.63, 2.10) [2]<br>1.09 (0.60, 1.99) [2]	Birth weight missing for 974 infants;
Christian et al; 2003 (7,14)	Nepal/ Rural terai	RCT/<br>Control: VA (n=685)<br>FAFe: (n=635)<br>MM: (n=705)<br><br>FAFe vs. Control<br>MM vs. Control	 2587 (445)<br>2652 (436)<br>2659 (446)<br><br>37 (-16, 90)[1]<br>64 (12, 119)[1]	 43.4<br>34.3<br>35.3<br><br>0.84 (0.72, 0.99)<br>0.86 (0.74, 0.99)	 20.4<br>23.1<br>20.6<br><br>1.13 90.90, 1.40)<br>1.01 (0.82, 1.26)	 45.7<br>36.3<br>54.0<br><br>0.80 (0.50, 1.27)<br>1.19 (0.77, 1.83)	N for mortality is higher: 876, 772 and 870 for C, FAFe and MM
Osrin et al; 2005 (17)	Nepal/ urban/rural terai antenatal clinics	RCT/<br>Control: FeFA (n=523)<br>MM: (n=529)<br><br>MM vs Control	 2733 (422)<br>2810 (529)<br><br>77 (24, 130)	 25<br>19<br><br>0.69 (0.52, 0.93)	 10<br>8<br><br>0.85 (0.57, 1.29)	 20<br>30.6<br><br>1.53 (0.72, 3.23)	N for mortality is 568 (Control) and 571 (MM)

RCT = randomized controlled trial; Low birth weight : <2500 g; Preterm delivery: gestational duration of <37 wk

[1] Adjusted for maternal weight at baseline;

[2] Adjusted for malaria parasitemia, anemia, infant sex, and seasons of birth. (Adapted from Christian and West, A2Z Micronutrient Brief, April 2006)

Table 2. Recommended Dietary Allowances and Recommended, Previously Used and Proposed JiVitA-3 Multiple Micronutrient Supplement Compositions

	Reference: RDA[1] for Pregnancy/ Lactation	NNIPS-3[2]: Previously Used Formula	UNIMMAP[3] :Current UNICEF Formula	UNICEF/ WHO[4]: Emergency Guidelines	**JiVitA-3 Proposed (~1 RDA)** [5]
Vitamin A (µg RE)	750/770	1000	800	800	**770**
Vitamin D (µg)	5 (200 IU)	10	5	5	**5**
Vitamin E (mg)	15	10	10	15	**15**
Vitamin K (µg)	75/90	65	-	-	**90**
Folic Acid (µgDFE)	600	400	400	600	**600**
Thiamin (mg)	1.4	1.6	1.4	1.4	**1.4**
Riboflavin (mg)	1.4	1.8	1.4	1.4	**1.4**
Niacin (mg)	18	20	18	18	**18**
VitaminB-12 (µg)	2.6	2.6	2.6	2.6	**2.6**
Vitamin B-6 (mg)	1.9	2.2	1.9	1.9	**1.9**
Vitamin C (mg)	80/85	100	70	55	**85**
Iron (mg)	27	60	30	27	**27**
Zinc (mg)	12/11	30	15	10	**12**
Iodine (µg)	220	-	150	250	**220**
Copper (µg)	1000	2000	2000	1150	**1000**
Selenium (µg)	60	-	65	30	**60**
Magnesium (mg)	400/350	100	-	-	**-**

[1] Based on a multivolume set of publications entitled “Dietary Reference Intakes” by the Food and Nutrition Board of the Institute of Medicine, Washington DC, from 1997 to 2002.
[2] Christian et al (refs 7,9).
[3] Based on a UNICEF expert consultation in New York, NY in December 1999.
[4] World Health Organization, 2006 (ref 47).
[5] As the comparison group, the antenatal standard of care daily supplement to be used in JiVitA-3 is proposed to contain iron (27 mg) as fumarate, reflecting the content of the UNIMMAP supplement, and folic acid (600 ug), reflecting the current RDA of the Dietary Reference Intake for folic acid set by the Institute of Medicine, Washington DC

Table 3 Sample Size of Live Births and Pregnancies Required per Group (Ni)1

		**Percent Reduction in Infant Mortality (to six months)**				
**6- Mo IMR**	**No. of Births (Bx) and Pregnancies**	**10**	**15**	**20**	**25**	**30**
**50**	No. Bx (unadjusted)	28,806	12,574	6,943	4,360	2,969
	No. Bx (adjusted)[2]	34,912	15,222	8,040	5,278	3,594
	No. Pregnancies[3]	49,875	21,746	11,486	7,540	5,135
**55**	No. Bx (unadjusted)	26,059	11,377	6,282	3,947	2,688
	No. Bx (adjusted)	31,545	13,773	7,605	4,778	3,254
	No. Pregnancies	45,065	19,676	10,865	6,826	4,649
**60**	No. Bx (unadjusted)	23,769	10,379	5,733	3,602	2,453
	No. Bx (adjusted)	28,773	**12,565**	6,940	4,361	2,970
	No. Pregnancies	41,105	**17,950**	9,915	6,230	4,243
**65**	No. Bx (unadjusted)	21,831	9,535	5,267	3,309	2,255
	No. Bx (adjusted)	26,427	11,543	6,376	4,006	2,730
	No. Pregnancies	37,753	16,490	9,109	5,723	3,900
**70**	No. Bx (unadjusted)	20,171	8,811	4,868	3,060	2,085
	No. Bx (adjusted)	24,418	10,666	5,893	3,705	2,524
	No. Pregnancies	34,883	15,238	8,419	5,293	3,607

[1] Sample size estimates based on presented ranges of infant mortality in the standard of care control group and plausible percent reductions in 6-month infant mortality that could result from maternal multiple micronutrient supplementation, accepting probabilities of Type I and Type II errors of 0.05 and 0.20, respectively.
[2] Adjusted for an estimated design effect = 1.15 and a 5% loss to follow-up.
[3] Based on an estimated pregnancy loss rate of 30% due to miscarriage (~12%), menstrual regulation (~14%) and still birth (~4%). Reference: JivitA-1 trial (unpublished, 2006)

Table 4. Multiple micronutrient tablet formulation and amounts.

DSM Nutritional Products

DSM

Product Formulation

Multivit. / Multimin. Film Coated Tablet

8362

	Composition	Label Claim		Overage	Quantities
				%	Mg/Tabl.
1	Vitamin A	770	µg RE	35	
	as Vitamin A Acetate 500				6.92
2	Vitamin D3	5	µg	35	
	as Vitamin D3, Typ 100 CWS/AM				2.70
3	Vitamin E	15.00	mg αTE	5	
	as Vitamin E 75 HP				31.29
4	Vitamin K1	Deleted from formulation			
	as Vitamin K1 5% SD				
5	Vitamin B9 (Folic Acid)	0.6	mg	25	
	as Folic Acid 10% Trituration				7.50
6	Vitamin B1 (Thiamine Mononitrate)	1.40	mg	10	
	as Thiamine Mononitrate				1.54
7	Vitamin B2 (Riboflavin)	1.40	mg	10	
	as Riboflavin TG				1.54
8	Vitamin B3 (Niacinamide)	18.00	mg	5	
	as Niacinamide				18.90
9	Vitamin B12	2.60	µg	30	
	as Vitamin B12 0.1% WS N				3.38
10	Vitamin B6 (Pyridoxol)	1.90	mg	10	
	as Pyridoxine Hydrochloride				2.55
11	Vitamin C	85.00	mg	5	
	as Ascorbic Acid 90% Granulation				99.17
12	Iron	27	mg	0	
	as Ferrous Fumarate				82.14
13	Zinc	12	mg	0	
	as Zinc Sulfate Monohydrate				32.94
14	Iodine	0.22	mg	0	
	as Potassium Iodide				0.29
15	Copper	1.00	mg	0	
	as Cupric Sulfate Anhydrous				2.51
16	Selenium	0.06	mg	0	
	as Sodium Selenite anhydrous				0.13

This Formula is a suggestion of DSM Nutritional Products., Customer Service Lab Pharma.
The formulations suggested herein are based on information, methods and practices believed to be reliable, however, results obtained may vary with manufacturing conditions and techniques utilised. Accordingly, DSM Nutritional Products can make no guarantees or warranties or assume any responsibility as to the results to be obtained, but provides the aforesaid as a service to you, subject to your judgement and decision to manufacture and/or use the same. For further assistance, please contact your DSM Nutritional Products representative.

13 August 2007

DSM Nutritional Products

Product Formulation

DSM

Multivit. / Multimin. Film Coated Tablet

8362

		Label Claim		Overage	Quantities
				%	Mg/Tabl.
17	Tablettose 80 (1)				150.00
18	Aerosil 200 (2)				4.28
19	Polyplasdone XL 10 (Crospovidone) (3)				7.00
20	Stearic Acid (4)				2.00
21	Magnesiumstearate (5)				2.00
22	Avicel PH 102 (6)				338.52
				**Total Tablet Weight**	**800.00**

Suppliers of excipients

(1) Fischer Chemicals AG, Riesbachstrasse 57, CH-80345 Zürich
(2) Aerosil 200: Aerosil 200, Degussa AG, 40402 Düsseldorf, Germany
(3) Polyplasdone XL 10: ISP Technologies Inc., Wayne, New Jersey
(4) Stearic Acid: Schlüter GmbH Hamburg
(5) Magnesium Stearate: Tracomme AG, CH-8134 Adliswil
(6) Avicel PH 102: FMC Europe NV, Avenue Louise 480 B9, 1050 Brussels, Belgium

Procedure

I Pass 2, 4-10, 12-19 and 22 trough 1.0 mm sieve, add 1,3,11and mix for 20 mins
II Sieve pos. 20 and 21 trough 1.0 mm and mix with **I** for 5 min.
III Compress to tablets

Table 5. Numbers of Supplements Required for JiVitA-3

				Minimum Required Numbers of Supplements (000)		
IMR	%↓	Sample Size Group	Total	Total	MM	Fe + FA
60	**15**	**18,000**	**36,000**	**14,454**[1]	**7,227**	**7,227**
60	20	9,915	19830	7600[2]	3,800	3,800
65	15	16,490	32980	12,640[3]	6,320	6,320
65	20	9,109	18218	7200[4]	3,600	3,600

[1] Number = (17,950 x 365 d) x 1.10 (overage) = ~7.86 m (or ~8 m) per group
[2] (9,915 x 365 d) x 1.20 = ~ 4.34 7.2 per group
[3] (16,490 x 365 d) x 1.20 =6.2 m per group
[4] (9,109 x 365 d) x 1.20 = 3.33 m per group

Figure 1. JiVitA-3 Design

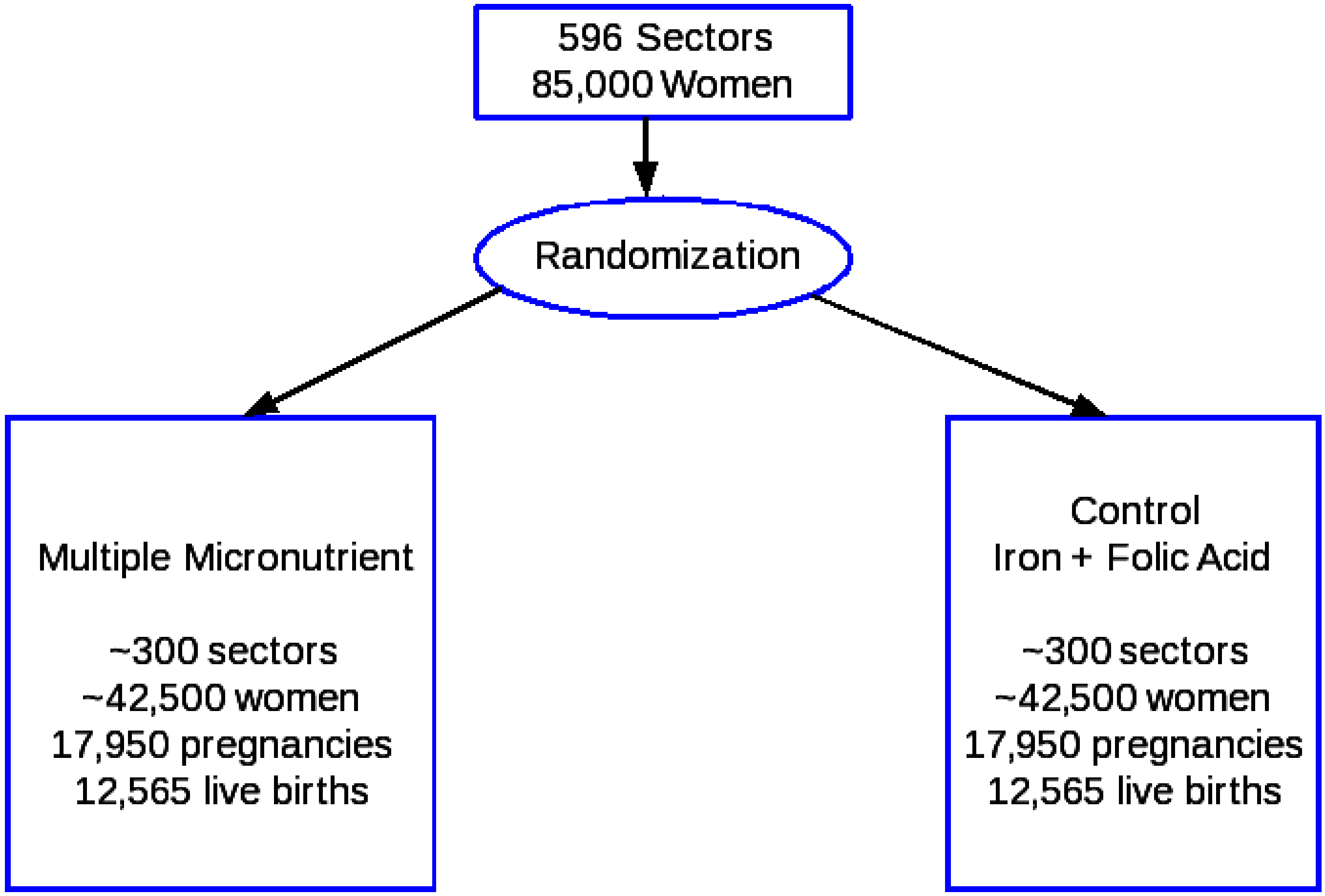

Figure 2. Proposed JiVitA Trial Timetables (Calendar Years)

	2007				2008				2009				2010			
Tasks	Q 1	Q 2	Q 3	Q 4	Q 1	Q 2	Q 3	Q 4	Q 1	Q 2	Q 3	Q 4	Q 1	Q 2	Q 3	Q 4
J 3 Protocol reviews, approvals and funding		X	X													
Supplement Production	X	X	X						X							
Finalize Forms and Procedures		X	X													
Staff Training & Standardization	x	X	X													
Population Census, Registration, Initial Assessment			X													
Pregnancy Surveillance, Detection, Enrollment			X	X	X	X	X	X	X	X	X					
Supplementation of Women & Follow-up			X	X	X	X	X	X	X	X	X	X	X	X	X	
Data Entry and Management			X	X	X	X	X	X	X	X	X	X	X	X	X	
Data and Safety Monitoring Meetings				X				X				X			X	
Laboratory analyses: serum, breast milk, urine					X	X	X	X	X	X	X	X	X	X	X	X
Data Analysis and Reporting Major Findings														X	X	X
Preparation and Publication of Manuscripts															X	X

Figure 3. JiVitA Staff Organization

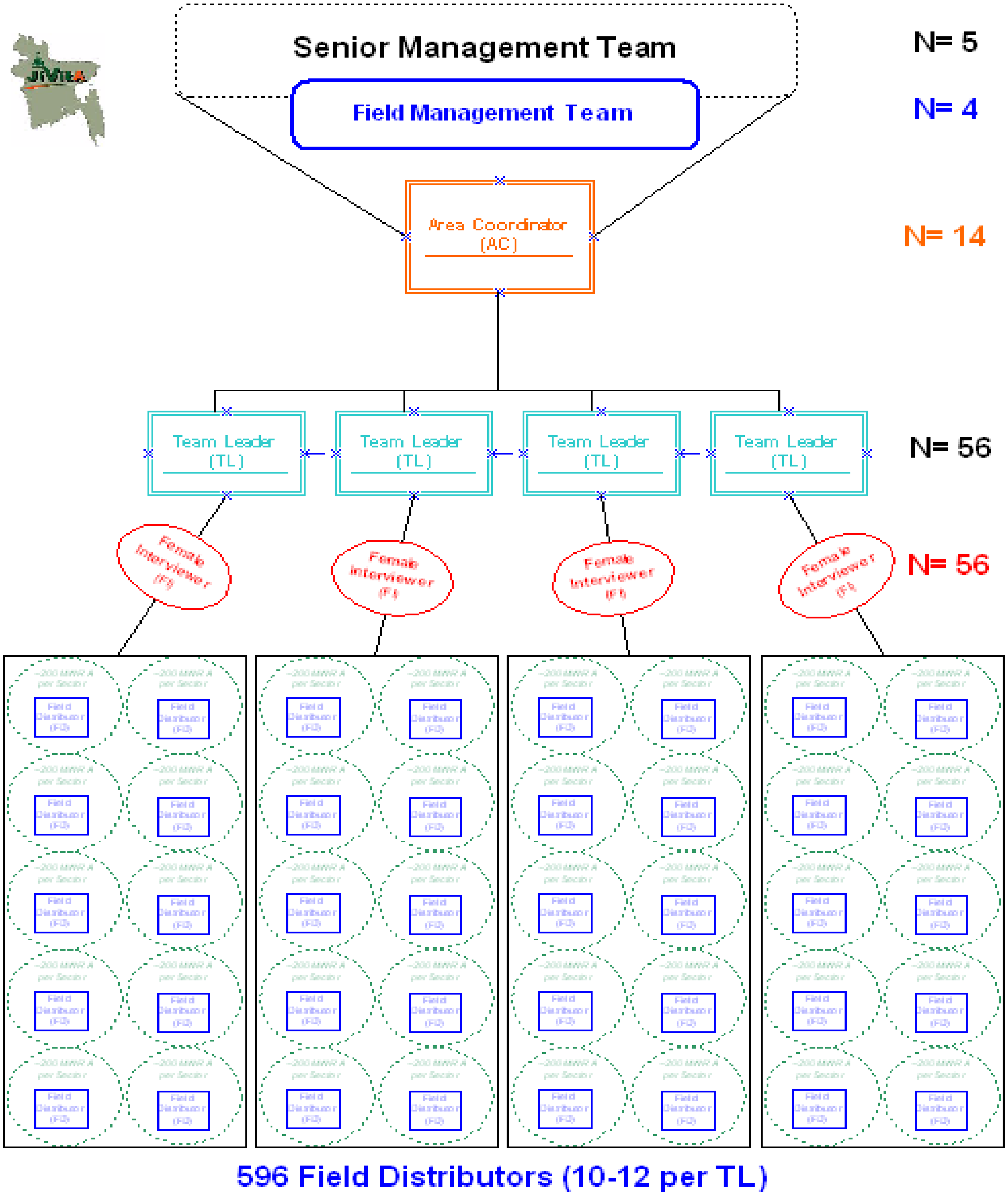

2010
