# Supplementary material for: Effects of Prenatal Multiple Micronutrient Supplementation on Fetal Growth Factors: A Cluster-Randomized, Controlled Trial in Rural Bangladesh
Source: PLoS One. 2015 Oct 2;10(10):e0137269. doi: 10.1371/journal.pone.0137269 (PMC4591978; doi:10.1371/journal.pone.0137269)
Supplement: S1 Protocol — Parent Trial Protocol and Amendment. (ZIP) [file pone.0137269.s002.zip › Trial Protocol/Trial Protocol Ammendment - supplemental information1b.pdf]

RESEARCH PLAN

Study Title
JiVitA-3: Antenatal Multiple Micronutrient Supplementation to Improve Infant Survival and Health in Bangladesh: A Double-Masked, Cluster-Randomized, Controlled Trial

1. Research Question(s):

Primary: Will daily maternal multiple micronutrient (MM) supplementation (containing 15 essential vitamins and minerals at recommended dietary levels) from early pregnancy through 12 weeks postpartum reduce infant mortality through six months of age by ≥15% compared to the mortality of infants whose mothers receive daily iron + folic acid (i.e. the control group)?

Secondary: The design and size of the proposed trial will also allow us to assess the efficacy of maternal multiple micronutrient supplementation in:

1. Reducing incidence rates of
   - Stillbirth by ≥23%, from an expected control group rate of 3%;
   - Preterm birth by ≥10%, from an expected control group rate of 18%;
   - Low birth weight (<2500 g) by ≥5%, from an expected control group rate of 40%.

2. Reducing prevalence rates of
   - Maternal morbidity during pregnancy through the first three postnatal months, including indicators of labor and delivery complications;
   - Maternal anemia and micronutrient deficiencies in the 3rd trimester and at 3 months postpartum;
   - Neonatal and post-neonatal morbidity, as ascertained by interview-based indicators of sepsis, birth asphyxia, hypothermia and other types of illness through three months of age.

3. Influencing
   - Infant multiple micronutrient status at 3 months of age;
   - Linear and ponderal growth of infants during the first 12 months of life;
   - Maternal body composition (by changing relative lean mass) during and following pregnancy;
   - Infant body composition (by changing relative lean mass) during the first 6 months of life;
   - Micronutrient transfer from the mother to fetus during pregnancy;
   - Aspects of materno-placento-fetal function that may influence intrauterine growth such as maternal plasma volume expansion, placental size, and endocrine hormone production.

2. Rationale: Maternal deficiency in multiple essential micronutrients is likely to be a major public health problem in low-income countries (1-5), although collection of prevalence data on coexisting deficiencies remains an urgent need. Micronutrient adequacy is required to support normal physiologic changes and increased metabolic demands associated with placentation, maternal tissue expansion and fetal growth and development during pregnancy. Multiple deficiencies can typically coexist which is believed to cause poorer health in mothers and their infants while *in utero* and in postnatal life (6-11). Supplementing mothers with certain individual micronutrients has been shown to confer health benefits. For example, dietary supplements containing vitamin A can reduce maternal night blindness during pregnancy (12), iodine can reduce risk of iodine deficiency disorders (13), calcium can reduce eclampsia (14), and iron and folic acid can reduce anemia (15).

In the developing world, there is growing international advocacy from UNICEF and the World Health Organization (WHO) to promote daily multiple micronutrient (MM) supplement use to control co-existing maternal micronutrient deficiencies and improve maternal and infant health. While theoretically

Downloaded From: http://jama.jamanetwork.com/ by a Penn State Milton S Hershey Med Ctr User on 01/07/2015

rational, evidence supporting widespread antenatal MM supplement use remains surprisingly weak, with only five population-based, randomized controlled trials published to date on efficacy in non-HIV-affected populations in the developing world (16-20). These few trials, for reasons of design constraint (e.g., restricted outcome assessment, inadequate sample size, insufficient duration or timing of nutrient exposure), questionable extent of deficiency in tested populations, or lack of nutritional efficacy for outcomes that have been assessed, have failed to provide clear evidence of efficacy and safety on which to base policy in this vital area of human nutrition, health and supplemental dietary practice. For example, birth size has been often shown to not increase or increase modestly in response to MM supplementation, which can be viewed as a beneficial response (17-20) that should decrease risk of mortality. However, two studies in Nepal, not powered to examine effects on infant mortality, found increased birth weight to be followed by a non-significant increase, rather than decrease, in neonatal mortality (19, 20). Pooling the findings from both trials yielded a statistically significant effect (21), although this combining technique was criticized as inappropriate and prone to study selection bias (22). Since then, early findings from a large "program effectiveness trial" in Indonesia have shown a 20% reduction in mortality among infants of MM vs. iron-folic acid-supplemented mothers (Shankar et al, Micronutrient Forum, Istanbul, Turkey, April, 2007). A Cochrane meta-analysis published in late 2006, which included the above studies and other unpublished trials of relatively small sample sizes, revealed a reduction in low birth weight of ~17% (95% CI: 9%-24%) with antenatal multiple micronutrient supplement use (23), an outcome universally seen as mediating a lower infant mortality risk in the developing world. However, this analysis of mostly modest-sized trials in developing countries also suggested there to be no difference in infant survival over that observed with "standard of care" iron-folic acid supplementation, leaving the public health impact of this intervention uncertain. It is essential that antenatal MM policies be based on evidence of efficacy (benefit with respect to a range of plausible outcomes) and safety (posing minimal risk) before advocating its use in developing countries. But, to date, no trial has been designed and conducted to assess the efficacy of daily antenatal MM supplementation in improving infant survival.

We propose to assess the impact of daily maternal use of a multiple micronutrient supplement, from early pregnancy through twelve weeks postpartum, on infant mortality through six months of age and other infant and maternal health outcomes in a rural area of northern Bangladesh (the JiVitA Project population site). The proposed MM supplement will contain 15 essential vitamins and minerals at recommended dietary levels for pregnancy and lactation (24, 25), which also approximates the nutrient dosages currently recommended by UNICEF and WHO in emergency settings in developing countries (26). All outcomes will be compared to an "active", randomized, double-masked control group in which women will receive a supplement with recommended dietary levels of iron + folic acid (ie, "standard of care") from early pregnancy through twelve weeks post-partum. The findings of the proposed 3-year trial, known as "JiVitA-3" can be expected to inform and help guide antenatal multinutrient supplementation policies in Bangladesh and elsewhere in South Asia in the future.

3. <u>Methods Overview</u>: We propose to conduct a cluster-randomized, double-masked, active-placebo controlled trial within an established rural study area located in the districts of Gaibandha and Rangpur in northern Bangladesh. The JiVitA Project employs ~850 trained local staff who are based in 70 field offices, a field station and a project headquarters that also includes data management and GIS centers and administrative offices. The study will be carried out in a contiguous rural project area of ~650 sq km with a resident population of nearly 600 thousand people, divided into 596 previously defined, GIS-mapped and addressed community clusters (called "sectors"), which will serve as our unit of randomization. The 596 sectors have also been previously aggregated into 56 team areas of 10-11 sectors each, each of which will be supervised by a team leader and served by a trained home interview staff member. Each sector will contain ~250 households with a married woman of reproductive age based on extant data from our previous trials. Half (n=298) of all sectors will be randomized for consenting pregnant women to receive

Downloaded From: http://jama.jamanetwork.com/ by a Penn State Milton S Hershey Med Ctr User on 01/07/2015

daily, from early pregnancy through 12 weeks postpartum, one of two coded, caplets of identical color, shape and consistency serving either as (a) an active control supplement containing 27 mg iron and 600 µg folic acid (an antenatal "standard of care") or (b) a MM supplement containing 15 essential vitamins and minerals (including iron-folic acid) containing approximate dietary allowances (RDAs) for the period of pregnancy to lactation as published by the Institute of Medicine (IOM) in the United States (**Table 1**) (24). Within all sectors, we will conduct an initial census followed by 5-weekly pregnancy surveillance among married women of reproductive age. Ascertained pregnant women will be approached for consent, enrolled into the trial and given coded supplements according to their sector allocations. Supplements will be replenished each week through 12 weeks postpartum. Mothers and their infants will be periodically visited through 6 months postpartum to obtain updated information on vital status (including pregnancy outcome), nutritional status, diet, illness, and other risk factors. Data will be collated from the field each week, checked and computer-entered as the trial progresses. Periodic data and safely monitoring will occur throughout the trial. On termination, analysis will initially focus on evaluating between-group differences in fetal loss and infant mortality to assess the potential impact of the antenatal supplement regimen in relation to iron and folic acid supplementation (standard of care).

4. <u>Population</u>: The proposed trial will require a sample size of ~12,565 live born infants per group, or a total of 25,130, to observe a difference of ≥15% in six-month, all-cause mortality between the treatment and control groups (**Table 2**). This sample size is based on an expected mortality rate of 60 deaths per 1000 live births in the control group, based on previous data in the study population (i.e., ~75 per 1000) adjusted for time and a 15% reduction that we anticipate occurring after newborns also receive a 50,000 IU dose of vitamin A shortly after birth, an intervention that we have previously shown can reduce infant mortality in this population (R Klemm et al, Pediatrics 2008; CHR #: H.22.03.01.09.A1). The sample size is also based on a design effect of 1.15, a 5% loss to follow-up, and Type I and II error probabilities of 0.05 and 0.20, respectively. We expect there to be a 30% pregnancy loss rate due to spontaneous and induced abortion and stillbirth in this population (JiVitA Project, unpublished 2007) which has been taken into consideration in estimating our final sample size of 17,950 pregnancies per group, for a total of 35,900 (rounded to 36,000) enrolled pregnancies (Table 2). Based on existing census data and our prior recruitment experience in this population, we expect to recruit and supplement ~18,000 pregnant women per year; thus, pregnancy enrollment can be expected to take 2 years (24 months). Given that we expect to recruit mothers at an average of ~8 weeks' gestation, women will be supplemented for ~30 weeks of pregnancy and for another 12 weeks post-partum. Women who after being tested positive for pregnancy are not found or met for 12 consecutive weeks to obtain consent for supplementation and enrollment will be excluded from the study. As we are planning to follow infants and mothers through six months (26 weeks) after childbirth, or 14 weeks beyond the end of maternal supplementation, we expect field work to be completed ~1 year (56 weeks) after reaching our planned sample size of 36,000 pregnancies, yielding a total expected time in the field of ~3 years from the time of starting recruitment.

5. <u>Procedures:</u> We will implement a set of procedures that will involving registering and periodically visiting women of reproductive age (i.e., ~13 to 45 years) in the 596 study sectors for occurrence pregnancy, and then enrolling, supplementing and following pregnancies for vital and morbidity outcomes.

<u>Initial Registration:</u> At the outset of the trial, we will conduct an initial census, registration and pregnancy ascertainment of an estimated 130,000 married women of reproductive age who are residing with their husbands. At this time, we plan to collect limited data on their SES, parental vital status and arm circumference size (reflecting nutritional status). Pregnancy will be ascertained by asking women if they are pregnant and by eliciting a history of menstruation in the previous month, following procedures we have developed previously in Nepal and Bangladesh. Since the trial seeks to enroll and supplement

Downloaded From: http://jama.jamanetwork.com/ by a Penn State Milton S Hershey Med Ctr User on 01/07/2015

women as early as possible in pregnancy, women who respond that they know they are pregnant (thus likely to be relatively advanced in pregnancy), or who are breast feeding an infant <12 months of age (thus, not likely to be menstruating due to lactational amenorrhea) will be temporarily excluded from entry into the pregnancy surveillance system until they either report resuming menstruation or enter their second year postpartum, whichever occurs first. At the time of the initial census, registered, women who have not menstruated in the past month but also do not know if they are pregnant (thus, possible early gestation pregnancies) will be offered a urine test and, if positive, asked to enroll into the trial (see below). Otherwise, registered non-pregnant women will be asked to participate in a pregnancy surveillance system.

Pregnancy surveillance: After the initial registration round, 115-120,000 consenting women (who were not initially pregnant) will be visited every five weeks at home by our field team of 596 local female workers (~1 per sector) over the next 2 to 3 years. During these home visits, usually lasting a few minutes, women will be asked about menstruation in the previous month. Those reporting to be amenstrual will be offered a urine test. Test-positive women will be asked to enroll into the trial (see below). Women who report to have entered menopause, who have become divorced or whose husbands have died will be excluded from subsequent pregnancy surveillance rounds. Otherwise non-pregnant women will continue to be visited at home in the same way until the sample size of ~36,000 pregnancies is attained. During surveillance, newly married women will be asked to be registered into the surveillance system, tested for pregnancy if not menstruating, and offered an opportunity to enroll into the trial if identified as pregnant. We expect to register ~10,000 newly pregnant women over the course of the ~2 year surveillance period.

Pregnancy enrollment: Within a week of the initial registration or a subsequent home surveillance visit, newly identified pregnant women (by urine testing) will be approached by one of our 56 field team leaders who will provide women with a pamphlet on antenatal, postpartum and infant care, irrespective of their decision to participate in the trial. Team leaders will explain the purpose and voluntary nature of the trial, elements of participation and potential risks and benefits, and will seek informed consent from women to participate in the study. Mothers will be asked to consent to (a) take daily a coded micronutrient tablet, to be distributed on a weekly basis at home by visiting project staff, and (b) participate in periodic health interviews and assessments of herself and, after delivery, of her infant(s).

Weekly supplementation: Within days of giving consent, pregnant women will be visited by one of the 596 female distributing staff who will provide the first week's supplements, plus a spare week's worth of supplements to allow for unscheduled misses during weekly home visits. Supplements will be provided in a personal plastic bottle with screw top. Women will be advised to keep the bottle out of reach of children at all times. Thereafter, women will be visited usually by the same female supplement distributor each week who will restock supplements (to a count of 14 tablets), monitor compliance, and record any change in pregnancy or vital status of the mother, fetus or infant, through 12 completed weeks after termination of pregnancy. Plastic bottles and any remaining supplements will be retrieved at the 13 week postpartum home visit, after which weekly visits will cease (although maternal and infant vital and health status will again be assessed at a 6-month postpartum visit – see below).

First trimester assessment: A first trimester pregnancy enrollment interview will be conducted usually within a week of consent by one of 56 trained interviewers. The assessment will consist of a 30-day and nested 7-day morbidity history, a 7-day recall of food intake, tobacco use and work activity, a pregnancy history, a questionnaire on parental vital status and morbidity, and a household socioeconomic status assessment (e.g., education, and selected asset and land ownership). Maternal anthropometric assessment will include weight, height, and mid-upper arm circumference (AC).

Downloaded From: http://jama.jamanetwork.com/ by a Penn State Milton S Hershey Med Ctr User on 01/07/2015

Third trimester assessment: At ~20 weeks after the first trimester interview (~32 weeks gestation), trained interviewers will repeat the home-based assessment, obtaining 30- and 7-day morbidity, and 7-day dietary intake, tobacco use and work activity histories. Maternal anthropometry will include weight and AC.

Birth assessment: To reach newborns in a setting where ~95% of all births occur at home, a local system, developed previously, will be employed whereby family members or neighbors notify their local project distributor staff of a birth, who in turn will notify lay interviewers who will visit the home to measure the newborn and interview the mother. Infant size will be assessed by anthropometry (weight, length, and arm, chest and head circumference). Mothers will be interviewed about any illnesses, complications and care received during the month before delivery, including during labor and delivery, and about prelacteal and early breast feeding practices of the infant in the first hours after birth. Maternal arm circumference will be again measured at this visit. Based on new evidence of efficacy in reducing infant mortality from a prior trial (JiVitA-2, CHR #: H.22.03.01.09.A1), newborns will also be dosed with 50,000 IU of vitamin A.

Maternal and infant 1 month postpartum assessment: During the 5th week postpartum, project interviewers will return to participating mothers to obtain a maternal history of morbidity symptoms, complications and any care received during the first 4 weeks after delivery, with emphasis on the first week postpartum. Seven day diet, tobacco use and work activity questions will be repeated, and mothers will be measured for weight and AC. Mothers will be asked about breast and complementary feeding practices, and about illnesses and bleeding symptoms[1] that may have been experienced by the infant in the 1st four weeks of life. Infant weight, length and circumferential measurements will be repeated at this visit.

Maternal and infant 3 month postpartum assessment: At 13 weeks postpartum, project interviewers will return to conduct a maternal 30-day and nested 7-day history of morbidity, and a 7-day assessment of dietary intake, tobacco exposure and work activity. Maternal weight and AC will also be measured at this visit. Interviewers will obtain a 2-month (i.e., covering the 2nd and 3rd months of life) history of infant breast and complementary feeding practices and illness (including sudden bleeding) symptoms). Infant anthropometry will include weight, length and circumferential measurements of the head, chest and arm.

Maternal and infant 6 month postpartum assessment: At around 26-weeks postpartum, or ~3 months after ceasing maternal supplementation, interviewers will return to measure infant length, weight, and head, chest and arm circumferences. Mothers will be interviewed to obtain breast and complementary feeding patterns and morbidity history of the infant and assess household food security.

Infant 12 month assessment: At around 52-weeks from birth, interviewers will return to assess using the same procedures as at 6 months, household food security, infant weight, length and head, chest and arm circumferences, breast and complementary feeding patterns and morbidity by history.

Adverse vital outcome assessment: Miscarriages, induced abortions, still births and maternal and infant deaths will be ascertained each week by local female staff who will report such events at weekly, local (team) staff meetings. Approximately 1 month after reported miscarriages, abortions and stillbirths, team leaders will dispatch trained interviewers to participant homes to obtain histories of events leading up to the pregnancy loss. On report of a maternal or infant death, morbidity and other events occurring prior to death will be ascertained by physicians (MBBS) or lay interviewers, respectively, via the conduct of a verbal autopsy interview with family members of the deceased. Later, verbal autopsy forms will be

[1] We are asking about bleeding symptoms in order to investigate the possible, but presently unknown, importance of early infantile bleeding as a form of morbidity in rural breast fed populations, that could lead to a need to address and prevent vitamin K deficiency in the future

Downloaded From: http://jama.jamanetwork.com/ by a Penn State Milton S Hershey Med Ctr User on 01/07/2015

reviewed independently by 2 physicians who will assign proximal and underlying causes of death following a combination of algorithms and clinical judgment, based on review procedures we have developed during previous trials.

Birth Defect Surveillance System: We do not expect maternal micronutrient supplementation at recommended dietary levels that begin at ~8 weeks's gestation to alter risks of birth defects. However, as a safety measure, we plan to monitor the occurrence of externally apparent birth defects building on experiences of previous trials in Bangladesh. A population- and trained lay-worker-based, physician-backed, digital-photography validating birth defect surveillance system will be implemented throughout the trial population area. At the 1-month postpartum assessment visit trained female interviewers will systematically examine infants from head-to-foot for evidence of external abnormalities. Any trait perceived as abnormal will be noted. Positive reports will be followed-up by home visits carried out by a physician or trained health technician who will examine infants, note clinical findings and document evident physical defects by high-resolution digital photographs that will be entered into a secure database for later expert review, diagnosis and classification.

JiVitA-3 Field Substudies: Four to six months after the planned start-up of the supplementation trial, a set of more intensive clinical, biochemical and biophysical studies are planned to be conducted among a subsample of mothers and infants. Approximately 600 full-protocol -compliant mothers with live born infants are required per supplement group (total N=1200) to compare groups of mothers and infants with respect to a range of continuous outcomes, accepting probabilities of type I and II errors of 0.05 and 0.20, respectively. Specific outcomes of interest that could be affected by different maternal micronutrient supplement regimens will include between treatment group-changes that may occur from the 1st to the 3rd trimesters, and from either trimester of pregnancy through 3 months post-partum in (a) multiple maternal plasma micronutrient, acute phase protein, and antioxidant concentrations, (b) breast milk concentrations of micronutrients, immune and other nutritional biomarkers, and antioxidants, and (c) maternal weight and body composition (by anthropometry and bioelectrical impedance analysis, or BIA). Beyond measures of growth taken in the larger trial, substudy infants will also be compared by treatment group with respect to differences in (a) size and body composition at birth and at 1, 3 and 6 months of age by combinations of anthropometry and non-invasive bioelectrical impedance analysis (BIA), and (b) plasma concentrations of sentinel micronutrients and other biomarkers.

Enrollment for the substudy is planned to take ~18 months, given that it will begin 6 months after onset of the larger trial. To achieve a total sample size of ~1200 materno-infant substudy pairs through the first 3 postnatal months, the substudies will be conducted in a subset of ~44 contiguous, centrally located sectors (4 "team areas"), chosen to be balanced across the two treatment groups (~22 sectors for each code) and to broadly represent the larger study area with respect to socioeconomic and maternal and infant nutritional characteristics. A sample of 44 sectors will be expected to yield ~11,000 married women of reproductive age (at ~250 women per sector). Given that ~13% will be expected to become pregnant each year (based on previous data), we expect to enroll ~1430 pregnant women in the 1st 12 months and ~620 in the 1st half of the second year (~18 mo enrolment period), giving an expected total number of ~2050 pregnancies enrolled. At an expected pregnancy loss rate of 30% (due to miscarriage, abortion, stillbirth), we expect ~1435 mothers to yield live births, of whom 1350 are expected to have live infants by 3 months of age (at an early infant mortality rate of 60 per 1000 live births). An all-test procedural compliance rate of 85-90% would yield our planned total sample size of ~1200 fully tested, surviving mother-infant pairs at 3 months of age, or 600 per treatment group. It is likely that an additional ~600 mothers will have contributed baseline specimens and, perhaps, half this number (e.g., 300) to have contributed either (both not both) late pregnancy or 3-month post-partum specimens for various reasons. An additional 200 infant blood specimens are likely to be collected from mothers with incomplete

Downloaded From: http://jama.jamanetwork.com/ by a Penn State Milton S Hershey Med Ctr User on 01/07/2015

specimen collection as well. Specimens from incomplete protocols will be processed and their data analyzed, as possible, for selected outcomes and making cross-sectional comparisons of treatment groups.

In addition to study-wide procedures described above, substudy field procedures will include one additional visit for a maternal and infant interview and additional anthropometric, clinical and biochemical assessments. Maternal venous blood (~8 ml or <2 tsp) will be drawn at the time of the 1st and 3rd trimester home visits, and at 3 months postpartum, from which samples of plasma, erythrocytes, and buffy coat (1st trimester only) will be prepared for temporary storage, shipment and subsequent laboratory analyses for micronutrient, acute phase marker and other analyte concentrations. Hemoglobin will also be measured by HemoCue testing at each visit and results shared with mothers. We will offer to measure blood type at the 1st trimester visit and provide this information as a service to mothers. Maternal urine samples (5 ml) will also be collected at each of the two pregnancy visits, which as part of generating a general health profile of mothers will be assessed for protein and sugar and pH levels using a qualitative dipstick test. Aliquots will also be processed, coded, shipped and stored to laboratory archives for future analyses. Manually expressed breast milk samples (6-8 ml) will be collected from mothers at 3 months postpartum for subsequent laboratory nutritional and immunological assays. Infant blood will be drawn at 3 months of age by heel stick to provide a small amount of plasma (~250 µl) for assessing infant status and detectable treatment differences with respect to sentinel micronutrients (e.g., retinol, zinc, ferritin and transferrin receptor concentrations).

Additional maternal biophysical and clinical assessments in substudies will include blood pressure (by sphygmomanometry), body temperature (oral), anthropometry (tricipital and subscapular skinfolds) and non-invasive, multi-frequency bioelectrical impedance analysis (BIA) at 1st and 3rd trimester visits and at 1 and 3 months postpartum. Through family informant procedures (previously developed) we will seek to obtain a late 3rd trimester, prelabor weight measurement. In addition to birth size measures being done across the trial, substudy infant anthropometry will include weight, length, tricipital and subscapular skinfolds and non-invasive BIA at 1, 3 and 6 months of age. Also, at 6 months postpartum, we will conduct a maternal interview to obtain a 30-day and 7-day history of morbidity, dietary intake, tobacco exposure and work activity and a 3-month history of infant breast and complementary feeding practices, illness symptoms and vaccination history.

In one-half of the substudy area, we also plan to carry out a limited ultrasound measurement of crown-rump length at the 1st trimester visit which will occur at 6-10 weeks' gestation based on reported last menstrual period (LMP). Ultrasound can estimate gestational age (GA) with 3-5 day accuracy. The procedure will be done by a trained staff member who will not identify fetal gender (which can only be assessed accurately after 14 weeks). The reason for obtaining ultrasound is to validate our study-wide distribution of GA estimates which is based on reported LMP and post-hoc analyses, which has in past studies led to a suspected (by unconfirmed) underestimate of GA and overestimation of preterm delivery rates. No fetal ultrasound assessments will be done at later gestational visits.

<u>JiVitA-A 3 Dark Adaptometry Study</u>: A new procedure will be nested into the present biochemical/clinical substudy to validate a novel, portable dark adaptometer. This non-invasive test requires the subject to wear a pair of lightweight "dark goggles", connected to a laptop, for a period of no more than 10 minutes. This test will assess the intensity of light required to cause a pupillary constriction, a normal physiologic reflex of the eye which may be impaired by vitamin A deficiency. The test begins by exposing the eyes to a bright flash (similar to a camera flash), followed by 8 minutes of wearing the goggles with no stimuli, allowing the subject's eyes to adjust to darkness (dark adaptation period). This is followed by 1 minute of 7 pulses of light of increasing intensity (1 second per pulse, with 10 seconds of rest between pulses). The stimuli are no brighter than a weak incandescent flashlight, likely to cause little

Downloaded From: http://jama.jamanetwork.com/ by a Penn State Milton S Hershey Med Ctr User on 01/07/2015

or no discomfort to the subject. The last 1-2 minutes of the pupillary reaction will be digitally recorded using the goggles and stored as a video feed of one eye. A total of 400 women already participating in the biochemical substudy will be asked to participate in this new procedure at their late pregnancy visit. The video data will be stored on the secure JiVitA database for subsequent investigator review only.

JiVitA-3 Placenta and Cord Blood Substudy: A new substudy will be nested into the existing biochemical/clinical substudy to assess maternal plasma volume expansion, obtain an umbilical cord blood sample, and weigh the placenta in n=310 mothers with live born infants (n=155 per treatment group) to evaluate the effects of the multiple micronutrient supplement on several intermediate materno-placento-fetal outcomes. At an expected 50% pregnancy attrition rate for this particular substudy (~30% due to fetal loss, ~10% refusals, and ~10% losses to follow-up, including missed births), we will plan to inform and recruit 478 women during their pregnancy to achieve the required sample size. Plasma volume expansion will be estimated, in part, from existing BIA, hemoglobin, hematocrit and anthropometric measurements being taken in the first and third trimesters. In this substudy only, these same measurements will be repeated during an added home visit in the second trimester. At this visit, capillary blood will be collected by finger-stick to measure hemoglobin and hematocrit. For this substudy, we will enhance the existing birth notification system to have enrolled families notify local JiVitA staff about the onset of labor so that a project auxiliary nurse midwife (ANM), trained in labor, delivery and emergency obstetric care, can conduct the substudy procedures. If requested by the family, our trained ANMs will also assist in the delivery. After delivery, ~8 ml of umbilical cord blood will be drawn from the placental side of the cord. The placenta will then be trimmed, drained, and weighed. Characteristics of the cord and placenta will be recorded. The placenta and will be returned to the family after assessment to permit the family to complete any cultural practices involving the placenta. Newborn birth weight will be assessed. The 8 ml cord blood sample will be brought to the project laboratory on ice where it will be processed to plasma and stored in liquid nitrogen until shipped and later analyzed for micronutrient and hormone concentrations at Johns Hopkins. During the birth visit, a project vehicle will remain on call to transport the mother and newborn, accompanied by the ANM, to the nearest appropriate government health facility in the event of a medical emergency. Women will receive a baby outfit and a bar of soap (valued at <$3) as a token of appreciation for participating in this enhanced birth assessment visit.

Storage of Substudy and Placenta-Cord Blood Study Biospecimens: In the substudy areas, we plan to collect from an estimated 1200 mothers specimens of blood (8 ml) and urine (5 ul) during the 1st and 3rd trimester home visits, and blood (8 ml) and breast milk (~8 ml) at the 3-month postpartum visit. From ~1200 infants we plan to collect ~400 ul of blood (to obtain ~250 ul of plasma) at 3 months of age. In addition we expect to collect ~310 specimens of cord blood (~8 ml). We expect several hundred additional specimens will be collected from mothers and infants with otherwise incomplete substudy protocols. A total of ~20,000 specimen aliquots are expected to be collected. First biospecimens are not planned to be collected until January 2008. All specimens will be coded; i.e., linked to records of individual subjects through a system of specimen ID numbers. Each person-visit panel of specimens will undergo micronutrient, other nutrient, immune factor, pregnancy-related hormone, and antioxidant analyses at Johns Hopkins to address or support intended aims of the study. We expect, however, to also archive coded aliquots of biospecimens at the Center for Human Nutrition laboratory at Johns Hopkins, as well as a set of plasma and breast milk samples at the Institute for Nutrition, Mahidol University (INMU), Thailand, for future analyses that will address questions of longer-term health outcomes of maternal micronutrient supplementation and other materno-fetal and infant exposures in this trial cohort. Specifically, one aliquot each of maternal plasma(~0.5ml) and breast milk will be transported via liquid nitrogen dry shipper to INMU for assessment of retinol (ie. vitamin A), tocopherols (ie. vitamin E),

Downloaded From: http://jama.jamanetwork.com/ by a Penn State Milton S Hershey Med Ctr User on 01/07/2015

carotenoids, as well as lipids and oxidative stress markers. The remaining biospecimens (plasma, red blood cells, urine, breast milk, etc) will be shipped on liquid nitrogen to Johns Hopkins for analysis of nutritional and health status markers and long-term storage. To this end, at the time of obtaining consent for biospecimen collection, mothers will be asked to give their permission for us to store specimens for up to a presently expected duration of ten years. We propose to request long term storage of all unanalyzed, archived specimens (ie, beyond 2 years after completion of field work) in the micronutrient reference laboratory facility at Johns Hopkins and at INMU under a separate repository protocol that will be submitted to the IRB prior to the end o the current trial for review.

Data Analysis: Data analysis will be conducted by the investigative teams at Johns Hopkins and in Bangladesh at the JiVitA Project site, under the guidance of the principle investigator. Exploratory analyses will examine discreet and continuous, single and joint distributions, for shape, outliers and other interesting features. Baseline (1st trimester) data analysis will seek to establish the comparability of treatment groups. Measures of supplement-taking adherence will be examined and total and daily nutrient exposures estimated. Primary analyses will compare 6-month mortality rates of infants born to mothers receiving multiple micronutrient (MM) supplements with the 6-month mortality rate among infants whose mothers received daily, the active control supplement of iron + folic acid using Generalized Estimating Equations logistic regression analyses to adjust for cluster-randomization. Additional analyses will evaluate effects of MM supplementation on secondary outcomes including (a) rates of stillbirth and neonatal mortality, (b) prevalence rates of conditions potentially arising from gestational micronutrient deficiencies including preterm delivery, low birth weight, and small size for gestational age; (c) incidence rates of maternal obstetric complications by history (e.g., premature rupture of membranes, prolonged labor, hemorrhage, sepsis, eclamptic symptoms, emergency procedures related to child birth); and (d) differences in status (continuous variables), including maternal, fetal (cord blood), and infant micronutrient status, anthropometric dimensions (maternal weight gain and body composition changes in pregnancy, birth weight, length, head, chest and arm circumference and neonatal body composition), and indices of materno-placento-fetal function; and (e) differences in prevalence of morbidity conditions in mothers (based on symptomatic reports during pregnancy and postpartum periods) and infants through six months of age.

6. Risk/Benefits: The provision of supplementary iron and folic-acid is a nutritional standard of care during pregnancy. All identified pregnant women will receive instructional pamphlets on antenatal and infant care. Pregnant women enrolled into the trial will receive an Institute of Medicine (IOM, USA) recommended dietary allowance (RDA) of iron and folic-acid (24, 25), either as a stand alone supplement or embedded with other nutrients into the multiple micronutrient supplement. Half of the enrolled pregnant women will receive a daily supplement containing an RDA for an additional 13 essential micronutrients, in daily amounts recommended by the IOM in the USA (24, 25). The multiple nutrient mix also closely aligns with a supplement formulation recommended for use by UNICEF in undernourished populations (26).Thus, there is reasonable expectation that participating women and their infants should receive some nutritional and health benefit from participating in the trial, irrespective of treatment arm, related to improved iron and folate status, risk reduction in iron deficiency anemia and perhaps a modest increase in birth weight. Conventional wisdom would predict that these nutritional benefits should reduce risk of infant death and materno-infant health complications in both groups. However, neither the health benefits nor risks of antenatal-postnatal multiple micronutrient supplement use are quantified or understood. A large recent trial in Indonesia has reported a significant 20% decline in infant mortality with antenatal micronutrient supplementation, using a formulation similar to that proposed here (Shankar et al, Micronutrient Forum, Istanbul, Turkey, 2007). On the other hand, two small trials in Nepal unexpectedly found non-significant reductions in neonatal survival following multiple micronutrient supplement use (19,20), possibly related to increased risk of birth asphyxia from a

Downloaded From: http://jama.jamanetwork.com/ by a Penn State Milton S Hershey Med Ctr User on 01/07/2015

marginally increased birth size (27). We believe that the weak and/or contrasting evidence leads to a state of equipoise about whether or not to promote standard (and already widely used and accepted) antenatal multivitamin and mineral supplement use as a public health measure in South Asia. Infant mortality differences between groups, in either direction, will be regularly monitored through the DSMB review process.

Most women enrolled in the larger trial area deliver in their home without the assistance of a trained birth attendant. Women in the Placenta and Cord Blood Substudy will gain the additional benefit of having an Auxillary Nurse Midwife trained in labor, delivery, and emergency obstetric care present at the time of delivery. The ANM will have basic supplies for handling emergencies. Women will also have the benefit of JiVitA transport to a local health facility in the case of a medical emergency that cannot be managed in the home.

In terms of procedural risks, phlebotomy in women and in infants may cause momentary discomfort, but will be performed by trained workers according to standard medical practice. While popularly considered as a source of exposure to infection, this has not been our experience when carried out with appropriate expertise in trials we have conducted in Bangladesh and Nepal over the past 15 years. The body composition and ultrasound assessments proposed in this study are common, non-invasive and safe for use at all ages. For example, the BIA equipment is powered by a small, 9v transistor radio battery. Drawing cord blood and weighing the placenta *ex-vivo* poses no medical risk to the mother or infant, which will be carried out by medically trained staff.

Some additional nutritional or other benefits may accompany participation. Since our recently completed JiVitA-2 trial showed that newborn vitamin A can reduce risk of infant mortality, we plan to offer mothers the opportunity for staff to dose babies born in the trial with 50,000 IU vitamin A. With time we intend to explore how this newborn supplementation opportunity might be extended more broadly to the District of Gaibandha as national policy discussions proceed about this new promising intervention. Infants and their mothers who, at the times of their home-based morbidity interviews, report illnesses that suggest a severe illness (based on predefined criteria for maternal responses to closed-ended questions) will be referred to local clinicians. Women reporting to be night blind in the third trimester of pregnancy will be treated with 25,000 IU of vitamin A weekly for at least 4 weeks, following WHO guidelines. In the substudy area, mothers blood-spot tested for anemia will be supplied with iron-folic acid supplements and followed for response or need for further referral. Mothers will be issued a project birth certificate for each live-born infant shortly after birth. Women who are identified through surveillance as pregnant will receive counseling and a pamphlet on antenatal, postnatal and newborn care, irrespective of participation in the trial. Also, local health center staff will receive periodic continuing education seminars which may lead to improved clinical care, or public health and program skills in the study area.

7. Compensation: Subjects in the Placenta and Cord Blood Substudy will be given a baby outfit and soap (valued <$3) as a token gift of appreciation at the birth visit. All other subjects will not be compensated for participating in any facet of the trial. Mothers or infants who may be referred to local clinicians for health evaluation will be provided transport costs to clinics in order to remove this particular obstacle to care.

8. Disclosure/Consent Processes: Following traditional practices, meetings will first be held to inform leaders, community groups, health officials and local healers about the study, and to request their cooperation. A community brief about the study will be published in local newspapers and circulated throughout the study area. Only married women will be eligible to participate in the trial. Enrolled married women <18 years will be considered “emancipated minors" whose consent will be considered

Downloaded From: http://jama.jamanetwork.com/ by a Penn State Milton S Hershey Med Ctr User on 01/07/2015

autonomous. Spouses, to the extent possible, will be involved in the consent process. Informed consent from women or their family members will be sought on several occasions, as described in the compendium of consent forms attached to this research plan: that is, at the time of the initial census and pregnancy surveillance (Consent Form 01); before women are administered a pregnancy test at the initial census (Consent Form 02) or during routine pregnancy surveillance (Consent Form 03); when, as pregnant, their participation is sought in the trial to receive supplements and to be periodically assessed (Consent Form 04); prior to investigating a pregnancy loss (Consent Form 05), an infant death (Consent Form 06), a maternal death (Consent Form 07), or a possible birth defect (Consent Form 08); prior to subsample women being asked to join enhanced biochemical and clinical assessment protocols (Consent Form 09) ; and prior to participation in the placenta study protocols (Consent Form 13). All consents are planned to be verbal, following rural tradition, but will be documented on forms by trained field staff which will be signed by a local witness. Signatures or thumb prints of trial subjects will not be used as they are typically reserved for legal contracts, land transfers and other important events. Further, rarely are women asked for signatures. Subjects will be informed that they can refuse to participate in the study and that they have the right to withdraw from the study at any time. Although there will be no alternative research programs offered, refusal to participate will not affect women's chances of participating in future JiVitA Project activities.

9. Safety Monitoring: A data and safely monitoring board (DSMB) will be composed with membership representing national and international expertise in obstetrics-gynecology, pediatrics, epidemiology, biostatistics and clinical trial design. Members are in the process of being identified. Once formalized the IRB and Bangladesh Medical Research Council will be informed of its membership. Once constituted, the Board will be asked to convene within the first 3-4 months of start-up to review the protocol, procedures, study timetable, to agree on terms of reference, timings of meetings, interim analyses and stopping rules. A DSMB report will be prepared following each meeting and submitted to the IRB within a month of its preparation.

9. Confidentiality Assurances: All data linked to identifiers will be treated with strict confidence throughout and beyond the duration of the trial, with identifier-linked data available only to study investigators. Data forms will be kept in secure files while in the field prior to their transmittal to the data entry center. Original data will be stored in a secure, temperature-regulated facility maintained at the Data Management Center in Rangpur. This facility is locked and access to the data is restricted to authorized data management personnel and investigators. The source data will be preserved for 7 years after the close-out of the field data collection period, and then destroyed by shredding. Shredded forms will be dissolved by a local paper mill under the supervision of study personnel to produce recycled paper. Identifiers will remain linked to all entered data given the extended, longitudinal nature of planned studies. All analyses and papers will report results in aggregate so as to prevent individual disclosure. All biochemical specimens will have coded identifiers that will remain with specimens throughout transport and analysis, for which linkage identifiers will remain solely within the data analysis unit in the Center for Human Nutrition. The PI (Dr. K. P. West Jr, 410-955-2061) will be responsible for all data security.

10. Collaborative Agreements: The premix for both supplements will be produced, donated and shipped to Bangladesh by DSM, Ltd in Singapore under technical arrangements with the company's regional and corporate headquarters in Bombay, India and Basel, Switzerland, respectively. The caplets will be produced and donated in-country by Beximco, Ltd. Neither manufacturer has representation on the study team, privileged access to data or exclusive commercial rights to the (generic) supplement formula resulting from collaboration. A total of 16 million supplements will be manufactured, bottled, labeled and delivered to the project for use in the trial free-of-charge. Under agreement with the

Downloaded From: http://jama.jamanetwork.com/ by a Penn State Milton S Hershey Med Ctr User on 01/07/2015

producers, the trial supplements will be produced in two batches, at the outset and mid-way through the trial, with tests for potency planned to be carried out periodically from supplements sampled at the point of use.

We have also established an agreement with the Institute of Nutrition, Mahidol University (Dr. Visith Chavasit, Director, and Drs. Emorn Wasantwisut and Pongtorn Sungpuag) for the completion of biochemical assays on biospecimens (plasma and breast milk) collected from participating study subjects, as described. The laboratory at INMU is well-established and capable of producing high-quality results, and our research group has had a long-standing relationship with the investigators there. Other than a list of sample id's, which provide no pertinent information about individual participants, the laboratory personnel at INMU will not have access to data from the main study.

11. <u>Other IRB Approvals</u>: Protocols for the main trial and its sub-studies will be reviewed and approved by the Bangladesh Medical Research Council, Dhaka, Bangladesh.

12. References:

1. Jiang T, Christian P, Khatry SK, Wu L, West KP Jr. Micronutrient deficiencies in early pregnancy are common, concurrent, and vary by season among rural Nepali women. J Nutr 2005; 135:1106-1112.

2. Pathak P, Kapil U, Kapoor KS, Saxena R, Kumar A, Gupta N, Dwivedi SN, Singh R, Singh P. Prevalence of multiple micronutrient deficiencies against pregnant women in a rural area of Haryana. Ind J Pediatr 2004; 71:1007-1014.

3. Dijkhuizen MA, Wieringa FT, West CE, Muherdiyantiningsih, Muhilal. Concurrent micronutrient deficiencies i lactating mothers and their infants in Indonesia. Am J Clin Nutr 2001;73:786-91.

4. Ramakrishnan U, Manjrekar R, Rivera J, Gonales-Cossio T, Martorell R. Micronutrients and pregnancy outcome: A review of the literature. Nutr Res 1999;19:103-159.

5. Friis H, Gomo E, Kaestel P, Ndhlovu P, Nyazema N, Krarup H, Michaelsen KF. HIV and other predictors of serum folate, serum ferritin, and hemoglobin in pregnancy: a cross-sectional study in Zimbabwe. Am J Clin Nutr 2001;73:1066-73.

6. Hollowell JG Jr, Hannon WH. Teratogen Update: Iodine deficiency, a community teratogen. Teratology 1997;55:389-405.

7. Christian P, Khatry SK, Katz J, Pradhan EK, LeClerq SC, Shrestha SR, Adhikari RK, Sommer A, West KP Jr. Effects of alternative maternal micronutrient supplements on low birth weight in rural Nepal. A double-blind randomized community trial. BMJ 2003; 326:571-576.

8. MRC Vitamin Study Group. Prevention of neural tube defects: results of the Medical Research Council Vitamin Study. Lancet 1991;338:131-7.

9. Czeizel AE, Dudas, I. Prevention of the first occurrence of neural-tube defects by periconceptional vitamin supplementation. NEJM 1992;327:1832-5.

10. Christian P, West KPO Jr, Khatry SK et al. Night blindness of pregnancy in rural Nepal – nutritional and health risks. Int J Epidemiol 1998;27:231-7.

Downloaded From: http://jama.jamanetwork.com/ by a Penn State Milton S Hershey Med Ctr User on 01/07/2015

11. West KP Jr., Katz J, Khatry SK, LeClerq S, Pradhan EK, Shrestha SR, Connor PB, Dali SM, Christian P, Pokhrel RP, Sommer A on behalf of the NNIPS-2 Group. Double blind, cluster randomized trial of low dose supplementation with vitamin A or B-carotene on mortality related to pregnancy. BMJ 1999;318:570-5.

12. Christian P, West KP Jr, Khatry SK, Katz J, LeClerq SC, Pradhan EK, Shrestha SR. Vitamin A or β-carotene reduces but does not eliminate maternal night blindness in Nepal. J Nutr 1998; 128:1458-1463.

13. Becker DV, Braverman LE, Delange F et al. Iodine supplementation for pregnancy and lactation-United States and Canada: recommendations of the american thyroid association. Thyroid 2006 Oct;16(10):949-51.

14. Villar J, Abdel-Aleem H, Merialdi M, et al. World Health Organization randomized trial of calcium supplementation among low calcium intake pregnant women. Am J Obstet Gynecol 2006;194:639-649.

15. Sloan NL, Jordan E, Winikoff B. Effects of iron supplementation on maternal hematologic status in pregnancy. Am J Public Health 2002;92:288-293.

16. Ramakrishnan U, Gonzalez-Cossio T, Neufield LM, Rivera J, Martorell R. Multiple micronutrient supplementation during pregnancy does not lead to greater infant birth size than does iron-only supplementation: a randomized controlled trial in a semirural community in Mexico. Am J Clin Nutr 2003; 77:720-5.

17. Friis H, Gomo E, Nyazema N, Ndhlovu P, Krarup H, Kaestel P, Michaelsen KF. Effect of multimicronutrient supplementation on gestational length and birth size: a randomized, placebo-controlled double-blind effectiveness trial in Zimbabwe. Am J Clin Nutr 2004; 80:178-84.

18. Kaestel P, Michaelsen KF, Aaby P, Friis H. Effects of prenatal multimicronutrient supplements on birth weight and perinatal mortality: a randomized, controlled, trial in Guinea-Bissau. European J Clin Nutr 2005; July 6 advance online publication.

19. Christian P, West KP Jr, Khatry SK, LeClerq SC, Pradhan EK, Katz J, Shrestha SR, Sommer A. Effects of micronutrient supplementation on fetal loss and infant mortality. A cluster-randomized trial in Nepal. Am J Clin Nutr 2003; 78:1194-1202.

20. Osrin D, Vaidya A, Shrestha Y et al. Effects of antenatal multiple micronutrient supplementation on birthweight and gestational duration in Nepal: double-blind, randomized controlled trial. Lancet 2005;365:955-62.

21. Christian P, Osrin D, Manandhar DS, Khatry SK, de L Costello AM, West KP Jr. Antenatal micronutrient supplements in Nepal. Lancet. 2005 Aug 27-Sep 2;366(9487):711-2.

22. S. Huffman, J. Habicht, N. Scrimshaw. Micronutrient supplementation in pregnancy. Lancet. 2005 366(9502): pp 2001.

23. Haider BA, Bhutta ZA. Multiple-micronutrient supplementation for women during

Downloaded From: http://jama.jamanetwork.com/ by a Penn State Milton S Hershey Med Ctr User on 01/07/2015

pregnancy. Cochrane Database Syst Rev. 2006 Oct 18;(4):CD004905.

24. Institute of Medicine. Dietary Reference Intakes for Vitamin A, Vitamin K, Arsenic, Boron, Chromium, Copper, Iodine, Iron, Manganese, Molybdenum, Nickel, Silicon, Vanadium, and Zinc. Washington, DC: National Academy Press, 2001.

25. Institute of Medicine. Dietary Reference Intakes for Vitamin C, Vitamin E, Selenium, and Carotenoids. Washington, DC: National Academy Press, 2000.

26. UNICEF/WHO/UNU. Composition of a multi-micronutrient supplement to be used in pilot programmes among pregnant women in developing countries. New York, UNICEF, 1999.

27. Christian P, Darmstadt GL, Wu L, Khatry SK, LeClerq SC, Katz J, West KP Jr, Adhikari RK. The impact of maternal micronutrient supplementation on early neonatal morbidity in rural Nepal: a randomized, controlled, community trial. Arch Dis Childhood (in press).

Appendix:

Table 1. Recommended Dietary Allowances and Recommended, Previously Used and Proposed JiVitA-3 Multiple Micronutrient Supplement Compositions

Table 2. Sample Size of Live Births and Pregnancies Required per Group

Downloaded From: http://jama.jamanetwork.com/ by a Penn State Milton S Hershey Med Ctr User on 01/07/2015

APPENDIX

Table 1. Recommended Dietary Allowances and Recommended, Previously Used and Proposed JiVitA-3 Multiple Micronutrient Supplement Compositions

	Reference: RDA[1] for Pregnancy/ Lactation	NNIPS-3[2]: Previously Used Formula	UNIMMAP[3]: Current UNICEF Formula	UNICEF/ WHO[4]: Emergency Guidelines	**JiVitA-3 Proposed (~1 RDA)[5]**
Vitamin A (µg RE)	750/770	1000	800	800	**770**
Vitamin D (µg)	5 (200 IU)	10	5	5	**5**
Vitamin E (mg)	15	10	10	15	**15**
Vitamin K (µg)	75/90	65	0	0	**0**
Folic Acid (µgDFE)	600	400	400	600	**600**
Thiamin (mg)	1.4	1.6	1.4	1.4	**1.4**
Riboflavin (mg)	1.4	1.8	1.4	1.4	**1.4**
Niacin (mg)	18	20	18	18	**18**
VitaminB-12 (µg)	2.6	2.6	2.6	2.6	**2.6**
Vitamin B-6 (mg)	1.9	2.2	1.9	1.9	**1.9**
Vitamin C (mg)	80/85	100	70	55	**85**
Iron (mg)	27	60	30	27	**27**
Zinc (mg)	12/11	30	15	10	**12**
Iodine (µg)	220	-	150	250	**220**
Copper (µg)	1000	2000	2000	1150	**1000**
Selenium (µg)	60	-	65	30	**60**
Magnesium (mg)	400/350	100	-	-	**-**

[1] Based on a multivolume set of publications entitled "Dietary Reference Intakes" by the Food and Nutrition Board of the Institute of Medicine, Washington DC, from 1997 to 2002 (24).
[2] Christian et al (7,19).
[3] Based on a UNICEF expert consultation in New York, NY in December 1999.
[4] World Health Organization, 2006 (26).
[5] As the comparison group, the antenatal standard of care daily supplement to be used in JiVitA-3 is proposed to contain iron (27 mg) as fumarate, reflecting the content of the UNIMMAP supplement, and folic acid (600 ug), reflecting the current RDA of the Dietary Reference Intake for folic acid set by the Institute of Medicine, Washington DC (24).

Downloaded From: http://jama.jamanetwork.com/ by a Penn State Milton S Hershey Med Ctr User on 01/07/2015

Table 2. Sample Size of Live Births and Pregnancies Required per Group (N_i)[1]

		**Percent Reduction in Infant Mortality (to six months)**				
**6- Mo IMR**	**No. of Births (Bx) and Pregnancies**	**10**	**15**	**20**	**25**	**30**
**50**	No. Bx (unadjusted)	28,806	12,574	6,943	4,360	2,969
	No. Bx (adjusted)[2]	34,912	15,222	8,040	5,278	3,594
	No. Pregnancies[3]	49,875	21,746	11,486	7,540	5,135
**55**	No. Bx (unadjusted)	26,059	11,377	6,282	3,947	2,688
	No. Bx (adjusted)	31,545	13,773	7,605	4,778	3,254
	No. Pregnancies	45,065	19,676	10,865	6,826	4,649
**60**	No. Bx (unadjusted)	23,769	10,379	5,733	3,602	2,453
	No. Bx (adjusted)	28,773	**12,565**	6,940	4,361	2,970
	No. Pregnancies	41,105	**17,950**	9,915	6,230	4,243
**65**	No. Bx (unadjusted)	21,831	9,535	5,267	3,309	2,255
	No. Bx (adjusted)	26,427	11,543	6,376	4,006	2,730
	No. Pregnancies	37,753	16,490	9,109	5,723	3,900
**70**	No. Bx (unadjusted)	20,171	8,811	4,868	3,060	2,085
	No. Bx (adjusted)	24,418	10,666	5,893	3,705	2,524
	No. Pregnancies	34,883	15,238	8,419	5,293	3,607

[1] Sample size estimates based on presented ranges of infant mortality in the standard of care control group and plausible percent reductions in 6-month infant mortality that could result from maternal multiple micronutrient supplementation, accepting probabilities of Type I and Type II errors of 0.05 and 0.20, respectively.
[2] Adjusted for an estimated design effect = 1.15 and a 5% loss to follow-up.
[3] Based on an estimated pregnancy loss rate of 30% due to miscarriage (~12%), menstrual regulation (~14%) and still birth (~4%). Reference: JivitA-1 trial (unpublished, 2006)

Downloaded From: http://jama.jamanetwork.com/ by a Penn State Milton S Hershey Med Ctr User on 01/07/2015
